# Coordination and Homologation of CO at Al(I): Mechanism and Chain Growth, Branching, Isomerization, and Reduction

**DOI:** 10.1021/jacs.2c05228

**Published:** 2022-07-05

**Authors:** Andreas Heilmann, Matthew M. D. Roy, Agamemnon E. Crumpton, Liam P. Griffin, Jamie Hicks, Jose M. Goicoechea, Simon Aldridge

**Affiliations:** Inorganic Chemistry Laboratory, Department of Chemistry, University of Oxford, South Parks Road, Oxford OX1 3QR, U.K.

## Abstract

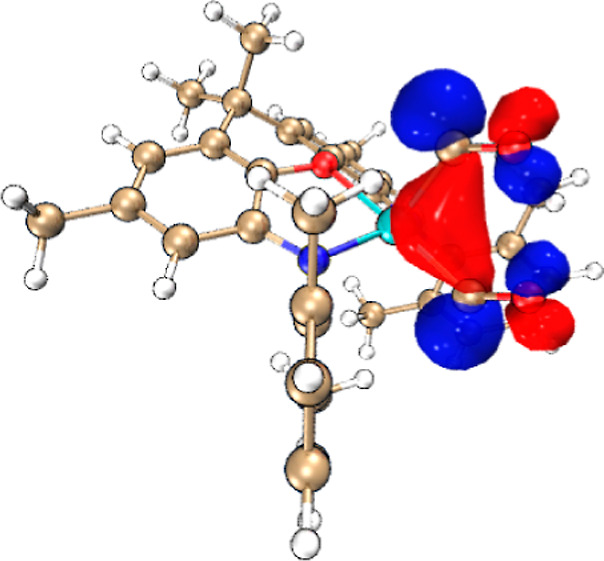

Homologation of carbon monoxide is central to the heterogeneous Fischer–Tropsch process for the production of hydrocarbon fuels. C–C bond formation has been modeled by homogeneous systems, with [C_*n*_O_*n*_]^2–^ fragments (*n* = 2–6) formed by two-electron reduction being commonly encountered. Here, we show that four- or six-electron reduction of CO can be accomplished by the use of anionic aluminum(I) (“aluminyl”) compounds to give both topologically linear and branched C_4_/C_6_ chains. We show that the mechanism for homologation relies on the highly electron-rich nature of the aluminyl reagent and on an unusual mode of interaction of the CO molecule, which behaves primarily as a Z-type ligand in initial adduct formation. The formation of [C_6_O_6_]^4–^ from [C_4_O_4_]^4–^ shows for the first time a solution-phase CO homologation process that brings about chain branching via complete C–O bond cleavage, while a comparison of the linear [C_4_O_4_]^4–^ system with the [C_4_O_4_]^6–^ congener formed under more reducing conditions models the net conversion of C–O bonds to C–C bonds in the presence of additional reductants.

## Introduction

The assembly of complex molecules from carbon monoxide via C–C bond formation represents a fundamental chemical challenge that has key relevance to the production of hydrocarbon fuels via the heterogeneous Fischer–Tropsch process.^1^ Although employed industrially since 1925, the mechanism for the conversion of mixtures of CO and H_2_ to short-to-medium-chain alkane products through the formation of C–C bonds remains the subject of significant debate.^2^ C=C bond formation to yield alkenes represents a competing homologation process, and the product mixture typically also includes oxygenated species along with methane—an undesirable reduction product produced without the accompanying C–C bond formation. The idealized ratio of H_2_/CO (ca. 2:1; eq 1, typically with 10 < *n* < 20) reflects the importance of the reductant (H_2_) in generating the desired alkane products. The much lower proportion of dihydrogen in the coal-derived synthesis gas (“Syn Gas”) feedstock is typically rectified through application of the water gas shift reaction.^3^
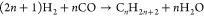
1

**Figure 1:**
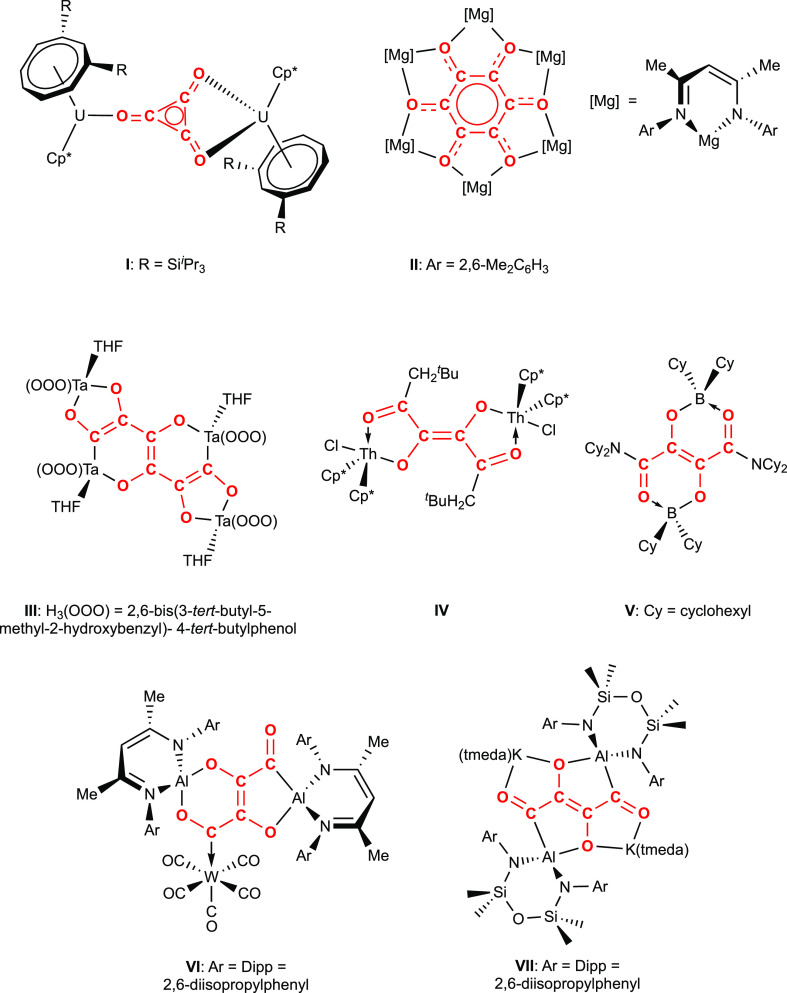
Selected examples of CO homologation relevant to the current study.

The amenability of homogeneous systems to small-molecule characterization techniques has driven the investigation of organometallic compounds capable of modeling the homologation of CO via C–C bond formation.^4^ Prominent examples include *d*-block metal compounds (mirroring the use of heterogeneous transition-metal catalysts such as iron and cobalt in the Fischer–Tropsch process),^5−17^ with examples derived from low-valent *s*-, *p*-,^18−33^ and *f*-block compounds^34−50^ having also been reported (e.g., **I**–**VII**, Figure 1). Prevalent among the homologation products formed via reduction processes are cyclic systems of the type [C_*n*_O_*n*_]^2–^ (*n* = 3, 4, and 6) and the related ethynediolate system [C_2_O_2_]^2–^,^6−8,11,18,20−23,29,40,41,44−48^ formed by a formal two-electron reduction process, although a number of systems featuring longer linear chains of carbon atoms have also been reported (Figure 1).^14,17,22,25,31,33,34,36,37,42,49^ Within this sphere, a small number of studies have emerged, which demonstrate the potential for control of homologation processes at a constant oxidation level: the selective formation of [C_*n*_O_*n*_]^2–^ (*n* = 2, 3, or 4) by the cooperative action of U(III) or Mg(I) centers has been shown to be influenced by the steric bulk of ancillary ligands,^21,48^ and the stepwise growth of C_3_ chains by the addition of CO has been demonstrated at *d*-block metal systems derived from metal carbonyl precursors.^17^ Very recently, the formation of C_4_ or C_5_ chains using aluminum(I) systems has been demonstrated, with the product formed appearing to reflect the nuclearity of the metal reagent.^33^

**Figure 2:**
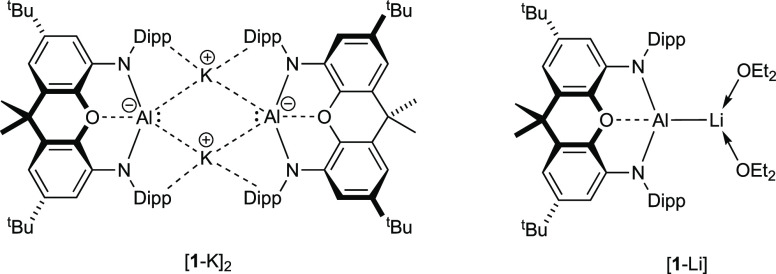
Aluminyl compounds central to the current study: dimeric potassium aluminyl compound K_2_[(NON)Al]_2_, [**1**-K]_2_, and monomeric lithium analogue (Et_2_O)_2_LiAl(NON), [**1**-Li(OEt_2_)_2_].

We have been developing the chemistry of electron-rich aluminum (“aluminyl”) compounds—stabilized by NON supporting ligands (4,5-bis(2,6-diisopropylanilido)-2,7-di-*tert*-butyl-9,9-dimethylxanthene; Figure 2).^51,52^ Such systems have been shown to be capable of the cleavage of C–H,^51^ C–C,^53^ and C–O bonds in arene substrates^54^ and to allow access to reactive terminal imide species which can cleave the CO bond in carbon monoxide.^55^ Systems such as K_2_[(NON)Al]_2_ ([**1**-K]_2_) have also shown strongly reducing capabilities toward oxygen-containing substrates,^56^ and here, we report on the reactivity of aluminyl compounds toward CO. We show that the homologation to give the known [C_4_O_4_]^4–^ fragment occurs with both mono- and dinuclear systems, a finding rationalized computationally through a rate-determining C=C bond-forming dimerization process. The initial interaction of the CO molecule at aluminum is unusual, in that it behaves primarily as a Z-type ligand, reflecting the highly electron-rich nature of the aluminyl reagent. Subsequent formation of [C_6_O_6_]^4–^ from [C_4_O_4_]^4–^ shows for the first time a solution-phase CO homologation process that brings about chain branching via complete C–O bond cleavage. In addition, comparison of the linear [C_4_O_4_]^4–^ system with the (unprecedented) [C_4_O_4_]^6–^ congener formed under more reducing conditions models the conversion of C–O bonds to C–C bonds in the presence of additional reductants.

## Results and Discussion

### Synthesis of [C_4_O_4_]^4–^ and [C_6_O_6_]^4–^ via CO Homologation

**Figure 3:**
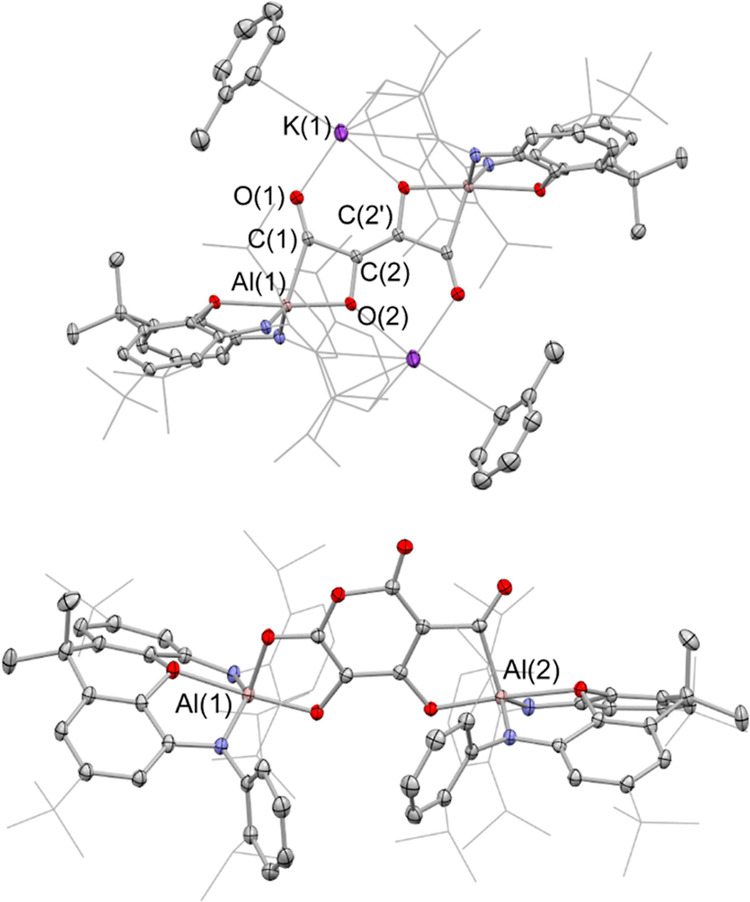
Molecular structures of (upper) [K(C_7_H_8_)]_2_[{(NON) Al}_2_(C_4_O_4_)], [**2**-K(C_7_H_8_)]_2_, and (lower) the anionic component of the asymmetric unit of K[K(THF)_2_][{(NON)Al}_2_(C_6_O_6_)], **3**, in the solid state as determined by X-ray crystallography. Hydrogen atoms omitted and selected groups represented in the wireframe format for clarity; thermal ellipsoids drawn at the 50% probability level. Key metrical parameters are listed in Table 1.

**Scheme 1:**
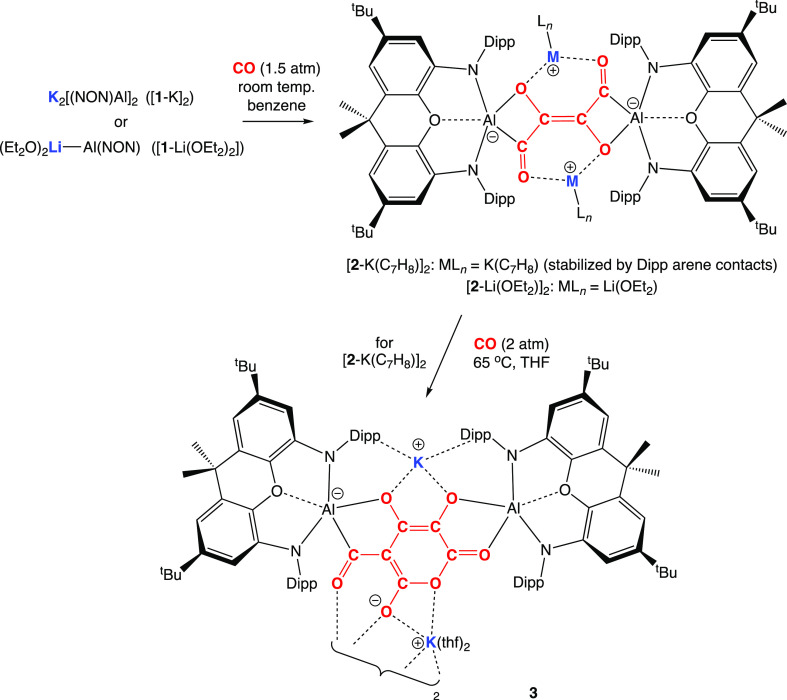
Homologation of CO by Aluminyl Compounds Yielding Products Containing [C_4_O_4_]^4–^ or [C_6_O_6_]^4–^ Fragments Four-electron reduction of CO by [**1**-K]_2_ or 2 equiv of [**1**-Li(OEt_2_)_2_] to give [**2**-K(C_7_H_8_)]_2_/[**2**-Li(OEt_2_)]_2_, featuring the topologically linear [C_4_O_4_]^4–^ fragment; onward reaction of [**2**-K(C_7_H_8_)]_2_ with CO to yield the [C_6_O_6_]^4–^ system, **3**.

The reaction of potassium aluminyl compound [**1**-K]_2_^51^ with CO (ca. 1.5 atm) in benzene over 16 h at ca. 35 °C (followed by recrystallization from toluene) leads to the isolation of the orange crystalline product [K(C_7_H_8_)]_2_[{(NON)Al}_2_(C_4_O_4_)], [**2**-K(C_7_H_8_)]_2_, which has been characterized by standard spectroscopic and micro-analytical methods (Scheme 1 and Figure 3). [**2**-K(C_7_H_8_)]_2_ can be shown by X-ray crystallography to feature a centrosymmetric structure in which two [(NON)Al] units are bridged by a [C_4_O_4_] fragment and two K^+^ counterions (which each interact with the π system of a Dipp group of one NON ligand and an additional toluene molecule; Figure 3).

Overall charge balance implies that the bridging unit is [C_4_O_4_]^4–^, a finding consistent with the presence in the (dimeric) aluminyl starting material of two Al(I) reductants, that is, with four-electron reduction of four molecules of CO. To a first approximation, C–C distances within the [C_4_O_4_]^4–^ unit reflect the presence of two C–C single bonds (1.507(1) Å) and a central C=C double bond (1.373(1) Å), while the C–O distances (1.383(1)/1.233(1) Å) are consistent with C–O single bonds linked to the central carbon atoms [i.e., C(2)] and C=O double bonds involving the Al-bound carbons C(1).

Four-carbon homologation has some literature precedent. In terms of a metal-bound ligand system, the C/O skeleton bears some resemblance connectivity-wise to the enedione diolate [R_2_C_4_O_4_]^2–^ system reported by Marks and co-workers, derived from the insertion of two molecules of CO into a thorium(IV) alkyl bond (**IV**)^34,36^ and to the mixed d-block/Al systems reported by Crimmin et al. (**VI**).^17^ Structurally, however, it is most closely related to Stephan’s B/N-functionalized system Cy_2_N{C_4_O_4_(BCy_2_)_2_}NCy_2_ derived from the trapping of CO by a combination of LiNCy_2_ and ClBCy_2_, and which also features a C(=O)–C(−O)=C(−O)–C(=O) skeleton (**V**).^31^ In addition, a similar C_4_ system was reported very recently by Coles and co-workers (**VII**).^33^

The isolated yield of [**2**-K(C_7_H_8_)]_2_ obtained from [**1**-K]_2_ and CO is low (ca. 10%), and in situ ^1^H NMR monitoring is consistent with the formation of other (NON)Al-containing species under these reaction conditions. Hypothesizing that this is due to the formation of other homologation products of the type [C_*n*_O_*n*_]^4–^, we sought (i) to probe the reactivity of isolated samples of [**2**-K(C_7_H_8_)]_2_ with CO under more forcing conditions, to see if homologation could be driven toward longer C–C-bonded chains and (ii) to investigate if greater control of the delivery of the carbon monoxide (and with it the product distribution) could be achieved by the use of alternative CO-containing reagents. Targeting the second of these objectives, we employed Fe(CO)_5_ as a labile (and readily soluble) source of the CO molecule in reactions with [**1**-K]_2_. Reaction with 4 equiv of Fe(CO)_5_ in benzene-*d*_6_ in the presence of the crown ether 12-crown-4 (to aid crystallization) leads to the formation of [**2**-K(12-c-4)]_2_, the solid-state structure of which is very closely related to that of [**2**-K(C_7_H_8_)]_2_ (see the Supporting Information). While the potential involvement of the iron center in Fe(CO)_5_ in this chemistry cannot be ruled out, it is noteworthy that conversion to the same [C_4_O_4_]^4–^ fragment via this approach is markedly higher (20–30%) than that obtained using gaseous CO.

The possibility for assembling [C_*n*_O_*n*_]^4–^ chains with *n* > 4 was probed via the reaction of [**2**-K(C_7_H_8_)]_2_ with CO at higher temperatures/pressures. Use of a 2 atm pressure in THF-*d*_8_ at ca. 65 °C over 4 d leads to quantitative conversion by ^1^H NMR (through at least three intermediate species) to a single product featuring two inequivalent NON ligands, which can be shown by X-ray crystallography to be K[K(THF)_2_] [{(NON)Al}_2_(C_6_O_6_)], (**3**; Figure 3). **3** features a [C_6_O_6_]^4–^ fragment formed by the assimilation of two further equivalents of CO, which presents distinct (O,O and C,O) coordination modes at the two aluminum centers. The geometry of the [C_6_O_6_]^4–^ group is unique, featuring a six-membered pyran core, with the mono-branched nature of the six-carbon chain implying that C–C bond formation is also accompanied by complete cleavage of one of the C–O bonds. While the homologation of CO by highly reducing molecular species to give linear or cyclic products has a literature precedent, to our knowledge, the formation of *branched* carbon chains in this fashion, with the accompanying cleavage of the exceptionally strong C–O bond, is unprecedented. Examples of branched-chain carbon skeletons have been reported previously—but only via subsequent derivatization reactions of linear CO-derived chains with electrophiles such as CO_2_ or MeI.^50^

### Mechanistic Studies: CO Coordination and C–C/C=C Bond Formation

While the two-electron reduction of CO by a number of main group compounds to give systems of the type [C_*n*_O_*n*_]^2–^ has been reported,^18,20−23,29^ four-electron processes yielding the topologically linear [C_4_O_4_]^4–^ fragment have a more limited literature precedent.^31,33^ We were therefore keen to probe the mechanism of this transformation through both experimental and quantum chemical methods. As a key mechanistic principle, we first set out to determine whether the dimeric nature of the aluminyl reagent, [**1**-K]_2_, is critical in driving the formation of the (four-electron reduction) product, [C_4_O_4_]^4–^.

[**1**-K]_2_ has been shown by DOSY NMR measurements to retain a dinuclear structure in solution in arene solvents. With this in mind, we targeted the reactivity of CO with related aluminyl compounds known to possess a mononuclear structure. The reaction of the (monomeric) lithium aluminyl complex (Et_2_O)_2_LiAl(NON) ([**1**-Li(OEt_2_)_2_])^57^ with CO was therefore probed under similar conditions. Intriguingly, this chemistry leads to the formation of a very similar dinuclear product, [**2**-Li(OEt_2_)]_2_, featuring an analogous [C_4_O_4_]^4–^ fragment, and two encapsulated Li^+^ counterions each ligated by two of the oxygen atoms of the [C_4_O_4_]^4–^ unit and an additional molecule of diethyl ether. The structural metrics relating to the Al_2_(C_4_O_4_) unit (Table 1 and the Supporting Information) are very similar to those measured for [**2**-K(C_7_H_8_)]_2_, being consistent with a centrosymmetric four-carbon chain incorporating two C–C and one C=C bonds. Moreover, the formation of [**2**-Li(OEt_2_)]_2_ from [**1**-Li(OEt_2_)_2_] in this manner (in greater conversion to [**2**-K(C_7_H_8_)]_2_, 40 vs 10%) suggests that the state of aggregation of the aluminyl reagent is not critical in determining the stoichiometry of this carbon-containing product. This observation is consistent with the mechanism proposed by Marks and co-workers for the formation of [Cp*_2_Th{OC(CH_2_^*t*^Bu)C(O)}]_2_, in which the central C=C bond is postulated as being constructed in the final mechanistic step through the dimerization of two mono-metallic carbene units (themselves accessed by bending of a coordinated ketene ligand).^36^ Coles has also proposed that the dimerization of a dioxocarbene moiety is the final step in the formation of an ethenetraolate ligand, [C_2_O_4_]^4–^.^58^

**Table 1:** Key Metrical Parameters for Aluminum Complexes Containing [C_4_O_4_]^*n*−^ Ions (*n* = 2, 4, and 6)

		[C_4_O_4_]^4–^		[C_4_O_4_]^6–^	[C_4_O_4_]^2–^
d/Å	[**2**-K(C_7_H_8_)]_2_	[**2**-K(THF)]_2_	[**2**-Li(OEt_2_)]_2_	[**4**-K_2_(BEt_3_)]_2_	5
C–C	1.507(1)	1.525(4)	1.482(5)	1.481(3)	1.438(2)
	1.373(1)	1.489(3)	1.357(4)	1.377(4)	1.461(2)
		1.376(3)			
C–O	1.233(1)	1.213(4)	1.246(5)	1.382(3)	1.257(2)
	1.383(1)	1.247(3)	1.391(4)	1.440(3)	1.265(2)
		1.373(3)			
		1.381(3)			
Al–C	2.038(1)	2.053(2)	2.063(4)	2.008(2)	n/a
		2.041(3)			
Al–O	1.871(1)	1.872(2)	1.877(2)	1.874(2)	1.933(1)
		1.860(2)			1.994(1)

**Figure 4:**
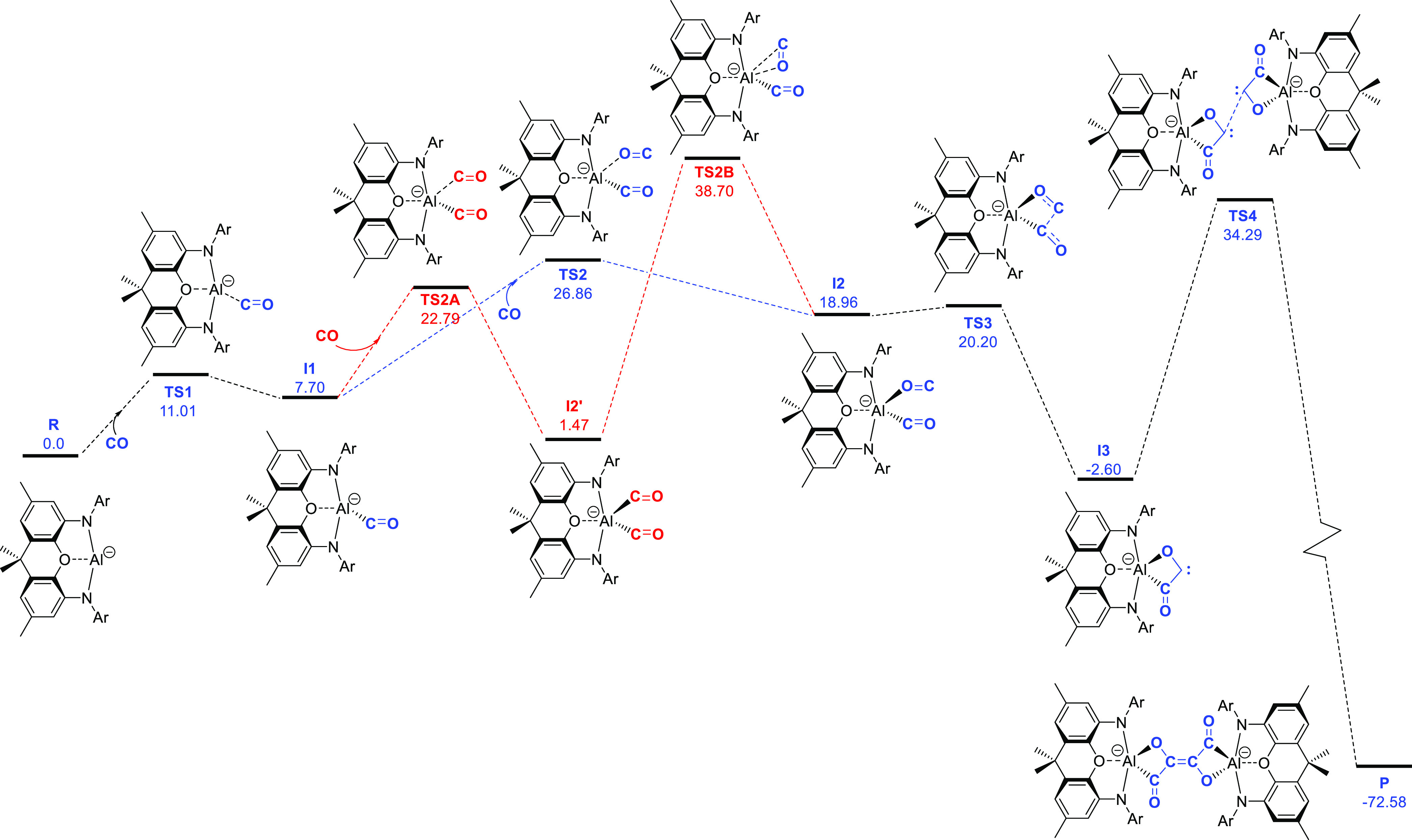
Proposed mechanism for CO homologation. The lowest energy pathway (blue) involves iso-carbonyl binding of one molecule of CO (**I2**); the alternative (higher barrier) pathway shown in red involves binding of the second CO through carbon (**I2′**) and then isomerization to **I2**. (Calculations carried out at the B3LYP/def2-TZVP//B3LYP/def2-SVP level with solvation modeled with CPCM (benzene) and ^*t*^Bu and ^*i*^Pr groups replaced by Me for computational efficiency.)

In the case of the (NON)Al-derived systems presented here, this hypothesis is consistent with the results of mechanistic calculations carried out using density functional theory—for a model system in which the backbone ^*t*^Bu and Dipp ^*i*^Pr groups are replaced by Me and the cation is omitted for computational efficiency (Figure 4). The rate-determining step for the lowest energy pathway is found to involve dimerization of two carbene fragments, with these each being derived from the coupling of two molecules of CO. Kinetically, the transition state energy for the C=C forming step is located ca. 34 kcal mol^–1^ above the starting materials (and ca. 37 kcal mol^–1^ above the preceding singlet carbene intermediate). This barrier height reflects, at least in part, the sterically encumbered (and anionic) nature of the two NON-supported fragments being brought together and presumably underpins the relatively low yields associated with systems such as [**2**-K(C_7_H_8_)]_2_ (and the possibility for forming longer C-based chains via carbene/CO coupling).^33^

**Figure 5:**
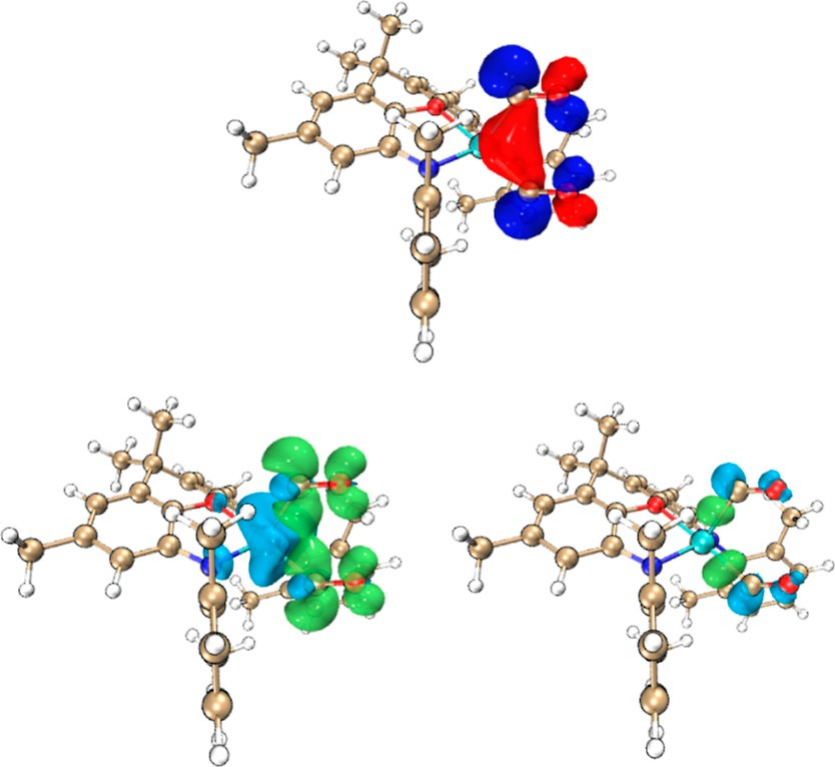
(Upper) HOMO of the model *C*-bound bis(carbonyl) adduct [(NON′)Al(CO)_2_]^−^ (shading denotes phasing of the wavefunction); (lower) predominant orbital interactions determined by ETS-NOCV methods for the binding of the two carbonyl ligands (shading denotes deformation densities: blue/green—depletion/enhancement of electron density).

In terms of the initial interaction of the CO molecule(s) with the (model) [(NON′)Al]^−^ unit, we find that the end-on approach of CO perpendicular to the AlN_2_ plane in a manner analogous to that determined for CO-adducts of isoelectronic silylene systems, X_2_Si·CO,^59^ does not correspond to a local minimum on the potential energy surface. Rather, reflecting the nucleophilic nature of the aluminyl reagent, the approach of the carbon monoxide unit gives rise to a bent Al–C–O unit, and the predominant orbital interaction involves electron donation *from* Al to the π* orbital of CO. For the model C-bound bis (carbonyl) adduct [(NON′)Al(CO)_2_]^−^, the HOMO (Figure 5) defines a three-center interaction involving the aluminum-centered lone pair and the π* orbitals of the two carbonyl ligands. Moreover, extended transition state with natural orbitals for chemical valence (ETS-NOCV) analysis determines that this three-center “back-bonding” interaction accounts for the majority (261 kcal mol^–1^) of the orbital interaction energy (Δ*E*_orb_).^60^ By contrast, the complementary three-center interaction involving donation from the CO lone pairs to the Al-centered p_π_-orbital, accounts for only 25 kcal mol^–1^. By means of comparison, a related ETS-NOCV analysis on the mono-carbonyl adduct of a cationic borole (in which the CO ligand behaves primarily as a σ-donor) yields 26 kcal mol^–1^ (back-bonding to CO) and 106 kcal mol^–1^ (sigma donation from CO).^61^ As such, the primary role of the CO donors in [(NON′)Al(CO)_2_]^−^ is strongly suggested to be as Z-type ligands.

While the bis-*C*-ligated adduct [(NON′)Al(CO)_2_]^−^ (**I2′**) represents the lowest energy form of the dicarbonyl species, onward transformation into carbene intermediate **I3** necessarily involves rotation of one of the CO ligands to generate the isomeric species [(NON′)Al(CO)(OC)]^−^ (**I2**) featuring one isocarbonyl donor.^62^ The barrier to this isomerization within the coordination sphere of aluminum (ca. 40.2 kcal mol^–1^) is found to be significantly higher than that for simple dissociation/re-association. Moreover, while **I2** is ca. 17.5 kcal mol^–1^ higher in energy than **I2′**, it is capable of undergoing a very facile C–C coupling to generate carbene intermediate **I3**, which is lower in energy than either of the carbonyl adducts.

**Figure 6:**
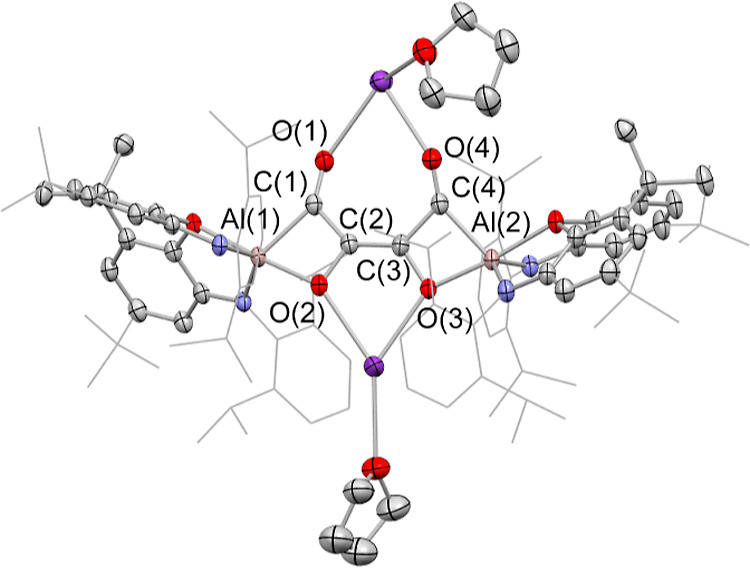
Molecular structure of [K(THF)]_2_[{(NON)Al}_2_(C_4_O_4_)], [**2**-K(THF)]_2_, in the solid state as determined by X-ray crystallography, showing dimeric unit analogous to [**2**-K(C_7_H_8_)]_2_. Further aggregation into “dimer of dimers” shown in Figure S16 (Supporting Information). Hydrogen atoms omitted and selected groups represented in a wireframe format for clarity; thermal ellipsoids drawn at the 50% probability level. Key metrical parameters are listed in Table 1.

**Scheme 2:**
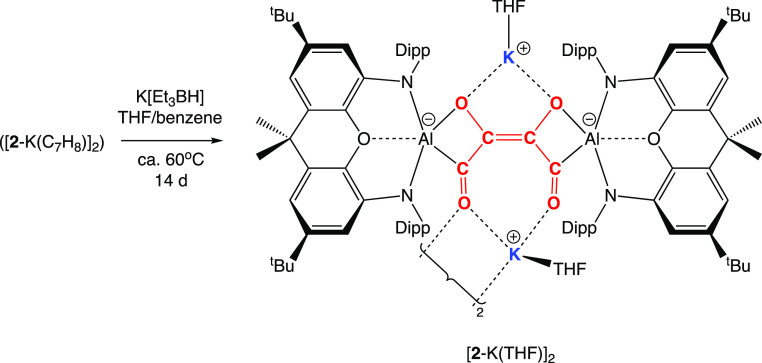
Isomerization of [C_4_O_4_]^4–^: *anti* to *syn* Isomerization of the [C_4_O_4_]^4–^ Fragment in the Presence of K[Et_3_BH] in Benzene/THF at ca. 60 °C

Interestingly, although C=C bond formation represents the key thermodynamic driving force for the overall homologation reaction (with the final dimerization process being energetically downhill by ca. 70 kcal mol^–1^), experimental evidence for the *lability* of the central C=C bond in [C_4_O_4_]^4–^ systems can be obtained from the reactions of isolated samples of [**2**-K(C_7_H_8_)]_2_ with alkali metal hydroborate reagents. In the case of the reaction with Li[^*s*^Bu_3_BH] in benzene/THF, simple cation metathesis is observed, generating [Li(THF)]_2_ [{(NON)Al}_2_(C_4_O_4_)], [**2**-Li(THF)]_2_, the structure of which closely resembles both [**2**-K(C_7_H_8_)]_2_ and [**2**-Li(OEt_2_)]_2_ (see the Supporting Information). However, thermolysis with K[Et_3_BH] at ca. 65–70 °C in the same solvent system leads to 70% conversion over a period of 14 d to a new species, the apparently analogous potassium salt [**2**-K(THF)]_2_. The structure of this species, however, can be shown by X-ray crystallography to feature the alternative *syn* disposition of substituents about the central C=C double bond (Scheme 2 and Figure 6).^33^ The C=C/C–C and C=O/C–O bond lengths within the carbon chain are not significantly different from the *anti*-form, but the [**2**-K(THF)]_2_ units are linked in the solid state via bridging K^+^ cations into tetra-aluminum “dimer of dimers” (see the Supporting Information). The lower solubility of this aggregate presumably drives the *anti*-to-*syn* isomerization process.

Kinetically, both heat and the presence of additional K^+^ cations can be shown to be necessary for the transformation to occur. C=O/C=C conjugation within the [C_4_O_4_]^4–^ unit (manifested through resonance structures featuring a central C(2)–C(2′) single bond) presumably lowers the barrier to rotation about the central C=C double bond and allows mechanistically for the *anti*-to-*syn* isomerization process.

### [C_4_O_4_]^6–^ and [C_4_O_4_]^2–^ Systems: Synthesis and Structural Comparisons

**Figure 7:**
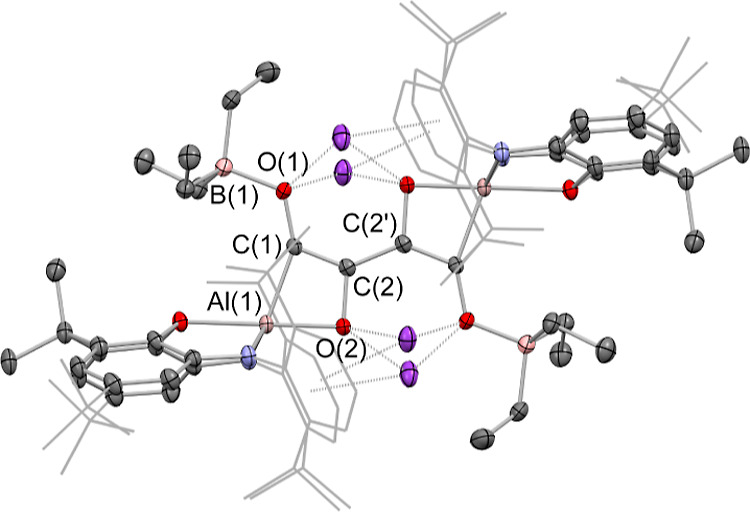
Molecular structure of [**4**-K_2_(BEt_3_)]_2_ in the solid state as determined by X-ray crystallography. Hydrogen atoms omitted and selected groups represented in a wireframe format for clarity; thermal ellipsoids drawn at the 50% probability level. Key metrical parameters are listed in Table 1.

**Scheme 3:**
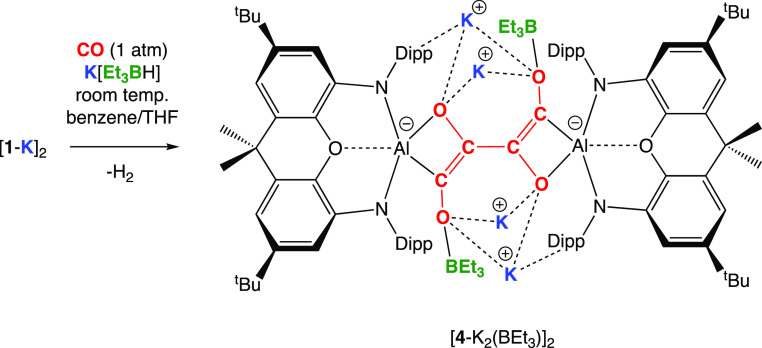
Synthesis of a Dialuminum System Containing the [C_4_O_4_]^6–^ Fragment: Synthesis of K_4_[{(NON)Al}_2_(C_4_O_4_) (BEt_3_)_2_], [**4**-K_2_(BEt_3_)]_2_, via the Reaction of [**1**-K]_2_ with CO in the Presence of the Hydride Source K[Et_3_BH]

While *isolated* samples of [**2**-K(C_7_H_8_)]_2_ are resistant to further reduction by the hydride component of Li[^*s*^Bu_3_BH] or K[Et_3_BH], the addition of K[Et_3_BH] to a mixture of [**1**-K]_2_/CO in benzene/THF in situ allows a more reduced product to be obtained. In this case, the reaction leads to evolution of dihydrogen (as judged by ^1^H NMR spectroscopy) and the formation of the further reduced species K_4_[{(NON)Al}_2_(C_4_O_4_)(BEt_3_)_2_] ([**4**-K_2_(BEt_3_)]_2_), containing an unprecedented formally hexa-anionic [C_4_O_4_]^6–^ chain (Scheme 3 and Figure 7). Mechanistically, the fact that *isolated* samples of [**2**-K(C_7_H_8_)]_2_ are not reduced (either chemically or electrochemically) suggests that the formation of [**4**-K_2_(BEt_3_)]_2_ involves a reduction event which occurs *prior* to the dimerization of the carbene intermediate **I3**.

[**4**-K_2_(BEt_3_)]_2_ is extremely reactive, particularly in solution, and has limited solubility in compatible solvents; as such, it can be characterized by ^1^H NMR spectroscopy and X-ray crystallography only. The centro-symmetric structure revealed crystallographically reveals four K^+^ cations encapsulated by a combination of O-atom and aryl π system ligation. Two molecules of triethylborane are also incorporated into the molecular framework, bound to the terminal O-atoms of the [C_4_O_4_]^6–^ chain. The central C–C distance is consistent with a single bond (1.481(3) Å), and the two terminal CC linkages feature bond lengths (1.377(4) Å) reflective of a C=C bond within an enolate functionality. The C–O distances[(1.382(3) and 1.440(3) Å] are consistent with single bonds. As such, the structural metrics for [**4**-K_2_(BEt_3_)]_2_ are consistent with a system based around a butadiene-tetraolate skeleton, C(−O)=C(−O)–C(−O)=C(−O).

**Figure 8:**
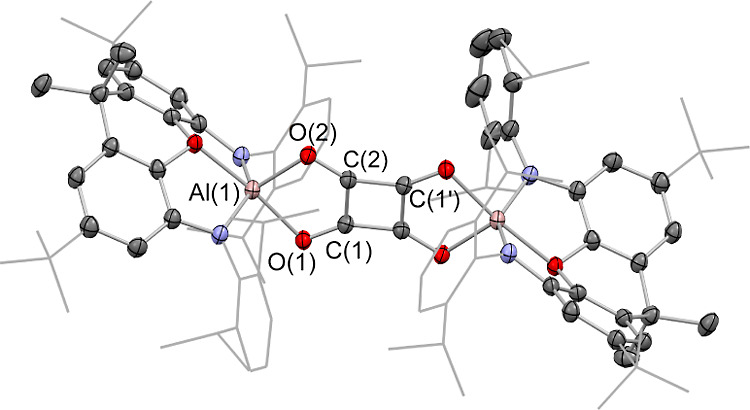
Molecular structure of the dialuminum-supported [C_4_O_4_]^2–^ compound {(NON)Al}_2_(C_4_O_4_), **5**, in the solid state as determined by X-ray crystallography. Hydrogen atoms omitted and selected groups represented in a wireframe format for clarity; thermal ellipsoids drawn at the 50% probability level. Key metrical parameters are listed in Table 1.

To allow for a complete structural comparison of the series of systems of the type [C_4_O_4_]^*n*−^ (*n* = 2, 4, 6) within the same aluminum coordination sphere, we also targeted the corresponding [C_4_O_4_]^2–^ (squarate) system. Such systems have been synthesized in the past by homologation of CO using metal centers (or combinations of metal centers) capable of effecting a two-electron reduction.^20,44^ In our hands, however, dimeric Al(II) systems such as (NON)Al–Al(NON)^51^ do not react with CO under either thermal or photolytic conditions, presumably reflecting the stronger nature of the metal–metal bond compared, for example, to dimeric Mg(I) reductants.^20−22^ For structural comparison, we therefore examined alternative synthetic approaches involving the reactions of pre-formed squarate salts M_2_[C_4_O_4_] with the readily available Al(III) iodide precursor (NON)AlI. In the event, (NON)Al(C_4_O_4_)Al(NON) (**5**) is most easily accessed by combining the disilver salt of squaric acid, Ag_2_[C_4_O_4_], with (NON)AlI in Et_2_O. **5** has been characterized by standard spectroscopic and analytical methods and by X-ray crystallography (Figure 8). Its molecular structure features a planar carbocyclic [C_4_O_4_]^2–^ ligand bridging between the two aluminum centers. The carbon-containing fragment closely resembles those reported previously,^20,44^ featuring C–C and C–O distances of 1.438(2)/1.461(2) and 1.257(2)/1.265(2) Å, respectively, consistent with the presence of a delocalized π system involving all four CO units, albeit with the fourfold symmetry of the free squarate ion being disrupted by chelation to the aluminum centers. The two different Al–O distances [1.933(1) and 1.994(1) Å] reflect a geometry at aluminum, which is between square pyramidal and trigonal bipyramidal (τ = 0.55), with the longer bond length being associated with the axial O-donor (assuming the TBP limit).

**Figure 9:**
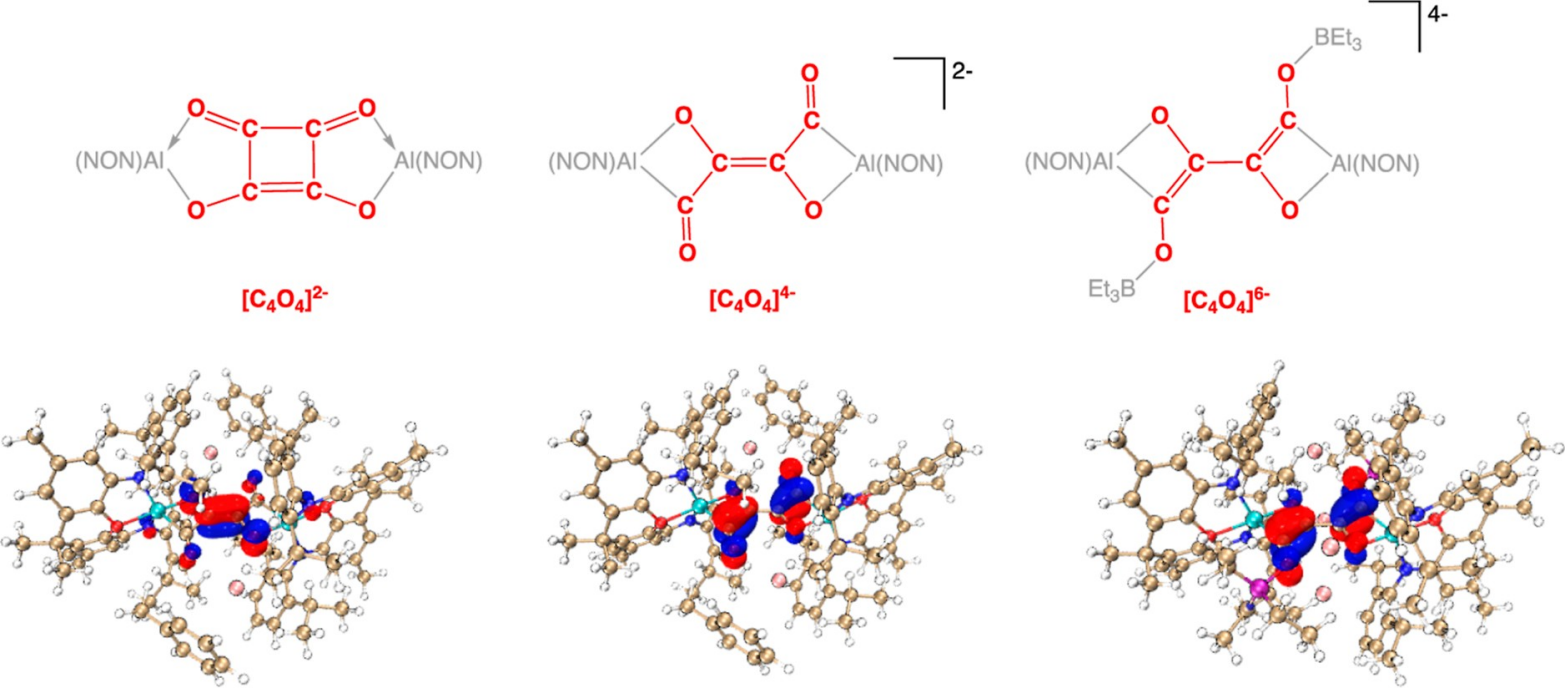
Comparison of the structures of the [C_4_O_4_]^*n*−^ fragments within **5**, [**2**-K(C_7_H_8_)]_2_, and [**4**-K_2_(BEt_3_)]_2_: (upper) important resonance structures of [C_4_O_4_]^2–^, [C_4_O_4_]^4–^, and [C_4_O_4_]^6–^ fragments; (lower) Key frontier orbitals: HOMO-1 and LUMO of [**2**-K(C_7_H_8_)]_2_ and HOMO of [**4**-K_2_(BEt_3_)]_2_.

The availability of structural data for **5**, [**2**-K(C_7_H_8_)]_2_, and [**4**-K_2_(BEt_3_)]_2_ provides a unique opportunity to probe the effects on the carbon/oxygen aggregate of stepwise (formal) reduction of the carbocyclic fragment (Figure 9)—notwithstanding the fact that (experimentally) both chemical oxidation and electrochemical oxidation or reduction of [**2**-K(C_7_H_8_)]_2_ are not facile. The two π-electron squarate dianions have been described as possessing a “moderate” aromaticity,^63^ a factor which underpins the prevalence of this planar carbocycle in four-carbon systems derived from two-electron reduction processes of CO.^20,44^ In the case of **5**, DFT calculations imply that an alternative (linear) isomer featuring a chain of four −C(=O)– units (akin to a doubly oxidized form of [**2**-K(C_7_H_8_)]_2_) is not an energetic minimum, rearranging to give a system featuring a bridging ethynediolate fragment ([OCCO]^2–^) and two aluminum-bound CO ligands (which even then lies ca. 10.8 kcal mol^–1^ above the squarate form; see the Supporting Information). The topologically linear butenedione diolate structure associated with the more heavily reduced [C_4_O_4_]^4–^ tetra-anion avoids the formation of a Hückel 4π anti-aromatic carbocycle derived from the addition of an additional pair of electrons to the LUMO of the squarate system. An alternative carbocyclic isomer featuring a more pronounced rectangular “squarate” core (akin to the Jahn–Teller distorted structure of the related 4π electron system cyclobutadiene)^64^ is found to lie significantly higher in energy (by >48 kcal mol^–1^).

The HOMO-1 of [**2**-K(C_7_H_8_)]_2_ [Figure 9 (lower)] corresponds to a π-bonding orbital across the central CC linkage of the [C_4_O_4_]^4–^ ligand. The formal addition of a further pair of electrons would then generate the [C_4_O_4_]^6–^ hexa-anion, which adopts a butadienetetraolate structure via reduction of the enedione component to the corresponding bis(enolate). The structural changes associated with the CC and CO linkages on transitioning from [C_4_O_4_]^4–^ to [C_4_O_4_]^6–^ are consistent with the form of the LUMO of [**2**-K(C_7_H_8_)]_2_ (and the HOMO of [**4**-K_2_(BEt_3_)]_2_), which features out-of-phase π interactions in the terminal CO and central CC units (Figure 9).

The overall transition from [C_4_O_4_]^2–^ to [C_4_O_4_]^4–^ to [C_4_O_4_]^6–^ is characterized by net bond cleavage (C–C/C–O bonds: 11 to 10 to 9), consistent (at a simplistic level) with successive filling of anti-bonding molecular orbitals. A comparison between the closely related [C_4_O_4_]^4–^ and [C_4_O_4_]^6–^ fragments in [**2**-K(C_7_H_8_)]_2_ and [**4**-K_2_(BEt_3_)]_2_, respectively, reflects the net conversion of C–O bonds to C–C bonds in the presence of an additional reductant. The roles of the [Et_3_BH]^−^ reagent in bringing about this transformation (acting as a source of both electrons and of the BEt_3_ fragment bound at C–O^–^) suggest that it acts as a surrogate for H_2_ in the Fischer–Tropsch process.^2^

## Conclusions

We have shown here that four- or six-electron reduction of CO can be accomplished by the use of anionic aluminum(I) (“aluminyl”) compounds of the type M_*n*_[(NON)Al]_*n*_ to give topologically linear or branched C_4_/C_6_ chains depending on the reaction conditions. The mechanism for homologation to the [C_4_O_4_]^4–^ system proceeds via the rate-limiting formation of the central C=C double bond from two carbene fragments and rationalizes the synthesis of this system from both monomeric (M = Li) and dimeric (M = K) precursors. Initial adduct formation with CO relies on the highly electron-rich nature of the aluminyl reagent, which drives an unusual (primarily Z-type) mode of interaction of the CO molecule with the metal center. The onward formation of [C_6_O_6_]^4–^ from [C_4_O_4_]^4–^ demonstrates for the first time a homogeneous process which brings about chain branching via complete C–O bond cleavage. A comparison of the linear [C_4_O_4_]^4–^ system with the [C_4_O_4_]^6–^ congener formed under more reducing conditions reflects the net conversion of C–O bonds to C–C bonds in the presence of an additional reductant.

## Experimental Section

Complete details of the synthetic methods and characterizing data, crystallographic information, and details of quantum chemical studies are provided in the Supporting Information. Starting materials **NON**AlI,^51a^ K_2_[**NON**Al]_2_,^51a^ [Li(OEt_2_)_2_] [**NON**Al],^57^ and Ag_2_(squarate)^65^ were prepared according to literature methods. All other reagents were used as received.

### [K(C_7_H_8_)]_2_[{(NON)Al}_2_(C_4_O_4_)], [**2**-K(C_7_H_8_)]_2_

To a 25 mL reaction bomb were added [**1**-K]_2_ (250 mg, 0.34 mmol) and benzene (7.5 mL). The resulting mixture was degassed twice using the freeze–pump–thaw method and sealed under vacuum. The bomb was then warmed to 307 K in an oil bath, and after a few minutes, the headspace was charged with CO (1.5 atm, ca. 1.2 mmol). After sealing the vessel, the mixture was vigorously shaken for 30 s and subsequently allowed to stand undisturbed at 307 K for 16 h. Recrystallization from toluene led to the formation of small orange crystals of [**2**-K(C_7_H_8_)]_2_·4(C_7_H_8_); isolated yield of single crystals: 37 mg, 11%. Anal. Calcd for C_98_H_124_Al_2_K_2_N_4_O_6_ + 1.5 toluene: C, 75.57; H, 7.95; N, 3.25. Found: C, 75.52; H, 8.04; N, 3.22. ^1^H NMR (400 MHz, THF-*d*_8_): δ 7.07–6.95 (m, 6H, Dipp-Ar-C*H*), 6.42 (d, *J* = 1.9 Hz, 2H, XA-C*H*^1^), 5.62 (d, *J* = 1.9 Hz, 2H, XA-C*H*^3^), 3.30–3.14 (m, 4H, C*H*Me_2_), 1.73 (s, 3H, C(C*H*_3_)_2_), 1.56 (s, 3H, C(CH_3_)_2_), 1.11 (d, *J* = 6.8 Hz, 6H, CH(C*H*_3_)_2_), 1.04 (s, 18H, C(C*H*_3_)_3_), 0.86 (d, *J* = 6.8 Hz, 6H, CH(C*H*_3_)_2_), 0.68 (d, *J* = 6.8 Hz, 6H, CH(C*H*_3_)_2_), 0.43 (d, *J* = 6.8 Hz, 6H, CH(C*H*_3_)_2_). ^13^C NMR (126 MHz, THF-*d*_8_): δ 148.2 (Dipp-*o*-*C*), 147.8 (*C*^t^Bu), 147.6 (Dipp-*o*-*C*), 146.8 (XA-*C*N), 144.8 (Dipp-*i*-*C*), 142.5 (XA-*C*O), 133.8 (*C*CMe_2_), 131.3 (C(=O)*C*O), 129.2 (C_6_H_6_), 125.4 (Dipp-Ar-*C*H), 125.2 (Dipp-Ar-*C*H), 123.6 (Dipp-*m*-*C*H), 111.0 (XA-*C*^3^H), 105.3 (XA-*C*^1^H), 37.9 (*C*CMe_2_), 35.5 (*C*Me_3_), 32.6 (C(*C*H_3_)_2_), 32.2 (C(*C*H_3_)_3_), 29.1 (*C*H(CH_3_)_2_), 28.5 (*C*H(CH_3_)_2_), 26.3 (CH(*C*H_3_)_2_), 25.5* (CH(*C*H_3_)_2_), 24.7 (CH(*C*H_3_)_2_), 24.5 (CH(*C*H_3_)_2_), 23.8 (C(*C*H_3_)_2_). *Overlapped with solvent signal—located in HSQC and HMBC. Due to quadrupolar broadening, the resonance associated with the Al–*C*(=O) group was not observed in the range −30 to 300 ppm. The corresponding signal is measured for the related derivative [**2**-K(12-crown-4)]_2_ at 296.2 ppm.

### [K(12-c-4)]_2_[{(NON)Al}_2_(C_4_O_4_)], [**2**-K(12-c-4)]_2_

(Method 1): To a suspension of [**2**-K(C_7_H_8_)]_2_ (15 mg, 0.009 mmol) in benzene-*d*_6_ (0.5 ml) was added a solution of 12-crown-4 in toluene (0.18 mL, 0.1 M, 0.018 mmol, 2.05 equiv), and the reaction mixture was heated at 358 K for 2 h. Volatiles were removed under vacuum, and the product was obtained as an orange powder. Yield 13 mg, 80% (quantitative by NMR). (Method 2): To a solution of [**1**-K]_2_ (0.120 g, 0.163 mmol) in toluene (5 mL) was added a solution of 12-crown-4 in toluene (3.2 mL of a 0.1 M solution, 0.320 mmol). The resulting red solution was stirred at room temperature for 5 min before addition of Fe(CO)_5_ (22 μL, 0.161 mmol). The resulting dark red solution was stirred overnight. Removal of volatiles yields a yellow oil, which can be dissolved in minimal benzene and stored at room temperature overnight to yield single crystals of [**2**-K(12-c-4)]_2_·4(C_6_H_6_) suitable for X-ray crystallography. Isolated yield of single crystals ca. 5 mg, 20% (30% by NMR). ^1^H NMR (600 MHz, THF-*d*_8_): δ 7.01 (t, *J* = 7.0 Hz, 2H, Dipp-*p*-C*H*), 6.96 (d, *J* = 7.0 Hz, 4H, Dipp-*m*-C*H*), 6.40 (d, *J* = 2.0 Hz, 2H, XA-C*H*^1^), 5.59 (d, *J* = 1.9 Hz, 2H, XA-C*H*^3^), 3.60 (s, 16H, 12-crown-4), 3.42–3.34 (m, 2H, C*H*Me_2_), 3.14 (sept., *J* = 6.9 Hz, 2H, C*H*Me_2_), 1.73 (s, 3H, C(C*H*_3_)_2_), 1.58 (s, 3H, C(C*H*_3_)_2_), 1.24 (d, *J* = 6.7 Hz, 6H, CH(C*H*_3_)_2_), 1.03 (s, 18H, C(C*H*_3_)_3_), 0.79 (d, *J* = 6.8 Hz, 6H, CH(C*H*_3_)_2_), 0.77 (d, *J* = 6.8 Hz, 6H, CH(C*H*_3_)_2_), 0.33 (d, *J* = 6.8 Hz, 6H, CH(C*H*_3_)_2_). ^13^C NMR (151 MHz, THF-*d*_8_): δ 296.2 (Al*C*O), 147.7 (Dipp-*o*-*C*), 147.5 (Dipp-*o*-*C*), 147.4 (*C*^t^Bu), 146.9 (XA-*C*N), 145.2 (Dipp-*i*-*C*), 142.7 (XA-*C*O), 134.1 (*C*CMe_2_), 130.8 (C(=O)*C*O), 125.9 (Dipp-*m*-*C*H), 125.1 (Dipp-*p*-*C*H), 123.1 (Dipp-*m*-*C*H), 111.4 (XA-*C*^3^H), 105.3 (XA-*C*^1^H), 70.5 (O*C*H_2_), 37.8 (*C*Me_2_), 35.4 (*C*(CH_3_)_3_), 32.2 (C(*C*H_3_)_3_), 32.0 (C(*C*H_3_)_2_), 29.4 (*C*HMe_2_), 28.7 (*C*HMe_2_), 26.5 (CH(*C*H_3_)_2_), 25.0 (CH(*C*H_3_)_2_), 24.6 (CH(*C*H_3_)_2_), 24.3 (CH(*C*H_3_)_2_), 23.6 (C(*C*H_3_)_2_).

### [Li(OEt_2_)][{(NON)Al}_2_(C_4_O_4_)], [**2**-Li(OEt_2_)]_2_ and [Li(THF)]_2_[{(NON)Al}_2_(C_4_O_4_)], [**2**-Li(THF)]_2_

#### Ether Adduct

(Method 1) A solution of [**1**-Li] (15 mg, 0.017 mmol) in C_6_D_6_ (0.5 mL) was degassed twice using the freeze–pump–thaw method. The headspace was then charged with CO (2 bar) and briefly shaken. ^1^H NMR reveals the formation of a mixture of products, including [**2**-Li(OEt_2_)]_2_ in ca. 40% yield. Upon concentration (to ca. 1/3 volume) and standing for 2 days, X-ray quality crystals of [**2**-Li(OEt_2_)]_2_ formed.

(Method 2) [**2**-K(C_7_H_8_)]_2_ (30 mg, 0.018 mmol) and LiI (5 mg, 2.1 equiv) were dissolved in Et_2_O (15 mL) and briefly heated to 318 K in a sealed ampoule, leading to a color change from orange to yellow. The mixture was allowed to cool to room temperature, and the resulting cloudy mixture was filtered onto benzene (8 mL). After mixing, the solution was concentrated until it turned turbid (ca. 5 mL). At this point, a sample (0.2 mL) was taken and diluted with C_6_D_6_ (0.3 mL), and a ^1^H NMR spectrum was measured. The resulting spectrum shows the presence of a single species, which has the same spectroscopic signals as those obtained using method 1 in a J. Young’s NMR tube. ^1^H NMR (400 MHz, C_6_D_6_): δ 6.67 (d, *J* = 2.0 Hz, 2H, XA-C*H*^1^), 6.00 (d, *J* = 2.0 Hz, 2H, XA-C*H*^3^), 3.72 (sept, *J* = 6.9 Hz, 2H), 1.70 (s, 3H, C(C*H*_3_)_2_), 1.61 (s, 3H, C(C*H*_3_)_2_), 1.52 (d, *J* = 6.9 Hz, 6H, CH(C*H*_3_)_2_), 1.19 (s, 18H, C(C*H*_3_)_3_), 0.36 (d, *J* = 6.9 Hz, 6H, CH(C*H*_3_)_2_). Samples of [**2**-Li(OEt_2_)]_2_ lose Et_2_O under continuous vacuum. Dissolution in THF-*d*_8_, however, yields spectroscopic signals indistinguishable from samples of [**2**-Li(THF)]_2_ prepared as set out below.

#### THF Adduct

A solution containing [**2**-K(C_7_H_8_)]_2_ (ca. 20 mg, 0.012 mmol) was prepared as described above, and Li[HB^s^Bu_3_] (1 mL of a 1 M solution in THF) was added. The mixture was heated at 307 K for 16 h, affording small yellow crystals. Analytically pure samples can be obtained by recrystallization from minimal benzene or THF and washing with THF. Isolated yield of single crystals: 7 mg, 30% over last step. Anal. Calcd for C_106_H_140_Al_2_Li_2_N_4_O_8_ + 1.75 THF: C, 75.73; H, 8.66; N, 3.13. Found: C, 75.13; H, 8.66; N, 3.21. ^1^H NMR (400 MHz, C_6_D_6_): δ 7.25 (m, 6H, Dipp-Ar-C*H*), 6.70 (d, *J* = 2.0 Hz, 2H, XA-C*H*^1^), 6.05 (d, *J* = 2.0 Hz, 2H, XA-C*H*^3^), 3.77 (sept, *J* = 6.9 Hz, 2H, C*H*Me_2_), 3.70 (s, m, 4H, THF), 3.41 (sept, *J* = 6.9 Hz, 2H, C*H*Me_2_), 1.69 (s, 3H, C(C*H*_3_)_2_), 1.60 (s, 3H, C(C*H*_3_)_2_), 1.58 (d, *J* = 6.9 Hz, 6H, CH(C*H*_3_)_2_), 1.32 (m, 4H, THF), 1.21 (s, 18H, C(C*H*_3_)_3_), 1.15 (overlapping d, *J* = 6.9 Hz, 12H, CH(C*H*_3_)_2_), 0.36 (d, *J* = 6.9 Hz, 6H, CH(C*H*_3_)_2_). ^13^C NMR (151 MHz, C_6_D_6_): δ 147.8 (*C*^t^Bu), 147.2 (Dipp-*o*-*C*), 146.3 (Dipp-*o*-*C*), 145.7 (XA-*C*^4^), 143.6 (Dipp-*i*-*C*), 141.4 (XA-*C*O), 133.1 (*C*CMe_2_), 125.8 (Dipp-*p*-*C* and Dipp-*m*-*C*), 123.0 (Dipp-*m*-*C*), 111.3 (XA-*C*^3^H), 106.4 (XA-*C*^1^H), 68.6 (THF-*C*O), 37.0 (*C*Me_2_), 35.0 (*C*Me_3_), 32.1 (C(*C*H_3_)_2_), 31.9 (C(*C*H_3_)_3_), 29.5 (*C*HMe_2_), 28.2 (*C*HMe_2_), 26.3 (CH(*C*H_3_)_2_), 25.4 (THF), 24.1 (CH(*C*H_3_)_2_), 23.9 (CH(*C*H_3_)_2_), 23.2 (C(*C*H_3_)_2_), 23.2 (CH(*C*H_3_)_2_). Due to quadrupolar broadening (and overlap with the solvent), the resonances associated with the [C_4_O_4_]^4–^ fragment were not observed in the range −30 to 300 ppm. The corresponding signals are measured for the related derivative [**2**-K(12-crown-4)]_2_ at 130.8 and 296.2 ppm. ^7^Li NMR (156 MHz, C_6_D_6_) δ 0.61.

### K[K(THF)_2_][{(NON)Al}_2_(C_6_O_6_)], **3**

[**2**-K(C_7_H_8_)]_2_ (25 mg, 0.015 mmol) was suspended in THF-*d*_8_ (0.5 mL) in a J. Young’s NMR tube and degassed twice using the freeze–pump–thaw method. The headspace was then charged with CO (2 atm) and heated at 339 K for 4 d. During this time, [**2**-K(C_7_H_8_)]_2_ dissolved, and a color change from yellow to red to orange was observed. ^1^H NMR monitoring shows quantitative conversion to a single product. After concentrating the solution to a quarter of its original, small pale orange plates formed at the surface of the solution. Anal. Calcd for C_108_H_140_Al_2_K_2_N_4_O_10_ + 2 THF: C, 72.16; H, 8.14; N, 2.90. Found: C, 72.13; H, 8.06; N, 2.55. ^1^H NMR (600 MHz, THF-*d*_8_): δ 7.43 (t, *J* = 7.6 Hz, 2H, Dipp-*p*-C*H*), 7.32 (t, *J* = 7.6 Hz, 2H, Dipp-*p*-C*H*), 7.25 (dd, *J* = 7.8, 1.6 Hz, 2H, Dipp-*m*-C*H*), 7.16 (dd, *J* = 7.6, 1.6 Hz, 2H, Dipp-*m*-C*H*), 7.09 (dd, *J* = 7.8, 1.6 Hz, 2H, Dipp-*m*-C*H*), 7.00 (dd, *J* = 7.7, 1.6 Hz, 2H, Dipp-*m*-C*H*), 6.49 (d, *J* = 1.9 Hz, 2H, XA-C^1^*H*), 6.42 (d, *J* = 1.9 Hz, 2H, XA-C^1^*H*), 5.36 (d, *J* = 1.9 Hz, 2H, XA-C^3^*H*), 5.34 (d, *J* = 1.9 Hz, 2H, XA-C^3^*H*), 3.46–3.36 (overlapping sept., 4H, C*H*Me_2_), 3.32 (sept., *J* = 7.2 Hz, 2H, C*H*Me_2_), 3.16 (sept., *J* = 6.9 Hz, 2H, C*H*Me_2_), 1.75 (s, 3H, C(C*H*_3_)_2_), 1.73 (s, 3H, C(C*H*_3_)_2_), 1.57 (s, 3H, C(C*H*_3_)_2_), 1.54 (s, 3H, C(C*H*_3_)_2_), 1.20 (d, *J* = 7.0 Hz, 6H, CH(C*H*_3_)_2_), 1.03 (s, 18H, C(C*H*_3_)_3_), 1.00 (s, 18H, C(C*H*_3_)_3_), 0.95 (d, *J* = 6.9 Hz, 6H, CH(C*H*_3_)_2_), 0.87 (d, *J* = 6.7 Hz, 6H, CH(C*H*_3_)_2_), 0.83 (d, *J* = 6.8 Hz, 6H, CH(C*H*_3_)_2_), 0.80 (d, *J* = 6.7 Hz, 6H, CH(C*H*_3_)_2_), 0.73 (d, *J* = 6.7 Hz, 6H, CH(C*H*_3_)_2_), 0.68 (d, *J* = 6.8 Hz, 6H, CH(C*H*_3_)_2_), 0.51 (d, *J* = 6.8 Hz, 6H, CH(C*H*_3_)_2_). ^13^C NMR (151 MHz, THF-*d*_8_): δ 258.6 (C_6_O_6_), 173.5 (C_6_O_6_), 169.0 (C_6_O_6_), 156.2 (C_6_O_6_), 149.4 (Dipp-*o*-*C*), 149.1 (Dipp-*o*-*C*), 148.0 (Dipp-*o*-*C*), 147.6 (*C*^t^Bu), 147.5 (Dipp-*o*-*C*), 147.3 (*C*^t^Bu), 146.1 (XA-*C*^4^), 145.8 (Dipp-*i*-*C*), 145.7 (Dipp-*i*-*C*), 145.6 (XA-*C*^4^), 142.7 (XA-*C*O), 141.5 (XA-*C*O), 133.7 (*C*CMe_2_), 133.3 (*C*CMe_2_), 129.2 (free C_6_H_6_), 127.4 (Dipp-*p*-*C*H), 127.3 (Dipp-*p*-*C*H), 127.2 (Dipp-*m*-*C*H), 126.2 (Dipp-*m*-*C*H), 124.4 (Dipp-*m*-*C*H), 123.8 (Dipp-*m*-*C*H), 118.2 (C_6_O_6_), 111.3 (XA-*C*^3^H), 110.5 (C_6_O_6_), 106.5 (XA-*C*^1^H), 105.9 (XA-*C*^1^H), 37.9 (*C*Me_2_), 37.8 (*C*Me_2_), 35.5 (*C*Me_3_), 35.4 (*C*Me_3_), 32.2 (C(*C*H_3_)_3_), 32.1 (C(*C*H_3_)_3_), 32.1 (C(*C*H_3_)_2_), 32.0 (C(*C*H_3_)_2_), 29.2 (*C*HMe_2_), 28.8 (*C*HMe_2_), 28.1 (*C*HMe_2_), 28.0 (*C*HMe_2_), 26.2 (CH(*C*H_3_)_2_), 26.1 (CH(*C*H_3_)_2_), 26.0 (CH(*C*H_3_)_2_), 25.3 (CH(*C*H_3_)_2_), 25.2 (CH(*C*H_3_)_2_), 24.9 (CH(*C*H_3_)_2_), 22.9 (C(*C*H_3_)_2_), 22.9 (C(*C*H_3_)_2_).

### K_4_[{(NON)Al}_2_(C_4_O_4_) (BEt_3_)_2_], [**4**-K_2_(BEt_3_)]_2_

To a stirred solution containing [**1**-K]_2_ (220 mg, 0.30 mmol), benzene (6.6 mL), and CO (ca. 1.5 bar) in a 25 mL reaction bomb was added K[HBEt_3_] (1.5 mL of a 1 M solution in THF, 1.5 mmol). After 24 h, the solution was concentrated to a fifth of its original volume under reduced pressure. Prolonged standing led to the formation of colorless extremely sensitive crystals of [**4**-K_2_(BEt_3_)]_2_. Yield 12 mg, 4%. ^1^H NMR (400 MHz, THF-*d*_8_): δ 7.27 (br s, 6H, Dipp-Ar-C*H*), 6.36 (br s, 2H, XA-C*H*^1^), 5.28 (s, 2H, XA-C*H*^3^), 3.79 (br s, 2H, C*H*Me_2_), 3.47 (sept, *J* = 6.6 Hz, 2H, C*H*Me_2_), 1.68 (s, 6H, C(C*H*_3_)_2_), 1.28 (br s, 6H, CH(C*H*_3_)_2_), 1.00 (s, 18H, C(C*H*_3_)_3_), 0.80 (d, *J* = 6.6 Hz, 13H), 0.57 (t, *J* = 7.7 Hz, 9H, BCH_2_C*H*_3_), 0.06 (q, *J* = 7.7 Hz, 6H, BC*H*_2_CH_3_). Due to the low solubility and stability of this compound in solution, no satisfactory ^11^B and ^13^C spectra could be obtained.

### [(NON)Al]_2_(C_4_O_4_), **5**

(NON)AlI (500 mg, 0.60 mmol, 2.0 equiv) and Ag_2_(C_4_O_4_) (110 mg, 0.33 mmol) were suspended in Et_2_O (10 mL), and the reaction mixture was refluxed at 308 K for 48 h in the dark. Volatiles were removed in vacuo, and the residue was extracted into toluene (15 mL), filtered, and layered with hexane (15 mL). Upon standing for several days, crystals of **5** suitable for single-crystal X-ray diffraction were obtained. Yield 180 mg, 39%. Anal. Calcd for C_98_H_124_Al_2_N_4_O_6_ + 0.5 hexane: C, 78.21; H, 8.51; N, 3.61. Found 78.08; H, 8.76; N, 3.34. ^1^H NMR (400 MHz, C_6_D_6_): δ 7.20 (m, 2H, Dipp-*p*-C*H*), 7.14 (m, 4H, Dipp-*p*-C*H*), 6.72 (d, *J* = 1.9 Hz, 2H, XA-C*H*^1^), 5.98 (d, *J* = 1.9 Hz, 2H, XA-C*H*^3^), 3.54 (sept, *J* = 6.8 Hz, 4H, C*H*Me_2_), 1.58 (s, 6H, C(C*H*_3_)_2_), 1.15 (s, 18H, C(C*H*_3_)_3_), 1.06 (d, *J* = 6.8 Hz, 12H, CH(C*H*_3_)_2_), 0.91–0.83 (br s, 12H, CH(C*H*_3_)_2_). ^13^C NMR (126 MHz, C_6_D_6_): δ 194.0 (*C*_4_O_4_), 148.9 (*C*^t^Bu), 147.0 (Dipp-*o*-*C*), 144.4 (XA-*C*N), 141.0, 140.8 (Dipp-*i*-*C*, XA-*C*O), 133.3 (*C*CMe_2_), 127.0 (Dipp-*p*-*C*), 125.1 (Dipp-*m*-*C*), 112.1 (XA-*C*^3^H), 108.2 (XA-*C*^1^H), 37.2 (*C*Me_2_), 35.1(*C*Me_3_), 31.7 (C(*C*H_3_)_3_), 28.4 (*C*HMe_2_), 26.4 (br, C(*C*H_3_)_2_), 25.4 (CH(*C*H_3_)_2_), 24.7(CH(*C*H_3_)_2_).

## Supporting Information Available

Complete details of synthetic procedures and characterizing data; representative spectra; details of quantum chemical calculations; and *xyz* coordinates for optimized structures (PDF)

## References

[ref1] Schulz H. Short history and present trends of Fischer–Tropsch synthesis. Appl. Catal., A 1999, 186, 3–12. 10.1016/S0926-860X(99)00160-X.

[ref2] van Santen R. A.; Ciobîcă I. M.; van Steen E.; Ghouri M. M. Mechanistic Issues in Fischer–Tropsch Catalysis, 1st ed.; Elsevier Inc.: Amsterdam, 2011; Vol. 54, pp 127–187.

[ref3] Kaneko T.; Derbyshire F.; Makino E.; Gray D.; Tamura M.; Li K. Coal Liquefaction. Ullmann’s Encyclopedia of Industrial Chemistry; Wiley-VCH: Weinheim, 2012.

[ref4] For a recent review, see Kong R. Y.; Crimmin M. R. Cooperative strategies for CO homologation. Dalton Trans. 2020, 49, 16587–16597. 10.1039/D0DT01564D. 32530017

[ref5] Manriquez J. M.; McAlister D. R.; Sanner R. D.; Bercaw J. E. Reduction of carbon monoxide promoted by alkyl and hydride derivatives of permethylzirconocene. J. Am. Chem. Soc. 1978, 100, 2716–2724. 10.1021/ja00477a025.

[ref6] Bianconi P. A.; Williams I. D.; Engeler M. P.; Lippard S. J. Reductive coupling of two carbon monoxide ligands to form a coordinated alkyne. J. Am. Chem. Soc. 1986, 108, 311–313. 10.1021/ja00262a030.

[ref7] Bianconi P. A.; Vrtis R. N.; Rao C. P.; Williams I. D.; Engeler M. P.; Lippard S. J. Reductive coupling of carbon monoxide ligands to form coordinated bis(trimethylsiloxy)ethyne in seven-coordinate niobium(I) and tantalum(I) [M(CO)_2_(dmpe)_2_Cl] complexes. Organometallics 1987, 6, 1968–1977. 10.1021/om00152a023.

[ref8] Coffin V. L.; Brennen W.; Wayland B. B. Thermodynamic studies of competitive adduct formation: single- and double-insertion reactions of carbon monoxide with rhodium octaethylporphyrin dimer. J. Am. Chem. Soc. 1988, 110, 6063–6069. 10.1021/ja00226a022. 22148782

[ref9] Vrtis R. N.; Rao C. P.; Bott S. G.; Lippard S. J. Synthesis and stabilization of tantalum-coordinated dihydroxyacetylene from two reductively coupled carbon monoxide ligands. J. Am. Chem. Soc. 1988, 110, 7564–7566. 10.1021/ja00230a062.

[ref10] Wayland B. B.; Sherry A. E.; Coffin V. L. Selective reductive coupling of carbon monoxide. J. Chem. Soc., Chem. Commun. 1989, 662–663. 10.1039/C39890000662.

[ref11] Protasiewicz J. D.; Lippard S. J. Vanadium-promoted reductive coupling of carbon monoxide and facile hydrogenation to form cis-disiloxyethylenes. J. Am. Chem. Soc. 1991, 113, 6564–6570. 10.1021/ja00017a030.

[ref12] Cummins C. C.; Van Duyne G. D.; Schaller C. P.; Wolczanski P. T. Carbonylation of zirconium complex [tert-Bu_3_SiNH]_3_ZrH and x-ray structure study of [tert-Bu_3_SiNH]_3_ZrCH_3_. Organometallics 1991, 10, 164–170. 10.1021/om00047a044.

[ref13] Miller A. J. M.; Labinger J. A.; Bercaw J. E. Reductive coupling of carbon monoxide in a rhenium carbonyl complex with pendant Lewis acids. J. Am. Chem. Soc. 2008, 130, 11874–11875. 10.1021/ja805108z. 18702489

[ref14] Watanabe T.; Ishida Y.; Matsuo T.; Kawaguchi H. Reductive coupling of six carbon monoxides by a ditantalum hydride complex. J. Am. Chem. Soc. 2009, 131, 3474–3475. 10.1021/ja9007276. 19243097

[ref15] Buss J. A.; Agapie T. Four-electron deoxygenative reductive coupling of carbon monoxide at a single metal site. Nature 2016, 529, 72–75. 10.1038/nature16154. 26689364

[ref16] Sharpe H. R.; Geer A. M.; Taylor L. J.; Gridley B. M.; Blundell T. J.; Blake A. J.; Davies E. S.; Lewis W.; McMaster J.; Robinson D.; Kays D. L. Selective reduction and homologation of carbon monoxide by organometallic iron complexes. Nat. Commun. 2018, 9, 3757 10.1038/s41467-018-06242-w. 30217985 PMC6138626

[ref17] aKong R. Y.; Crimmin M. R. Carbon Chain Growth by Sequential Reactions of CO and CO_2_ with [W(CO)_6_] and an aluminum(I) Reductant. J. Am. Chem. Soc. 2018, 140, 13614–13617. 10.1021/jacs.8b09761. 30351139

[ref18] Lalrempuia R.; Kefalidis C. E.; Bonyhady S. J.; Schwarze B.; Maron L.; Stasch A.; Jones C. Activation of CO by hydrogenated magnesium(I) dimers: sterically controlled formation of ethenediolate and cyclopropanetriolate complexes. J. Am. Chem. Soc. 2015, 137, 8944–8947. 10.1021/jacs.5b06439. 26135846

[ref19] Anker M. D.; Hill M. S.; Lowe J. P.; Mahon M. F. Alkaline-earth-promoted CO homologation and reductive catalysis. Angew. Chem., Int. Ed. 2015, 54, 10009–10011. 10.1002/anie.201505851. PMC4678424 26220407

[ref20] Yuvaraj K.; Douair I.; Paparo A.; Maron L.; Jones C. Reductive trimerization of CO to the deltate dianion using activated magnesium(I) compounds. J. Am. Chem. Soc. 2019, 141, 8764–8768. 10.1021/jacs.9b04085. 31096751

[ref21] Yuvaraj K.; Douair I.; Jones D. D. L.; Maron L.; Jones C. Sterically controlled reductive oligomerisations of CO by activated magnesium(I) compounds: deltate vs. ethenediolate formation. Chem. Sci. 2020, 11, 3516–3522. 10.1039/D0SC00836B. 34109023 PMC8152598

[ref22] Paparo A.; Yuvaraj K.; Matthews A. J. R.; Douair I.; Maron L.; Jones C. Reductive hexamerization of CO involving cooperativity between magnesium(I) reductants and [Mo(CO)_6_]: synthesis of well-defined magnesium benzenehexolate complexes. Angew. Chem., Int. Ed. 2021, 60, 630–634. 10.1002/anie.202009523. 32969564

[ref23] Liu H.-Y.; Schwamm R. J.; Neale S. E.; Hill M. S.; McMullin C. L.; Mahon M. F. Reductive dimerization of CO by a Na/Mg(I) Diamide. J. Am. Chem. Soc. 2021, 143, 17851–17856. 10.1021/jacs.1c09467. 34652134 PMC8554760

[ref24] Wang X.; Zhu Z.; Peng Y.; Lei H.; Fettinger J. C.; Power P. P. Room-temperature reaction of carbon monoxide with a stable diarylgermylene. J. Am. Chem. Soc. 2009, 131, 6912–6913. 10.1021/ja9017286. 19413315

[ref25] Braunschweig H.; Dellermann T.; Dewhurst R. D.; Ewing W. C.; Hammond K.; Jimenez-Halla J. O. C.; Kramer T.; Krummenacher I.; Mies J.; Phukan A. K.; Vargas A. Metal-free binding and coupling of carbon monoxide at a boron–boron triple bond. Nat. Commun. 2013, 5, 1025–1028. 10.1038/nchem.1778. 24256866

[ref26] Majumdar M.; Omlor I.; Yildiz C. B.; Azizoglu A.; Huch V.; Scheschkewitz D. Reductive cleavage of carbon monoxide by a disilenide. Angew. Chem., Int. Ed. 2015, 54, 8746–8750. 10.1002/anie.201503455. 26088688

[ref27] Anker M. D.; Kefalidis C. E.; Yang Y.; Fang J.; Hill M. S.; Mahon M. F.; Maron L. Alkaline earth-centered CO homologation, reduction, and amine carbonylation. J. Am. Chem. Soc. 2017, 139, 10036–10054. 10.1021/jacs.7b04926. 28640639

[ref28] Shi X.; Hou C.; Zhou C.; Song Y.; Cheng J. A molecular barium hydrido complex stabilized by a super-bulky hydrotris(pyrazolyl)borate ligand. Angew. Chem., Int. Ed. 2017, 56, 16650–16653. 10.1002/anie.201709344. 29125204

[ref29] Protchenko A. V.; Vasko P.; Do D. C. H.; Hicks J.; Fuentes M. Á.; Jones C.; Aldridge S. Reduction of carbon oxides by an acyclic silylene: reductive coupling of CO. Angew. Chem., Int. Ed. 2019, 58, 1808–1812. 10.1002/anie.201812675. 30537262

[ref30] Wang Y.; Kostenko A.; Hadlington T. J.; Luecke M.-P.; Yao S.; Driess M. Silicon-mediated selective homo- and heterocoupling of carbon monoxide. J. Am. Chem. Soc. 2019, 141, 626–634. 10.1021/jacs.8b11899. 30516372

[ref31] Xu M.; Qu Z.-W.; Grimme S.; Stephan D. W. Lithium Dicyclohexylamide in Transition-Metal-Free Fischer-Tropsch Chemistry. J. Am. Chem. Soc. 2021, 143, 634–638. 10.1021/jacs.0c11482. 33399459

[ref32] Yuvaraj K.; Jones C. Reductive coupling of CO with magnesium anthracene complexes: formation of magnesium enediolates. Chem. Commun. 2021, 57, 9224–9227. 10.1039/D1CC03890G. 34519307

[ref33] Evans M. J.; Gardiner M. G.; Anker M. D.; Coles M. P. Extending chain growth beyond C_1_ → C_4_ in CO homologation: aluminyl promoted formation of the [C_5_O_5_]^5–^ ligand. Chem. Commun. 2022, 58, 5833 10.1039/D2CC01554D. 35452064

[ref34] Fagan P. J.; Manriquez J. M.; Marks T. J.; Day V. W.; Vollmer S. H.; Day C. S. Carbon monoxide activation by f-element organometallics. An unusually distorted, carbene-like dihaptoacyl and CO tetramerization. J. Am. Chem. Soc. 1980, 102, 5393–5396. 10.1021/ja00536a048.

[ref35] Fagan P. J.; Moloy K. G.; Marks T. J. Carbon monoxide activation by organoactinides. Migratory carbon monoxide insertion into metal-hydrogen bonds to produce polynuclear formyls. J. Am. Chem. Soc. 1981, 103, 6959–6962. 10.1021/ja00413a032.

[ref36] Evans W. J.; Wayda A. L.; Hunter W. E.; Atwood J. L. Organolanthanoid activation of carbon monoxide: single and multiple insertion of CO into t-butyl lanthanoid bonds; X-ray crystallographic identification of a new bonding mode for a bridging enedione diolate ligand formed by formal coupling of four CO molecules. J. Chem. Soc., Chem. Commun. 1981, 706–708. 10.1039/C39810000706.

[ref37] Evans W. J.; Grate J. W.; Hughes L. A.; Zhang H.; Atwood J. L. Reductive homologation of CO to a ketenecarboxylate by a low-valent organolanthanide complex—synthesis and X-ray crystal-structure of [(C_5_Me_5_)_4_Sm_2_(O_2_CCCO)(THF)]_2_. J. Am. Chem. Soc. 1985, 107, 3728–3730. 10.1021/ja00298a060.

[ref38] Radu N. S.; Engeler M. P.; Gerlach C. P.; Tilley T. D.; Rheingold A. L. Isolation of the first d^0^ metalloxy ketene complexes via “double insertion” of carbon monoxide into thorium-silicon bonds. J. Am. Chem. Soc. 1995, 117, 3621–3622. 10.1021/ja00117a035.

[ref39] Ferrence G. M.; McDonald R.; Takats J. Stabilization of a discrete lanthanide(II) hydrido complex by a bulky hydridotris(pyrazolyl)borate ligand. Angew. Chem., Int. Ed. 1999, 38, 2233–2237. 10.1002/(sici)1521-3773(19990802)38:15<2233::aid-anie2233>3.0.co;2-x. 10425494

[ref40] Summerscales O. T.; Cloke F. G. N.; Hitchcock P. B.; Green J. C.; Hazari N. Reductive cyclotrimerization of carbon monoxide to the deltate dianion by an organometallic uranium complex. Science 2006, 311, 829–831. 10.1126/science.1121784. 16469921

[ref41] Summerscales O. T.; Cloke F. G. N.; Hitchcock P. B.; Green J. C.; Hazari N. Reductive cyclotetramerization of CO to squarate by a U(III) complex: the X-ray crystal structure of [(U(η-C_8_H_6_{(SiPr_3_)-Pr-i-1,4}_2_)(η-C_5_Me_4_H)]_2_(μ–η^2^:η^2^-C_4_O_4_). J. Am. Chem. Soc. 2006, 128, 9602–9603. 10.1021/ja063222r. 16866493

[ref42] Evans W. J.; Lee D. S.; Ziller J. W.; Kaltsoyannis N. Trivalent [(C_5_Me_5_)_2_(THF)Ln]_2_(μ–η^2^:η^2^-N_2_) complexes as reducing agents including the reductive homologation of CO to a ketene carboxylate, (μ–η^4^-O_2_C–C=C=O)^2–^. J. Am. Chem. Soc. 2006, 128, 14176–14184. 10.1021/ja0640851. 17061902

[ref43] Werkema E. L.; Maron L.; Eisenstein O.; Andersen R. A. Reactions of Monomeric [1,2,4-(Me_3_C)_3_C_5_H_2_]_2_CeH and CO with or without H_2_: An Experimental and Computational Study. J. Am. Chem. Soc. 2007, 129, 2529–2541. 10.1021/ja066482h. 17286402

[ref44] Frey A. S.; Cloke F. G. N.; Hitchcock P. B.; Day I. J.; Green J. C.; Aitken G. Mechanistic studies on the reductive cyclooligomerisation of CO by U(III) mixed sandwich complexes; the molecular structure of [U(η-C_8_H_6_{Si^i^Pr_3_-1,4}_2_)(η-Cp*)]_2_(μ-η^1^:η^1^-C_2_O_2_). J. Am. Chem. Soc. 2008, 130, 13816–13817. 10.1021/ja8059792. 18817397

[ref45] Arnold P. L.; Turner Z. R.; Bellabarba R. M.; Tooze R. P. Carbon monoxide coupling and functionalisation at a simple uranium coordination complex. Chem. Sci. 2011, 2, 77–79. 10.1039/C0SC00452A.

[ref46] Mansell S. M.; Kaltsoyannis N.; Arnold P. L. Small molecule activation by uranium tris(aryloxides): experimental and computational studies of binding of N_2_, coupling of CO, and deoxygenation insertion of CO_2_ under ambient conditions. J. Am. Chem. Soc. 2011, 133, 9036–9051. 10.1021/ja2019492. 21591662

[ref47] Gardner B. M.; Stewart J. C.; Davis A. L.; McMaster J.; Lewis W.; Blake A. J.; Liddle S. T. Homologation and functionalization of carbon monoxide by a recyclable uranium complex. Proc. Natl. Acad. Sci. U.S.A. 2012, 109, 9265–9270. 10.1073/pnas.1203417109. 22652572 PMC3386139

[ref48] Tsoureas N.; Summerscales O. T.; Cloke F. G. N.; Roe S. M. Steric effects in the reductive coupling of CO by mixed-sandwich uranium(III) complexes. Organometallics 2013, 32, 1353 10.1021/om301045k.

[ref49] Wang B.; Luo G.; Nishiura M.; Luo Y.; Hou Z. Cooperative trimerization of carbon monoxide by lithium and samarium boryls. J. Am. Chem. Soc. 2017, 139, 16967–16973. 10.1021/jacs.7b10108. 29083923

[ref50] Simler T.; McCabe K. N.; Maron L.; Nocton G. CO reductive oligomerization by a divalent thulium complex and CO2-induced functionalization. 2021, ChemRxiv (accessed June 22, 2022). 10.1039/d2sc01798a PMC9241974 35919756

[ref51] aHicks J.; Vasko P.; Goicoechea J. M.; Aldridge S. Synthesis, structural and reaction chemistry of a nucleophilic aluminyl anion. Nature 2018, 557, 92–95. 10.1038/s41586-018-0037-y. 29662211

[ref52] aSchwamm R. J.; Anker M. D.; Lein M.; Coles M. P. Reduction vs. addition: the reaction of an aluminyl anion with 1,3,5,7-cyclooctatetraene. Angew. Chem., Int. Ed. 2019, 58, 1489–1493. 10.1002/anie.201811675. 30548141

[ref53] Hicks J.; Vasko P.; Goicoechea J. M.; Aldridge S. Reversible, room-temperature C-C bond activation of benzene by an isolable metal complex. J. Am. Chem. Soc. 2019, 141, 11000–11003. 10.1021/jacs.9b05925. 31251586

[ref54] Hicks J.; Vasko P.; Heilmann A.; Goicoechea J. M.; Aldridge S. Arene C-H activation at aluminium(I): meta selectivity driven by the electronics of S_N_Ar chemistry. Angew. Chem., Int. Ed. 2020, 59, 20376–20380. 10.1002/anie.202008557. PMC7693242 32722863

[ref55] Heilmann A.; Hicks J.; Vasko P.; Goicoechea J. M.; Aldridge S. Carbon monoxide activation by a molecular aluminium imide: C-O Bond cleavage and C-C bond formation. Angew. Chem., Int. Ed. 2020, 59, 4897–4901. 10.1002/anie.201916073. 31999037

[ref56] Hicks J.; Heilmann A.; Vasko P.; Goicoechea J. M.; Aldridge S. Trapping and reactivity of a molecular aluminium oxide ion. Angew. Chem., Int. Ed. 2019, 58, 17265–17268. 10.1002/anie.201910509. 31550066

[ref57] Roy M. M. D.; Hicks J.; Vasko P.; Heilmann A.; Baston A. M.; Goicoechea J. M.; Aldridge S. Probing the extremes of covalency in M–Al bonds: lithium and zinc aluminyl compounds. Angew. Chem., Int. Ed. 2021, 60, 22301–22306. 10.1002/anie.202109416. 34396660

[ref58] Anker M. D.; McMullin C. L.; Rajabi N. A.; Coles M. P. Carbon–carbon bond forming reactions promoted by aluminyl and alumoxane anions: introducing the ethenetetraolate ligand. Angew. Chem., Int. Ed. 2020, 59, 12806–12810. 10.1002/anie.202005301. 32378311

[ref59] aGanesamoorthy C.; Schoening J.; Wölper C.; Song L.; Schreiner P. R.; Schulz S. A silicon-carbonyl complex stable at room temperature. Nat. Commun. 2020, 12, 608–614. 10.1038/s41557-020-0456-x. 32313239

[ref60] Mitoraj M. P.; Michalak A.; Ziegler T.; A combined charge and energy decomposition scheme for bond analysis. J. Chem. Theory Comput. 2009, 5, 962–975. 10.1021/ct800503d. 26609605

[ref61] Heitkemper T.; Sindlinger C. P. A cationic NHC-supported borole. Chem.—Eur. J. 2020, 26, 11684–11689. 10.1002/chem.202001916. 32343437 PMC7540045

[ref62] For a previous example of a transformation of CO proceeding through an O-bound adduct, see for example Berkefeld A.; Piers W. E.; Parvez M.; Castro L.; Maron L.; Eisenstein O. Carbon monoxide activation via O-Bound CO using decamethylscandocinium–hydridoborate ion pairs. J. Am. Chem. Soc. 2012, 134, 10843–10851. 10.1021/ja300591v. 22670831

[ref63] Schleyer P. v. R.; Najafian K.; Kiran B.; Jiao H. Are oxocarbon dianions aromatic?. J. Org. Chem. 2000, 65, 426–431. 10.1021/jo991267n. 10813951

[ref64] Kollmar H.; Staemmler V. A theoretical study of the structure of cyclobutadiene. J. Am. Chem. Soc. 1977, 99, 3583–3587. 10.1021/ja00453a009.

[ref65] Hill-Cousins J. T.; Pop I.-A.; Pileio G.; Stevanato G.; Håkansson P.; Roy S. S.; Levitt M. H.; Brown L. J.; Brown R. C. D. Synthesis of an isotopically labeled naphthalene derivative that supports a long-lived nuclear singlet state. Org. Lett. 2015, 17, 2150–2153. 10.1021/acs.orglett.5b00744. 25898076 PMC4516318

