# Supplementary material for: Coordination and Homologation of CO at Al(I): Mechanism and Chain Growth, Branching, Isomerization, and Reduction
Source: J Am Chem Soc. 2022 Jul 5;144(28):12942–53. doi: 10.1021/jacs.2c05228 (PMC9348839; doi:10.1021/jacs.2c05228)
Supplement: Supplementary file 1 — ja2c05228_si_001.pdf [file ja2c05228_si_001.pdf]

Coordination and homologation of CO at Al(I): mechanism and chain growth, branching, isomerization and reduction

Andreas Heilmann, Matthew M. D. Roy, Agamemnon E. Crumpton, Liam P. Griffin, Jamie Hicks, Jose M. Goicoechea,* and Simon Aldridge*

Supporting Information (54 pages)

Table of contents:

1 General considerations S2
2 Sources of starting materials S2
3 Synthetic procedures and characterising data for new compounds S3
4 Representative NMR spectra S7
5 X-ray crystallographic studies S14
6 Computational details S25
7 References S53

1 General considerations:

All manipulations were carried out using standard Schlenk line or dry-box techniques under an atmosphere of argon or dinitrogen. Et_2O and THF were distilled from sodium or potassium and benzophenone. All other solvents were degassed by sparging with argon and dried by passing through a column of the appropriate drying agent. NMR spectra were measured in benzene-d_6 (which was dried over potassium) or THF-d_8 (which was dried over CaH_2), with the solvent then being distilled under reduced pressure and stored under argon in Teflon valve ampoules. NMR samples were prepared under argon in 5 mm Wilmad 507-PP tubes fitted with J. Young Teflon valves. ^1H and $^{13}C\{^1H\}$ NMR spectra were recorded on Bruker Avance III HD nanobay 400 MHz, Bruker Avance III 500 MHz, or Bruker NEO 600 MHz spectrometers at ambient temperature and referenced internally to residual protio-solvent (^1H) or solvent (^{13}C) resonances and are reported relative to tetramethylsilane (δ = 0 ppm). Assignments were confirmed using two-dimensional ^1H-^1H and ^{13}C-^1H NMR correlation experiments. Chemical shifts are quoted in δ (ppm) and coupling constants in Hz. Elemental analyses were carried out by Elemental Microanalysis Ltd, Okehampton, Devon, UK.

2 Sources of starting materials:

NONAlI[S1a], K_2[**NON**Al]$_2$[S1a], [Li(OEt_2)$_2$][**NON**Al][S1b] and Ag_2(squarate)[S2] were prepared according to literature methods. All other reagents were used as received.

3 Synthetic procedures and characterising data for new compounds:

$[K(C_7H_8)]_2[\{(NON)Al\}_2(C_4O_4)]$, $[$2$-K(C_7H_8)]_2$: To a 25 mL reaction bomb was added [**1**-K]$_2$ (250 mg, 0.34 mmol) and benzene (7.5 mL). The resulting mixture was degassed twice using the freeze-pump-thaw method, and sealed under vacuum. The bomb was then warmed to 307 K in an oil bath, and after a few minutes the headspace was charged with CO (1.5 atm, ca. 1.2 mmol). After sealing the vessel, the mixture was vigorously shaken for 30 s, and subsequently allowed to stand undisturbed at 307 K for 16 h, leading to the formation of small orange crystals of [**2**-K(C_7H_8)]$_2$·4(C_7H_8). Isolated yield of single crystals: 37 mg, 11 %. Anal. Calc. for $C_{98}H_{124}Al_2K_2N_4O_6$ + 1.5 toluene: C 75.57, H 7.95, N 3.25; found: C 75.52, H 8.04, N 3.22.

^{1}H NMR (400 MHz, THF-d$_8$) δ 7.07 – 6.95 (m, 6H, Dipp-Ar-C*H*), 6.42 (d, *J* = 1.9 Hz, 2H, XA-C*H*^{1}), 5.62 (d, *J* = 1.9 Hz, 2H, XA-C*H*^{3}), 3.30 – 3.14 (m, 4H, C*H*Me$_2$), 1.73 (s, 3H, C(C*H*$_3$)$_2$), 1.56 (s, 3H, C(CH$_3$)$_2$), 1.11 (d, *J* = 6.8 Hz, 6H, CH(C*H*$_3$)$_2$), 1.04 (s, 18H, C(C*H*$_3$)$_3$), 0.86 (d, *J* = 6.8 Hz, 6H, CH(C*H*$_3$)$_2$), 0.68 (d, *J* = 6.8 Hz, 6H, CH(C*H*$_3$)$_2$), 0.43 (d, *J* = 6.8 Hz, 6H, CH(C*H*$_3$)$_2$).
^{13}C NMR (126 MHz, THF-d$_8$) δ 148.2 (Dipp-*o*-*C*), 147.8 (*C*^{t}Bu), 147.6 (Dipp-*o*-*C*), 146.8 (XA-*C*N), 144.8 (Dipp-*i*-*C*), 142.5 (XA-*C*O), 133.8 (*C*CMe$_2$), 131.3 (C(=O)*C*O), 129.2 (C$_6$H$_6$), 125.4 (Dipp-Ar-*C*H), 125.2 (Dipp-Ar-*C*H), 123.6 (Dipp-*m*-*C*H), 111.0 (XA-*C*^{3}H), 105.3 (XA-*C*^{1}H), 37.9 (*C*CMe$_2$), 35.5 (*C*Me$_3$), 32.6 (C(*C*H$_3$)$_2$), 32.2 (C(*C*H$_3$)$_3$), 29.1 (*C*H(CH$_3$)$_2$), 28.5 (*C*H(CH$_3$)$_2$), 26.3 (CH(*C*H$_3$)$_2$), 25.5* (CH(*C*H$_3$)$_2$), 24.7 (CH(*C*H$_3$)$_2$), 24.5 (CH(*C*H$_3$)$_2$), 23.8 (C(*C*H$_3$)$_2$). *Overlapped with solvent signal – located in HSQC and HMBC. Due to quadrupolar broadening the resonance associated with the Al-*C*(=O) group was not observed in the range -30 to 300 ppm. The corresponding signal is measured for the related derivative [**2**-K(12-crown-4)]$_2$ at 296.2 ppm.

$[K(12\text{-}c\text{-}4)]_2[\{(NON)Al\}_2(C_4O_4)]$, $[$2$-K(12\text{-}c\text{-}4)]_2$: Method 1: To a suspension of [**2**-K(C_7H_8)]$_2$ (15 mg, 0.009 mmol) in benzene-d$_6$ (0.5 ml) was added a solution of 12-crown-4 in toluene (0.18 mL, 0.1 M, 0.018 mmol, 2.05 equiv.) and the reaction mixture heated at 358 K for 2 h. Volatiles were removed under vacuum and the product was obtained as an orange powder. Yield 13 mg, 80 % (quantitative by NMR).
Method 2: To a solution of [**1**-K]$_2$ (0.120 g, 0.163 mmol) in toluene (5 mL) was added a solution of 12-crown-4 in toluene (3.2 mL of a 0.1 M solution, 0.320 mmol). The resulting red solution was stirred at room temperature for 5 min, before addition of Fe(CO)$_5$ (22 µL, 0.161 mmol). The resulting dark red solution was stirred overnight. Removal of volatiles yields a yellow oil which can be dissolved in minimal benzene and stored at room temperature overnight to yield single crystals of [**2**-K(12-c-4)]$_2$·4(C_6H_6) suitable for X-ray crystallography. Isolated yield of single crystals ca. 5 mg, 20% (30 % by NMR).

^{1}H NMR (600 MHz, THF-d$_8$) δ 7.01 (t, *J* = 7.0 Hz, 2H, Dipp-*p*-C*H*), 6.96 (d, *J* = 7.0 Hz, 4H, Dipp-*m*-C*H*), 6.40 (d, *J* = 2.0 Hz, 2H, XA-C*H*^{1}), 5.59 (d, *J* = 1.9 Hz, 2H, XA-C*H*^{3}), 3.60 (s, 16H, 12-crown-4), 3.42 – 3.34 (m, 2H, C*H*Me$_2$), 3.14 (sept., *J* = 6.9 Hz, 2H, C*H*Me$_2$), 1.73 (s, 3H, C(C*H*$_3$)$_2$), 1.58 (s, 3H, C(C*H*$_3$)$_2$), 1.24 (d, *J* = 6.7 Hz, 6H, CH(C*H*$_3$)$_2$), 1.03 (s, 18H, C(C*H*$_3$)$_3$), 0.79 (d, *J* = 6.8 Hz, 6H, CH(C*H*$_3$)$_2$), 0.77 (d, *J* = 6.8 Hz, 6H, CH(C*H*$_3$)$_2$), 0.33 (d, *J* = 6.8 Hz, 6H, CH(C*H*$_3$)$_2$).
^{13}C NMR (151 MHz, THF-d$_8$) δ 296.2 (Al*C*O), 147.7 (Dipp-*o*-*C*), 147.5 (Dipp-*o*-*C*), 147.4 (*C*^{t}Bu), 146.9 (XA-*C*N), 145.2 (Dipp-*i*-*C*), 142.7 (XA-*C*O), 134.1 (*C*CMe$_2$), 130.8 (C(=O)*C*O), 125.9 (Dipp-*m*-*C*H), 125.1 (Dipp-*p*-*C*H), 123.1 (Dipp-*m*-*C*H), 111.4 (XA-*C*^{3}H), 105.3 (XA-*C*^{1}H), 70.5 (O*C*H$_2$),

37.8 (*C*Me$_2$), 35.4 (*C*(CH$_3$)$_3$), 32.2 (C(*C*H$_3$)$_3$), 32.0 (C(*C*H$_3$)$_2$), 29.4 (*C*HMe$_2$), 28.7 (*C*HMe$_2$), 26.5 (CH(*C*H$_3$)$_2$), 25.0 (CH(*C*H$_3$)$_2$), 24.6 (CH(*C*H$_3$)$_2$), 24.3 (CH(*C*H$_3$)$_2$), 23.6 (C(*C*H$_3$)$_2$).

[Li(OEt$_2$)][{(NON)Al}$_2$(C$_4$O$_4$)], [2-Li(OEt$_2$)]$_2$ and [Li(THF)]$_2$[{(NON)Al}$_2$(C$_4$O$_4$)], [2-Li(THF)]$_2$: Ether adduct: (Method 1) A solution of [**1**-Li] (15 mg, 0.017 mmol) in C_6D_6 (0.5 mL) was degassed twice using the freeze-pump-thaw method. The headspace was then charged with CO (2 bar) and briefly shaken. ^{1}H NMR reveals formation of a mixture of products, including [**2**-Li(OEt$_2$)]$_2$ in ca. 40% yield. Upon concentration (to ca. 1/3 volume) and standing for two days, X-ray quality crystals of [**2**-Li(OEt$_2$)]$_2$ formed.
(Method 2) [**2**-K(C$_7$H$_8$)]$_2$ (30 mg, 0.018 mmol) and LiI (5 mg, 2.1 equiv.) were dissolved in Et$_2$O (15 mL) and briefly heated to 318 K in a sealed ampoule, leading to a colour change from orange to yellow. The mixture was allowed to cool to room temperature and the resulting cloudy mixture was filtered onto benzene (8 mL). After mixing, the solution was concentrated until it turned turbid (ca. 5 mL). At this point a sample (0.2 mL) was taken and diluted with C_6D_6 (0.3 ml) and a ^{1}H NMR spectrum measured. The resulting spectrum shows the presence of a single species, which has the same spectroscopic signals as those obtained using method 1 in a J. Young's NMR tube.

^{1}H NMR (400 MHz, C_6D_6) δ 6.67 (d, *J* = 2.0 Hz, 2H, XA-C*H*^{1}), 6.00 (d, *J* = 2.0 Hz, 2H, XA-C*H*^{3}), 3.72 (sept, *J* = 6.9 Hz, 2H), 1.70 (s, 3H, C(C*H*$_3$)$_2$), 1.61 (s, 3H, C(C*H*$_3$)$_2$), 1.52 (d, *J* = 6.9 Hz, 6H, CH(C*H*$_3$)$_2$), 1.19 (s, 18H, C(C*H*$_3$)$_3$), 0.36 (d, *J* = 6.9 Hz, 6H, CH(C*H*$_3$)$_2$). Samples of [**2**-Li(OEt$_2$)]$_2$ lose Et$_2$O under continuous vacuum. Dissolution in THF-d$_8$, however, yields spectroscopic signals indistinguishable from samples of [**2**-Li(THF)]$_2$ prepared as set out below.

THF adduct: A solution containing [**2**-K(C$_7$H$_8$)]$_2$ (ca. 20 mg, 0.012 mmol) was prepared as described above and Li[HB^{s}Bu$_3$] (1 mL of a 1 M solution in THF) was added. The mixture was heated at 307 K for 16 h affording small yellow crystals. Analytically pure samples can be obtained by recrystallization from minimal benzene or THF and washing with THF. Isolated yield of single crystals: 7 mg, 30% over last step. Anal. Calc. for $C_{106}H_{140}Al_2Li_2N_4O_8$ + 1.75 THF: C 75.73, H 8.66, N 3.13; found: C 75.13, H 8.66, N 3.21.

^{1}H NMR (400 MHz, C_6D_6) δ 7.25 (m, 6H, Dipp-Ar-C*H*), 6.70 (d, *J* = 2.0 Hz, 2H, XA-C*H*^{1}), 6.05 (d, *J* = 2.0 Hz, 2H, XA-C*H*^{3}), 3.77 (sept, *J* = 6.9 Hz, 2H, C*H*Me$_2$), 3.70 (s, m, 4H, THF), 3.41 (sept, *J* = 6.9 Hz, 2H, C*H*Me$_2$), 1.69 (s, 3H, C(C*H*$_3$)$_2$), 1.60 (s, 3H, C(C*H*$_3$)$_2$), 1.58 (d, *J* = 6.9 Hz, 6H, CH(C*H*$_3$)$_2$), 1.32 (m, 4H, THF), 1.21 (s, 18H, C(C*H*$_3$)$_3$), 1.15 (overlapping d, *J* = 6.9 Hz, 12H, CH(C*H*$_3$)$_2$), 0.36 (d, *J* = 6.9 Hz, 6H, CH(C*H*$_3$)$_2$).
^{13}C NMR (151 MHz, C_6D_6) δ 147.8 (*C*^{t}Bu), 147.2 (Dipp-*o*-*C*), 146.3 (Dipp-*o*-*C*), 145.7 (XA-*C*^{4}), 143.6 (Dipp-*i*-*C*), 141.4 (XA-*C*O), 133.1 (*C*CMe$_2$), 125.8 (Dipp-*p*-*C* and Dipp-*m*-*C*), 123.0 (Dipp-*m*-*C*), 111.3 (XA-*C*^{3}H), 106.4 (XA-*C*^{1}H), 68.6 (THF-*C*O), 37.0 (*C*Me$_2$), 35.0 (*C*Me$_3$), 32.1 (C(*C*H$_3$)$_2$), 31.9 (C(*C*H$_3$)$_3$), 29.5 (*C*HMe$_2$), 28.2 (*C*HMe$_2$), 26.3 (CH(*C*H$_3$)$_2$), 25.4 (THF), 24.1 (CH(*C*H$_3$)$_2$), 23.9 (CH(*C*H$_3$)$_2$), 23.2 (C(*C*H$_3$)$_2$), 23.2 (CH(*C*H$_3$)$_2$). Due to quadrupolar broadening (and overlap with the solvent) the resonances associated with the $[C_4O_4]^{4-}$ fragment were not observed in the range -30 to 300 ppm. The corresponding signals are measured for the related derivative [**2**-K(12-crown-4)]$_2$ at 130.8 and 296.2 ppm. ^{7}Li NMR (156 MHz, C_6D_6) δ 0.61.

K[K(THF)$_2$][{(NON)Al}$_2$(C$_6$O$_6$)], 3: [**2**-K(C$_7$H$_8$)]$_2$ (25 mg, 0.015 mmol) was suspended in THF-d$_8$ (0.5 mL) in a J. Youngs NMR tube and degassed twice using the freeze-pump-thaw method.

The headspace was then charged with CO (2 atm) and heated at 339 K for 4 d. During this time [**2**-K(C_7H_8)]$_2$ dissolved and a colour change from yellow to red to orange was observed. ^{1}H NMR monitoring shows quantitative conversion to a single product. After concentrating the solution to a quarter of its original, small pale orange plates formed at the surface of the solution. Anal. Calc. for $C_{108}H_{140}Al_2K_2N_4O_{10}$ + 2 THF: C 72.16, H 8.14, N 2.90; found: C 72.13, H 8.06, N 2.55.

^{1}H NMR (600 MHz, THF-d$_8$) δ 7.43 (t, *J* = 7.6 Hz, 2H, Dipp-*p*-C*H*), 7.32 (t, *J* = 7.6 Hz, 2H, Dipp-*p*-C*H*), 7.25 (dd, *J* = 7.8, 1.6 Hz, 2H, Dipp-*m*-C*H*), 7.16 (dd, *J* = 7.6, 1.6 Hz, 2H, Dipp-*m*-C*H*), 7.09 (dd, *J* = 7.8, 1.6 Hz, 2H, Dipp-*m*-C*H*), 7.00 (dd, *J* = 7.7, 1.6 Hz, 2H, Dipp-*m*-C*H*), 6.49 (d, *J* = 1.9 Hz, 2H, XA-C^1*H*), 6.42 (d, *J* = 1.9 Hz, 2H, XA-C^1*H*), 5.36 (d, *J* = 1.9 Hz, 2H, XA-C^3*H*), 5.34 (d, *J* = 1.9 Hz, 2H, XA-C^3*H*), 3.46 – 3.36 (overlapping sept., 4H, C*H*Me$_2$), 3.32 (sept., *J* = 7.2 Hz, 2H, C*H*Me$_2$), 3.16 (sept., *J* = 6.9 Hz, 2H, C*H*Me$_2$), 1.75 (s, 3H, C(C*H*$_3$)$_2$), 1.73 (s, 3H, C(C*H*$_3$)$_2$), 1.57 (s, 3H, C(C*H*$_3$)$_2$), 1.54 (s, 3H, C(C*H*$_3$)$_2$), 1.20 (d, *J* = 7.0 Hz, 6H, CH(C*H*$_3$)$_2$), 1.03 (s, 18H, C(C*H*$_3$)$_3$), 1.00 (s, 18H, C(C*H*$_3$)$_3$), 0.95 (d, *J* = 6.9 Hz, 6H, CH(C*H*$_3$)$_2$), 0.87 (d, *J* = 6.7 Hz, 6H, CH(C*H*$_3$)$_2$), 0.83 (d, *J* = 6.8 Hz, 6H, CH(C*H*$_3$)$_2$), 0.80 (d, *J* = 6.7 Hz, 6H, CH(C*H*$_3$)$_2$), 0.73 (d, *J* = 6.7 Hz, 6H, CH(C*H*$_3$)$_2$), 0.68 (d, *J* = 6.8 Hz, 6H, CH(C*H*$_3$)$_2$), 0.51 (d, *J* = 6.8 Hz, 6H, CH(C*H*$_3$)$_2$).
^{13}C NMR (151 MHz, THF-d$_8$) δ = 258.6 (C_6O_6), 173.5 (C_6O_6), 169.0 (C_6O_6), 156.2 (C_6O_6), 149.4 (Dipp-*o*-*C*), 149.1 (Dipp-*o*-*C*), 148.0 (Dipp-*o*-*C*), 147.6 (*C*^{t}Bu), 147.5 (Dipp-*o*-*C*), 147.3 (*C*^{t}Bu), 146.1 (XA-*C*^{4}), 145.8 (Dipp-*i*-*C*), 145.7(Dipp-*i*-*C*), 145.6 (XA-*C*^{4}), 142.7 (XA-*C*O), 141.5 (XA-*C*O), 133.7 (*C*CMe$_2$), 133.3 (*C*CMe$_2$), 129.2 (free C_6H_6), 127.4 (Dipp-*p*-*C*H), 127.3 (Dipp-*p*-*C*H), 127.2 (Dipp-*m*-*C*H), 126.2 (Dipp-*m*-*C*H), 124.4 (Dipp-*m*-*C*H), 123.8 (Dipp-*m*-*C*H), 118.2 (C_6O_6), 111.3 (XA-*C*^{3}H), 110.5 (C_6O_6), 106.5 (XA-*C*^{1}H), 105.9 (XA-*C*^{1}H), 37.9 (*C*Me$_2$), 37.8 (*C*Me$_2$), 35.5 (*C*Me$_3$), 35.4 (*C*Me$_3$), 32.2 (C(*C*H$_3$)$_3$), 32.1 (C(*C*H$_3$)$_3$), 32.1 (C(*C*H$_3$)$_2$), 32.0 (C(*C*H$_3$)$_2$), 29.2 (*C*HMe$_2$), 28.8 (*C*HMe$_2$), 28.1 (*C*HMe$_2$), 28.0 (*C*HMe$_2$), 26.2 (CH(*C*H$_3$)$_2$), 26.1 (CH(*C*H$_3$)$_2$), 26.0 (CH(*C*H$_3$)$_2$), 25.3 (CH(*C*H$_3$)$_2$), 25.2 (CH(*C*H$_3$)$_2$), 24.9 (CH(*C*H$_3$)$_2$), 22.9 (C(*C*H$_3$)$_2$), 22.9 (C(*C*H$_3$)$_2$).

K$_4$[{(NON)Al}$_2$(C$_4$O$_4$)(BEt$_3$)$_2$], [4-K$_2$(BEt$_3$)]$_2$: To a stirred solution containing [**1**-K]$_2$ (220 mg, 0.30 mmol), benzene (6.6 mL), and CO (ca. 1.5 bar) in a 25 mL reaction bomb was added K[HBEt$_3$] (1.5 mL of a 1M solution in THF, 1.5 mmol). After 24 h, the solution was concentrated to a fifth of its original volume under reduced pressure. Prolonged standing led to the formation of colourless extremely sensitive crystals of [**4**-K$_2$(BEt$_3$)]$_2$. Yield 12 mg, 4%.

^{1}H NMR (400 MHz, THF-d$_8$) δ 7.27 (br s, 6H, Dipp-Ar-C*H*), 6.36 (br s, 2H, XA-C*H*^{1}), 5.28 (s, 2H, XA-C*H*^{3}), 3.79 (br s, 2H, C*H*Me$_2$), 3.47 (sept, J = 6.6 Hz, 2H, C*H*Me$_2$), 1.68 (s, 6H, C(C*H*$_3$)$_2$), 1.28 (br s, 6H, CH(C*H*$_3$)$_2$), 1.00 (s, 18H, C(C*H*$_3$)$_3$), 0.80 (d, *J* = 6.6 Hz, 13H), 0.57 (t, *J* = 7.7 Hz, 9H, BCH$_2$C*H*$_3$), 0.06 (q, *J* = 7.7 Hz, 6H, BC*H*$_2$CH$_3$).
Due to the low solubility and stability of this compound in solution, no satisfactory ^{11}B and ^{13}C spectra could be obtained.

[(NON)Al]$_2$(C$_4$O$_4$), 5: (NON)AlI (500 mg, 0.60 mmol, 2.0 eq) and $Ag_2(C_4O_4)$ (110 mg, 0.33 mmol) were suspended in Et_2O (10 mL) and the reaction mixture refluxed at 308 K for 48 h in the dark. Volatiles were removed in vacuo and the residue extracted into toluene (15 mL), filtered, and layered with hexane (15 mL). Upon standing for several days, crystals of **5** suitable for single crystal X-ray diffraction were obtained. Yield 180 mg, 39 %. Anal. Calc. for $C_{98}H_{124}Al_2N_4O_6$ + 0.5 hexane: C 78.21, H 8.51, N 3.61; found 78.08, H 8.76, N 3.34.

^{1}H NMR (400 MHz, C_6D_6) δ 7.20 (m, 2H, Dipp-*p*-C*H*), 7.14 (m, 4H, Dipp-*p*-C*H*), 6.72 (d, *J* = 1.9 Hz, 2H, XA-CH^1), 5.98 (d, *J* = 1.9 Hz, 2H, XA-CH^3), 3.54 (sept, *J* = 6.8 Hz, 4H, C*H*Me_2), 1.58 (s, 6H, C(CH_3)$_2$), 1.15 (s, 18H, C(CH_3)$_3$), 1.06 (d, *J* = 6.8 Hz, 12H, CH(CH_3)$_2$), 0.91 – 0.83 (br s, 12H, CH(CH_3)$_2$).
^{13}C NMR (126 MHz, C_6D_6) δ = 194.0 (C_4O_4), 148.9 (C^tBu), 147.0 (Dipp-*o*-*C*), 144.4 (XA-*C*N), 141.0, 140.8 (Dipp-*i*-*C*, XA-*C*O), 133.3 (*C*CMe_2), 127.0 (Dipp-*p*-*C*), 125.1 (Dipp-*m*-*C*), 112.1 (XA-C^3H), 108.2 (XA-C^1H), 37.2 (*C*Me_2), 35.1(*C*Me_3), 31.7 (C(*C*H_3)$_3$), 28.4 (*C*HMe_2), 26.4 (br, C(*C*H_3)$_2$), 25.4 (CH(*C*H_3)$_2$), 24.7(CH(*C*H_3)$_2$).

4 Representative NMR Spectra:

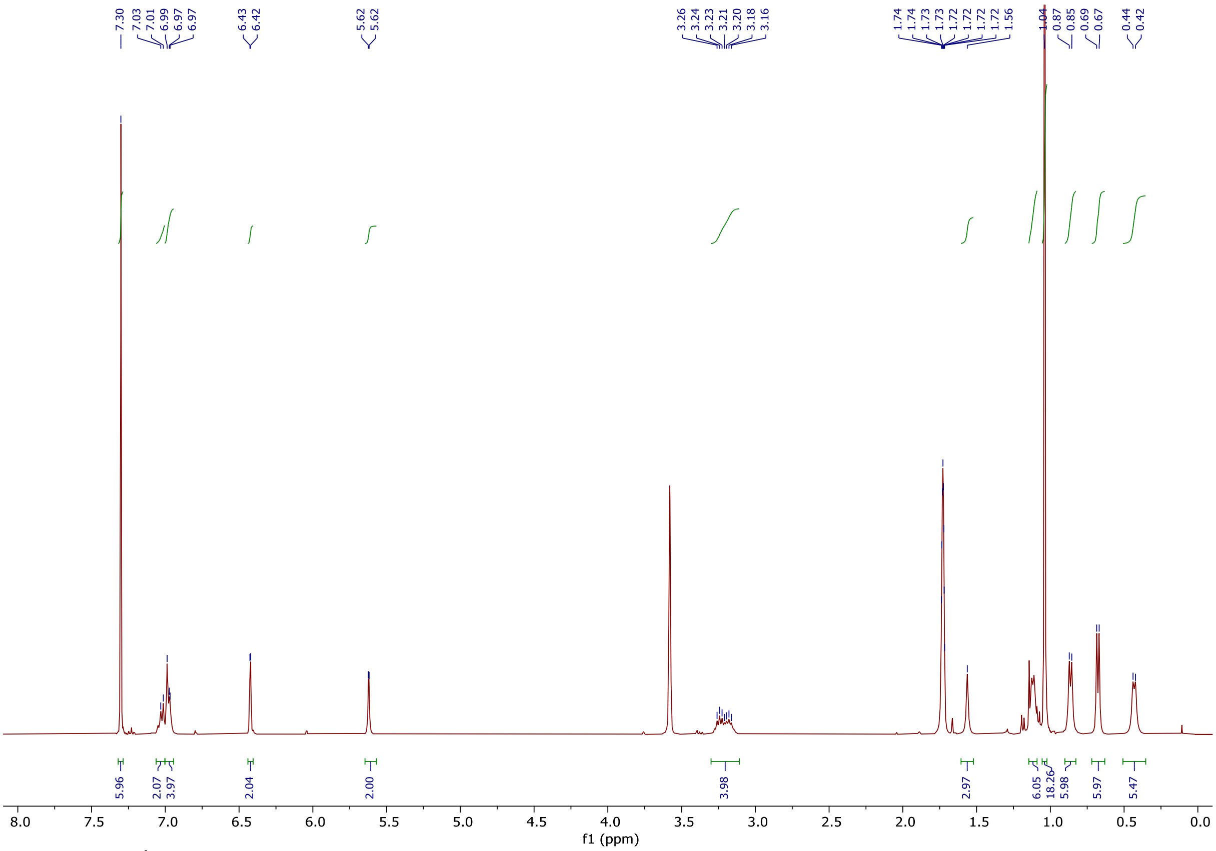

Figure S1: ^{1}H NMR spectrum of [**2**-K(C_7H_8)]$_2$ in THF-d_8.

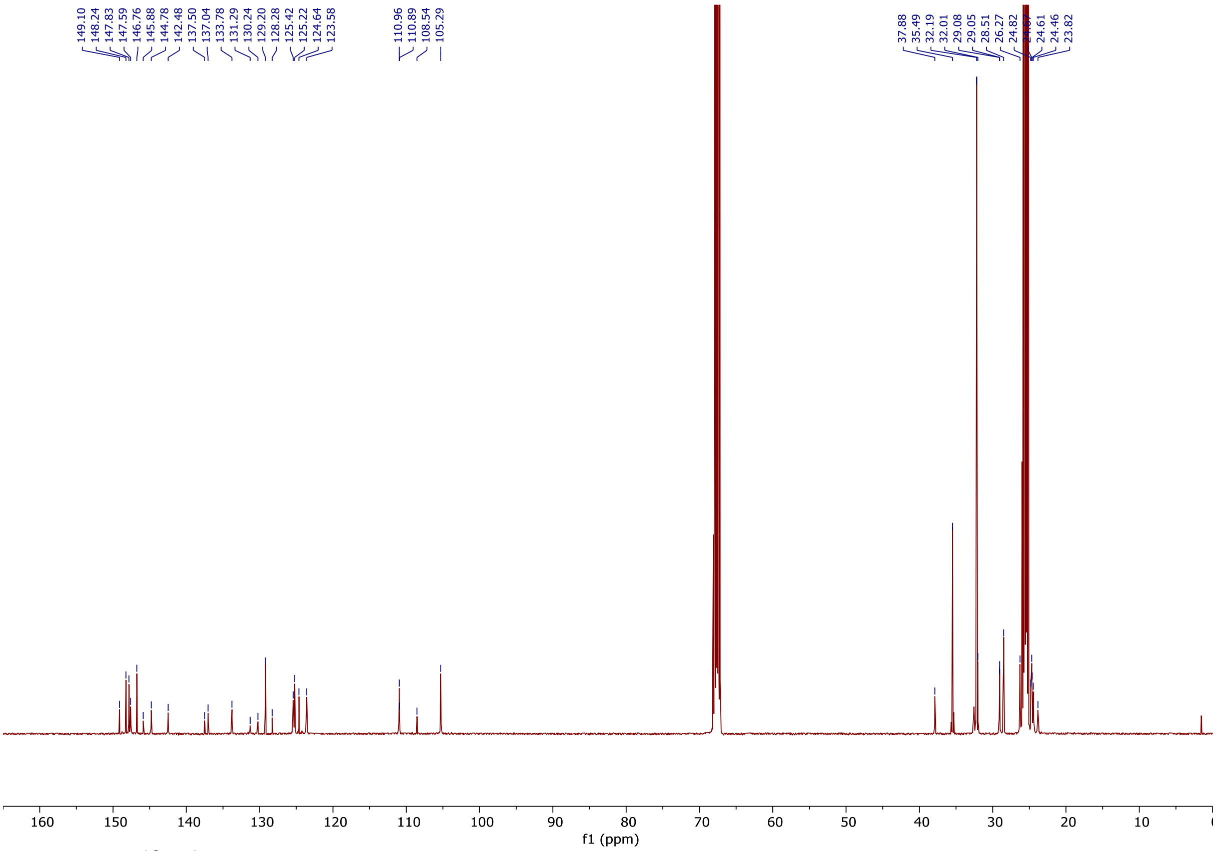

Figure S2: $^{13}C\{^{1}H\}$ NMR spectrum of [**2**-K(C_7H_8)]$_2$ in THF-d_8.

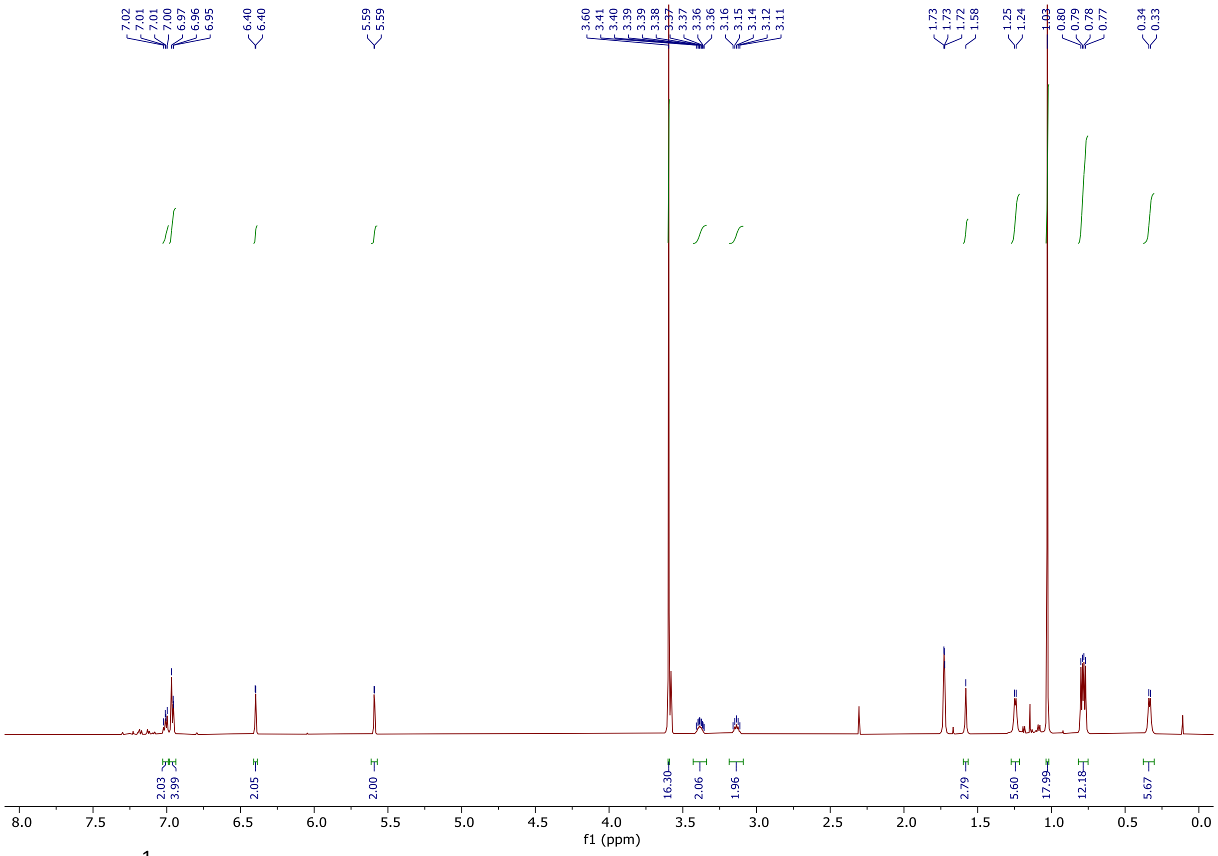

Figure S3: ^{1}H NMR spectrum of [**2**-K(12-c-4)]$_2$ in THF-d$_8$.

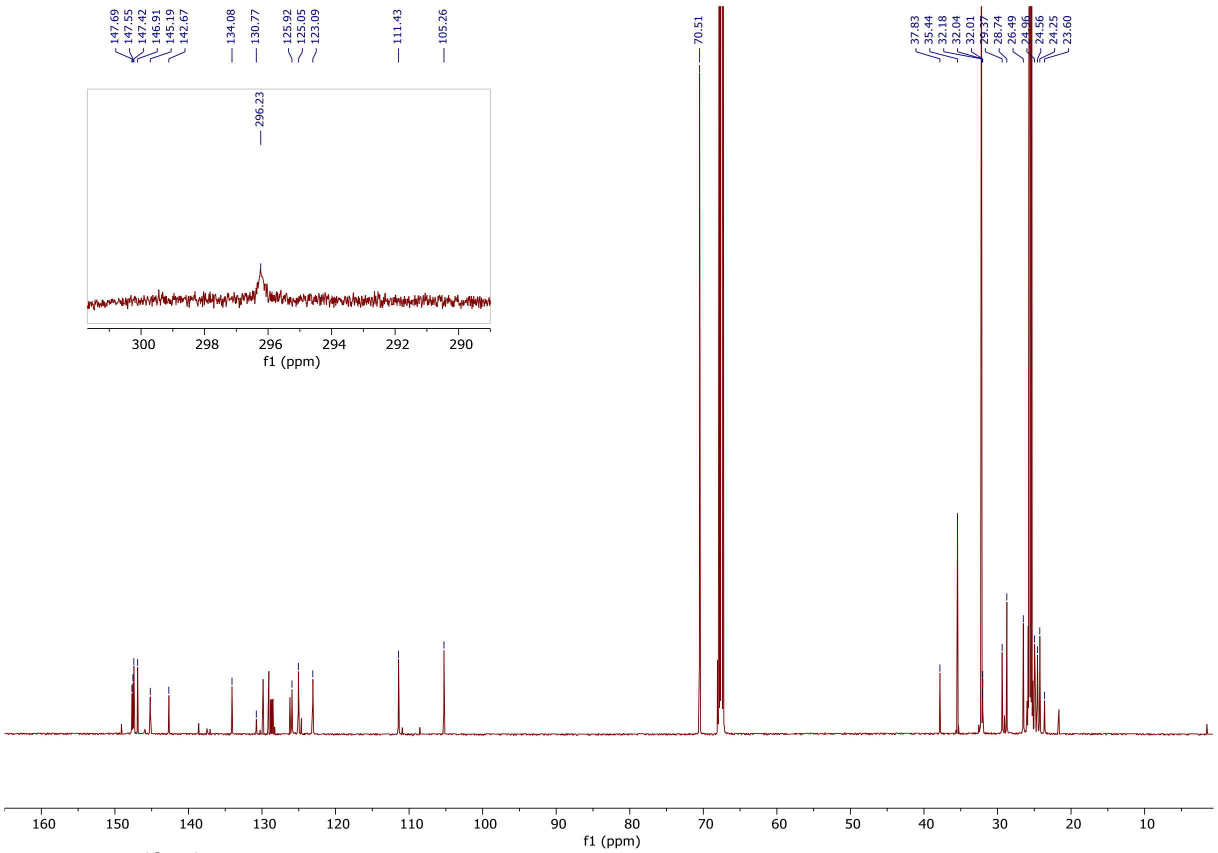

Figure S4: ^{13}C{^{1}H} NMR spectrum of [**2**-K(12-c-4)]$_2$ in THF-d$_8$.

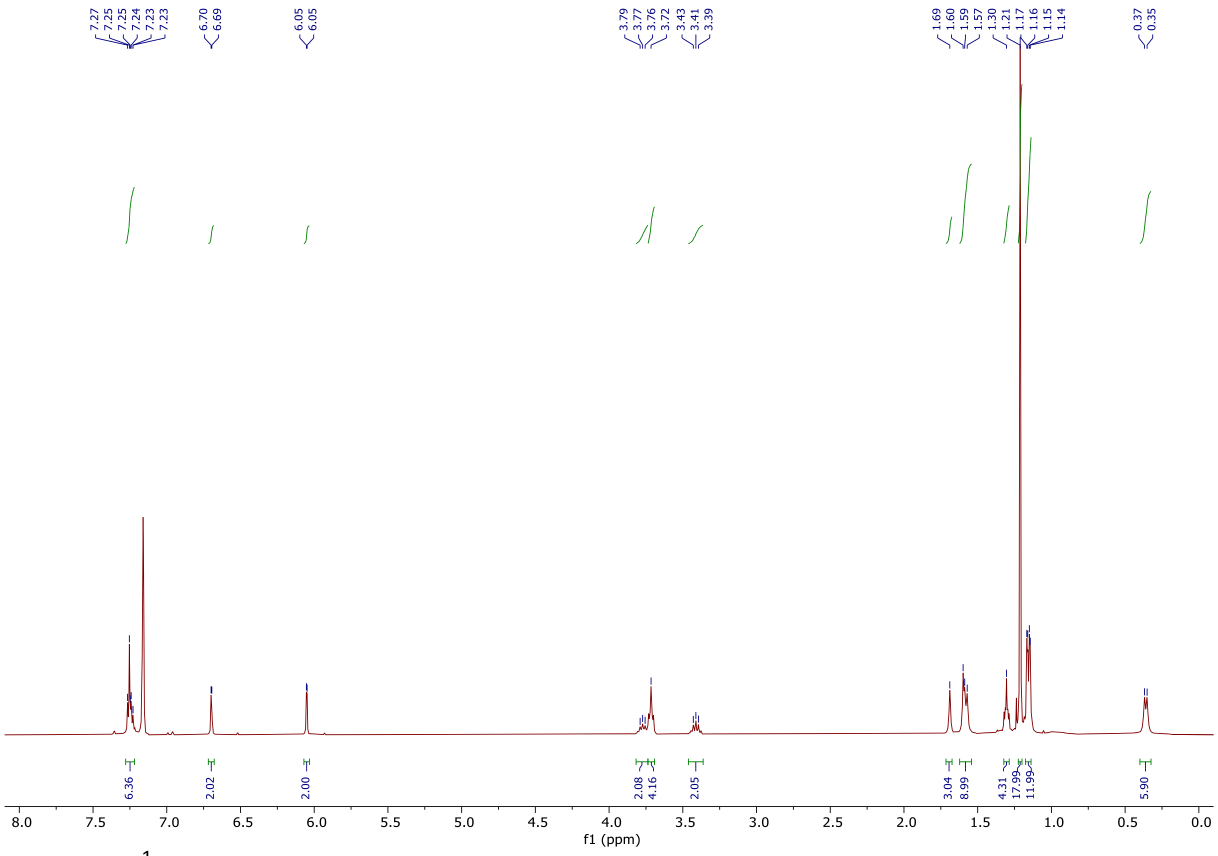

Figure S5: ^{1}H NMR spectrum of [**2**-Li(THF)]$_2$ in C_6D_6.

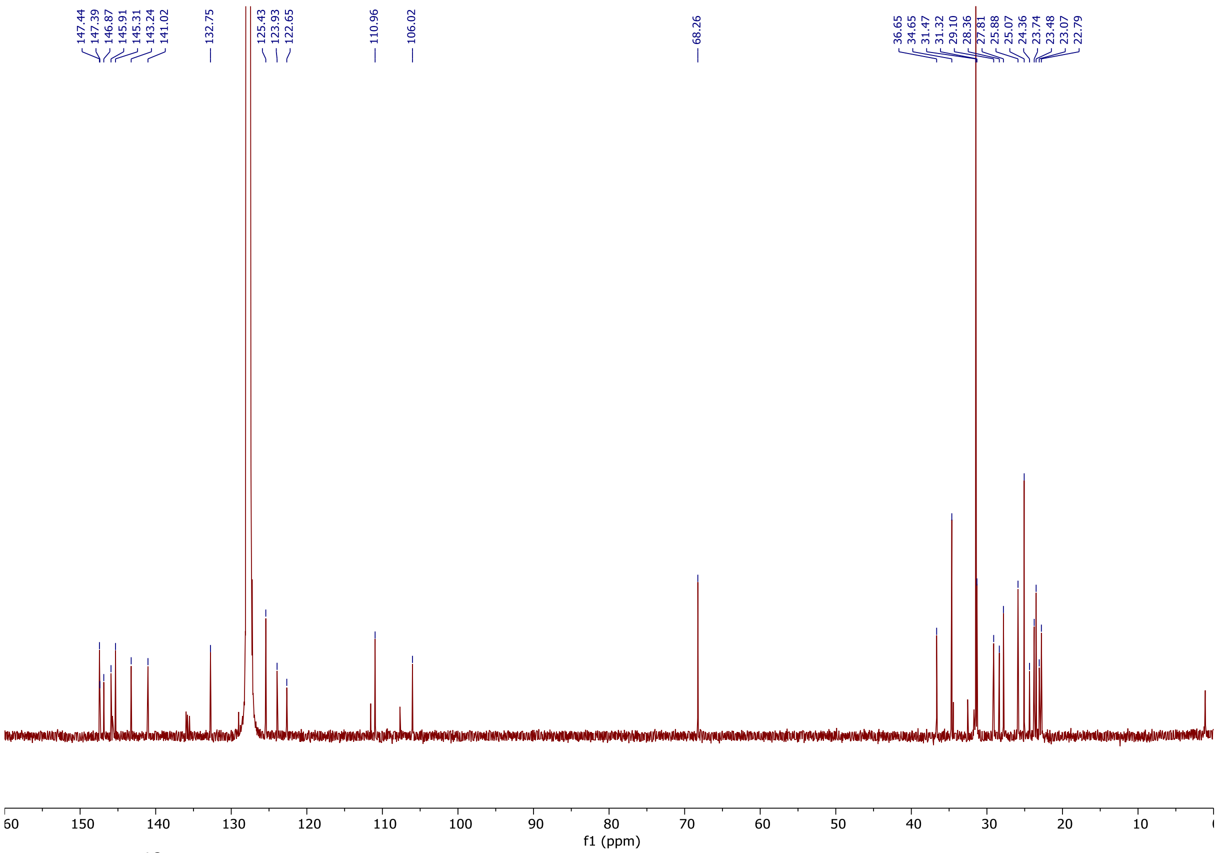

Figure S6: ^{13}C NMR spectrum of [**2**-Li(THF)]$_2$ in C_6D_6.

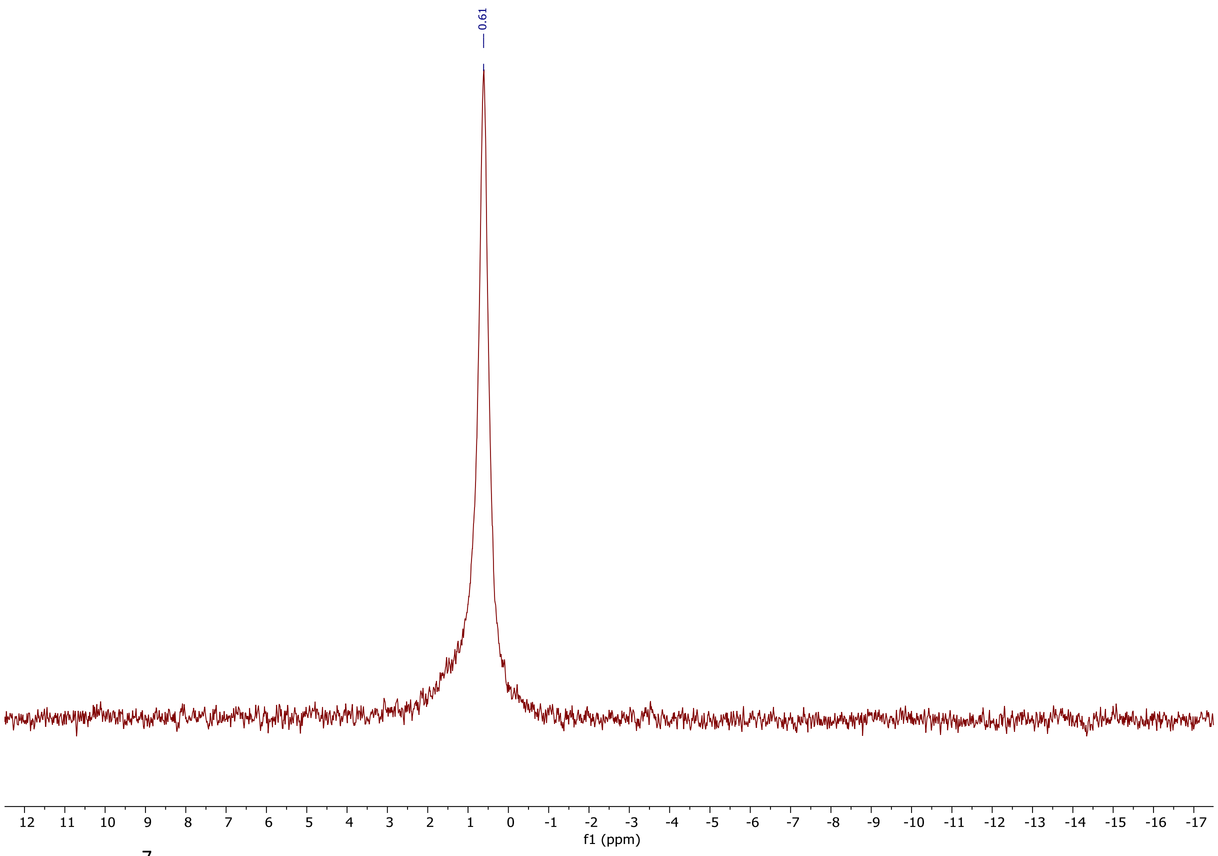

Figure S7: ^{7}Li NMR spectrum of [**2**-Li(THF)]$_2$ in C_6D_6.

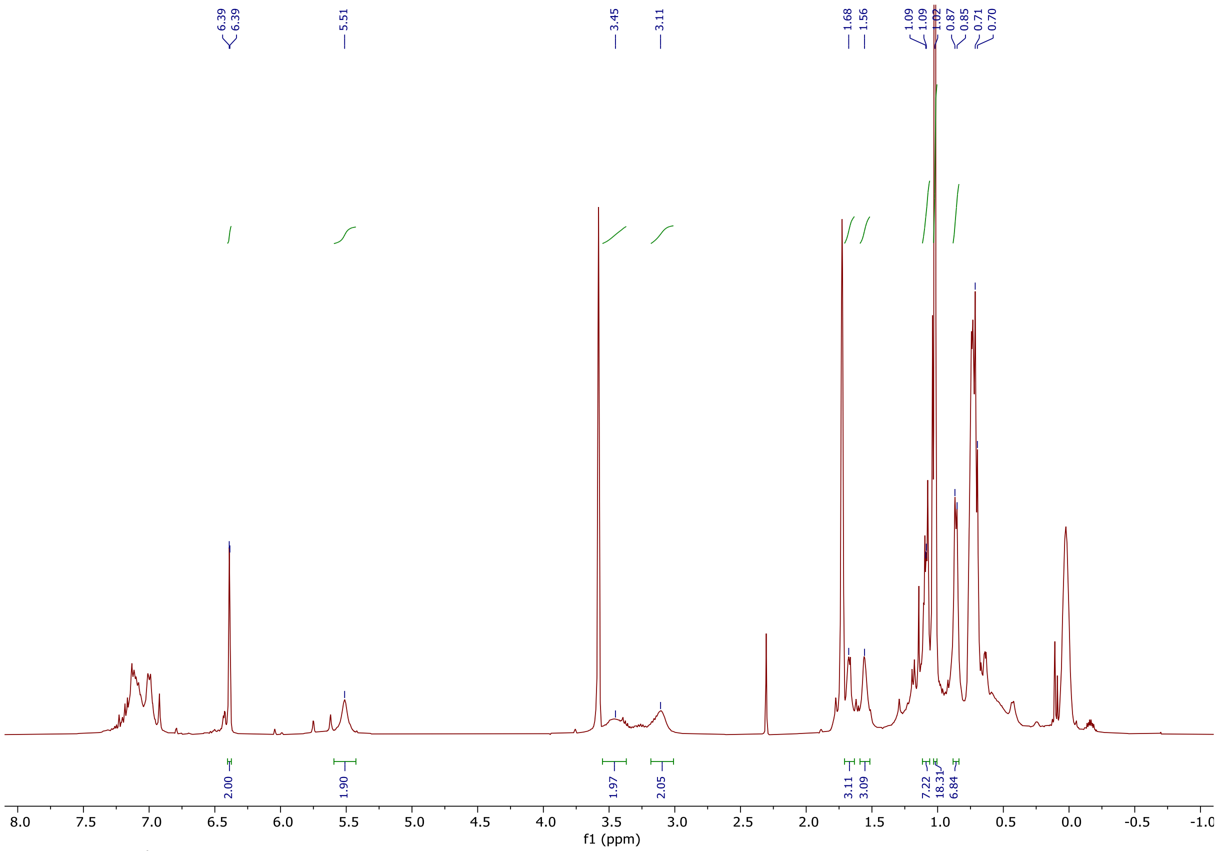

Figure S8: ^{1}H NMR spectrum of [**2**-K(THF)]$_2$ in THF-d_8.

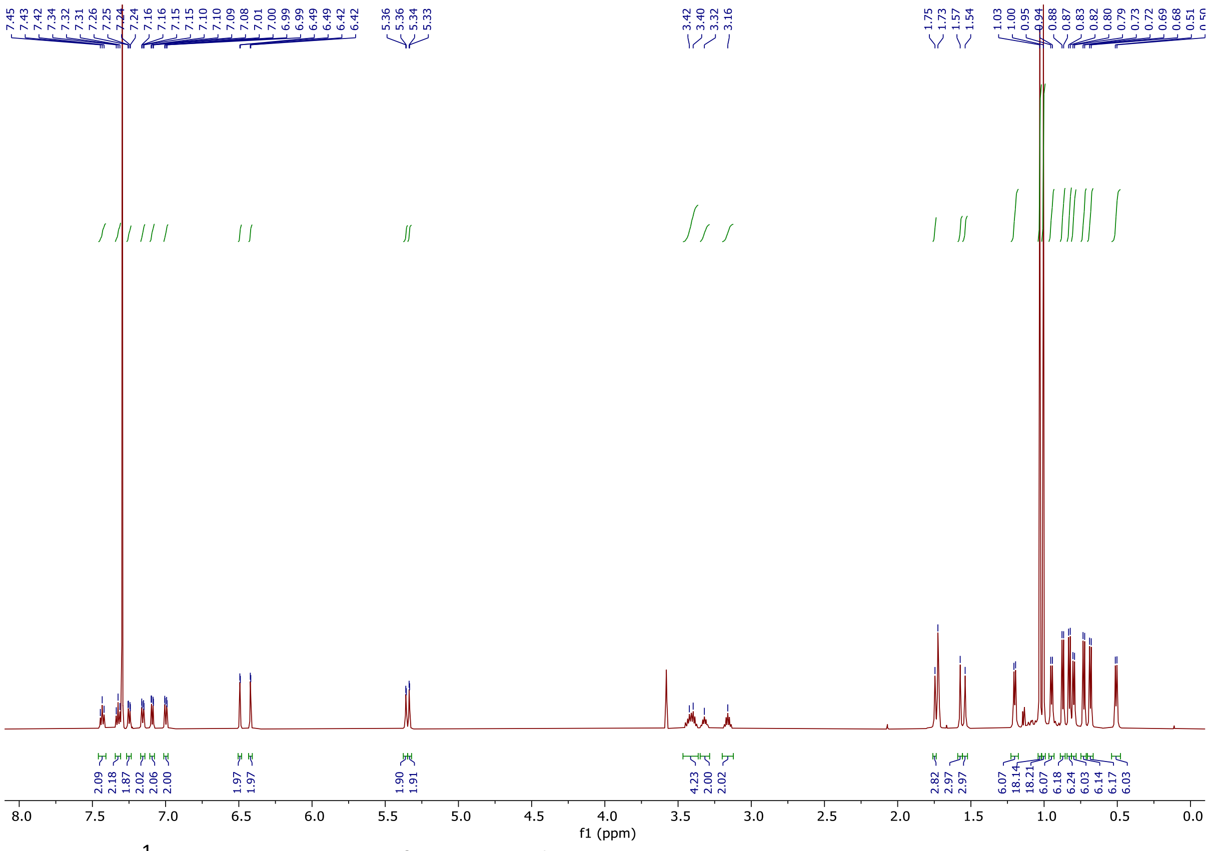

Figure S9: ^{1}H NMR spectrum of **3** in THF-d_8.

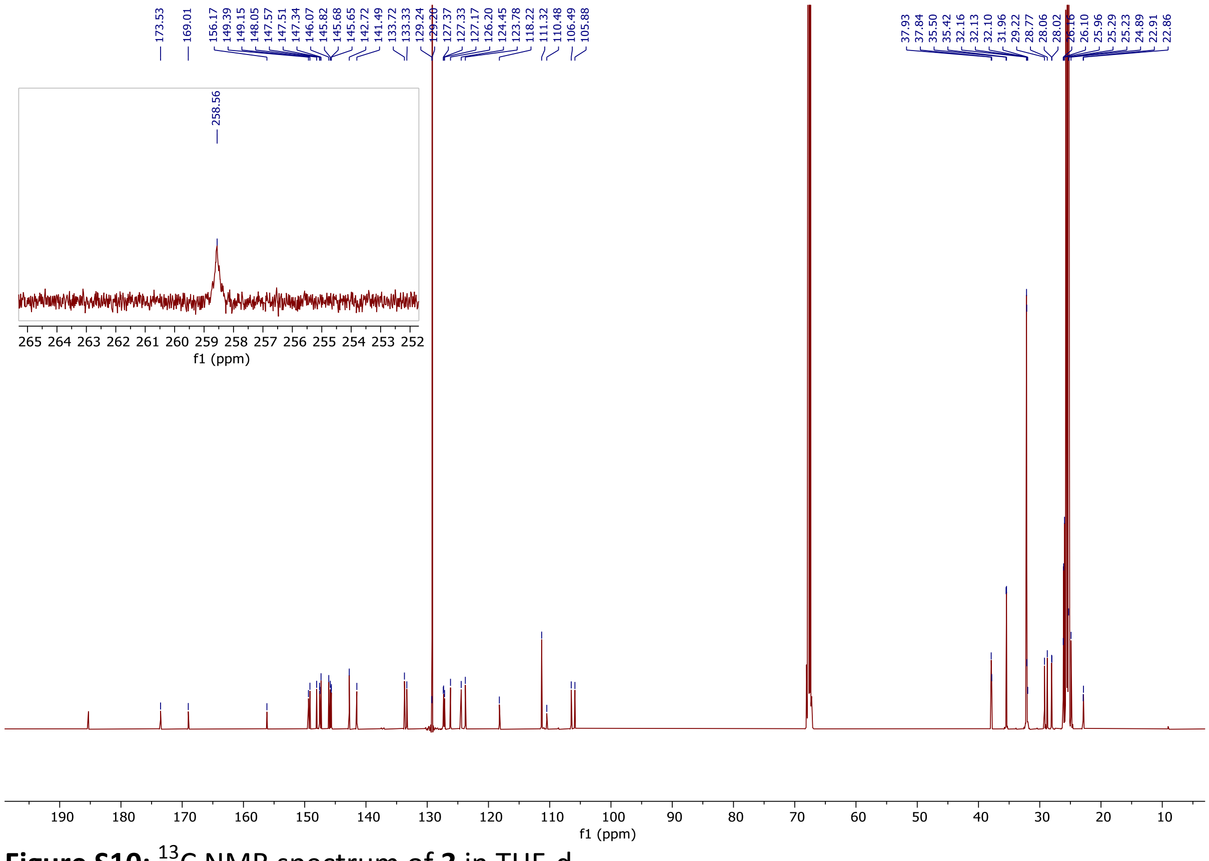

Figure S10: ^{13}C NMR spectrum of **3** in THF-d_8.

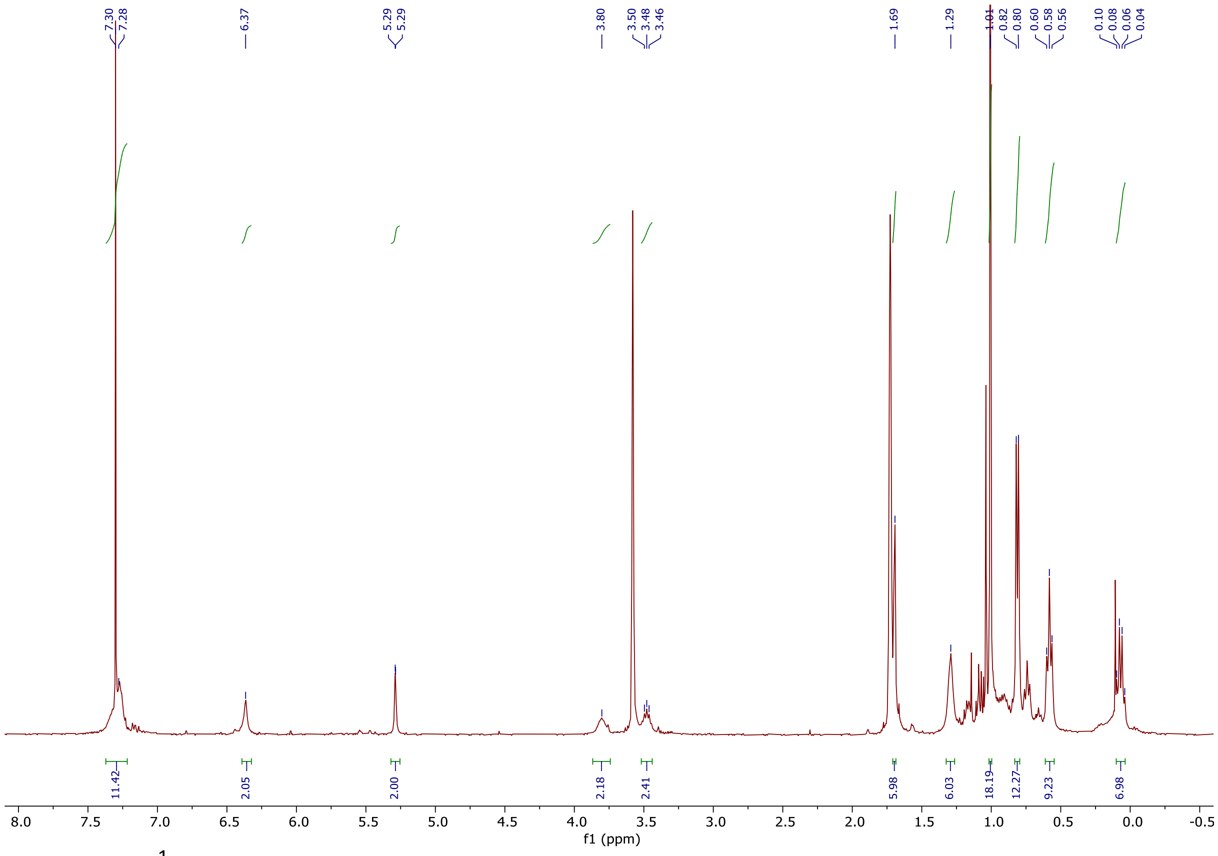

Figure S11: ^{1}H NMR spectrum of [**4**-$K_2(BEt_3)]_2$ in THF-d_8.

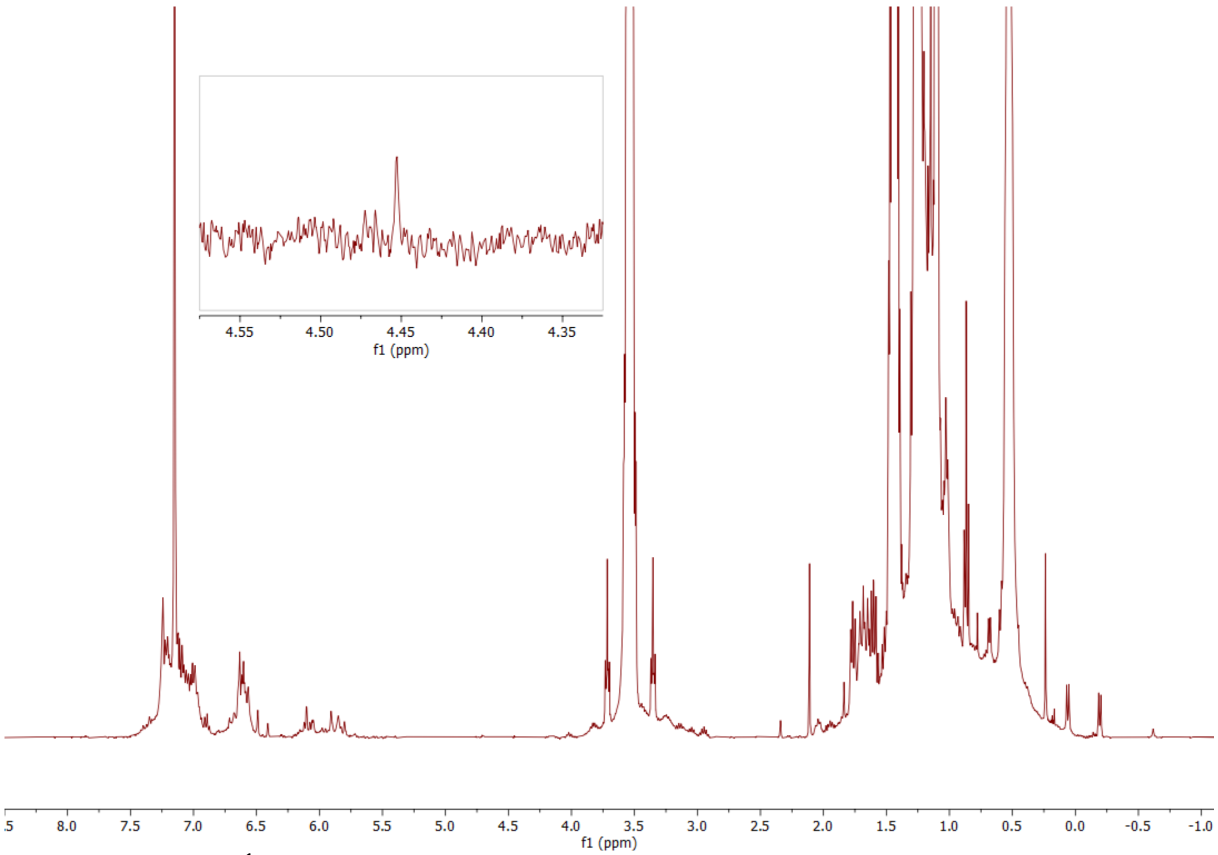

Figure S12: *In situ* ^{1}H NMR spectrum showing formation of [**4**-$K_2(BEt_3)]_2$ in THF-d_8.

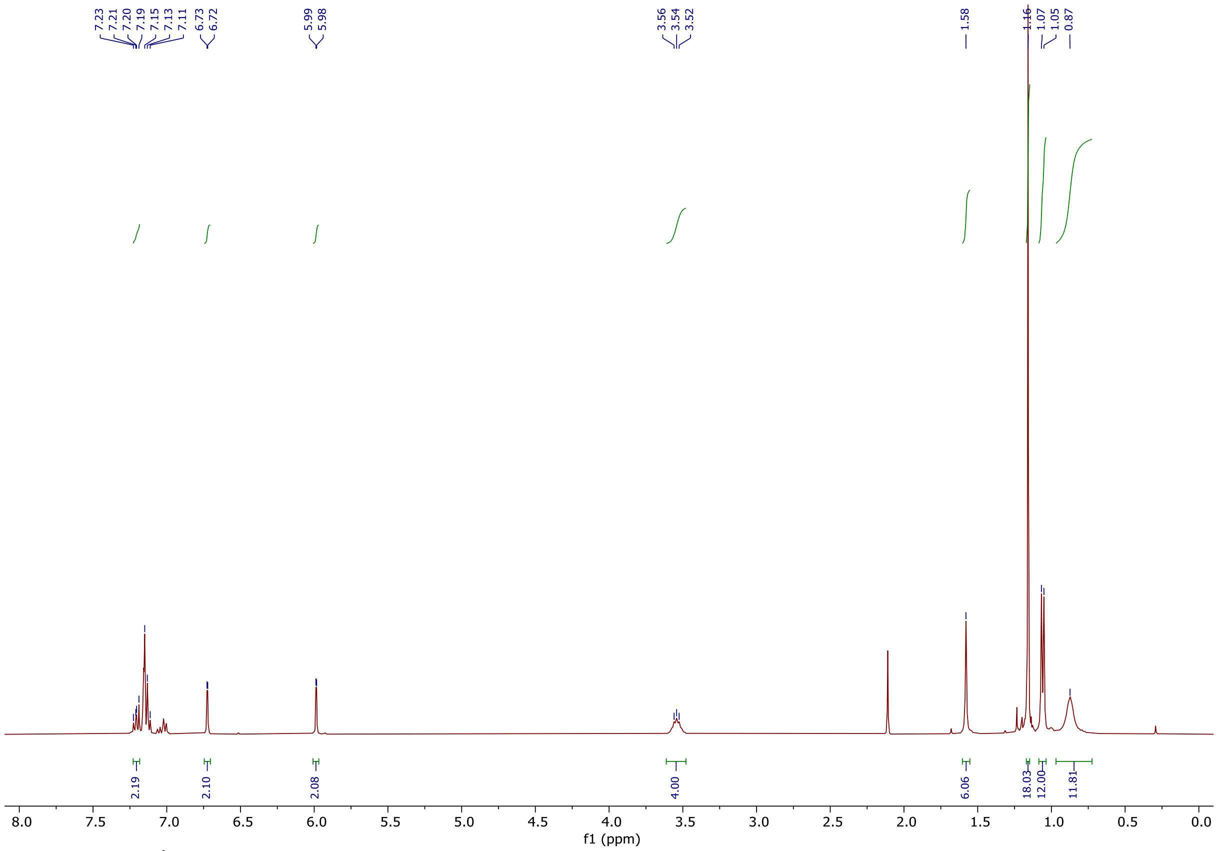

Figure S13: ^{1}H NMR spectrum of **5** in C_6D_6.

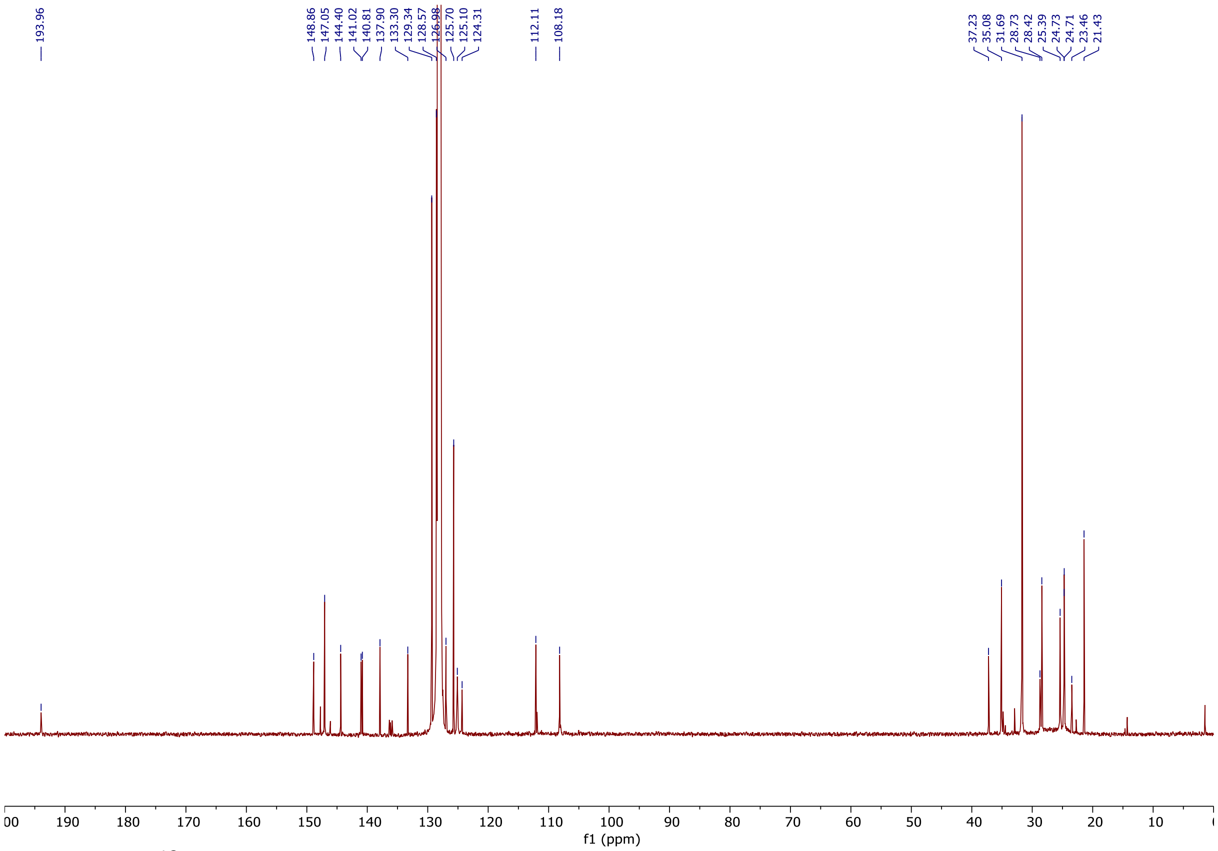

Figure S14: ^{13}C NMR spectrum of **5** in C_6D_6.

5 Crystallography:

Single-crystal X-ray diffraction data for all compounds were collected on an Oxford Diffraction/Agilent SuperNova diffractometer equipped with a 135 mm Atlas CCD area detector. Crystals were selected under Paratone-N oil, mounted on MiTeGen Micromount loops and quench-cooled using an Oxford Cryosystems open flow N2 cooling device.[S3] Data were collected at 150 K using mirror monochromated Cu Kα radiation (λ = 1.5418 Å; Oxford Diffraction Supernova). Data collected were processed using the CrysAlisPro package, including unit cell parameter refinement and inter-frame scaling (which was carried out using SCALE3 ABSPACK within CrysAlisPro).[S4] Equivalent reflections were merged and diffraction patterns processed with the CrysAlisPro suite.[S4]Structures were solved ab initio from the integrated intensities using SHELXT[S5] and refined on F2 using SHELXL[S6] with the graphical interface OLEX2[S7] or XSeed[S8]. Selected crystallographic data are summarised in Table S1, and full details are given in the supplementary deposited CIF files (CCDC 2114859-2114865 and 2118134). These data can be obtained free of charge from the Cambridge Crystallographic Data Centre via http://www.ccdc.cam.ac.uk/data_request/cif.

Compound	$[2\text{-}K(C_7H_8)]_2$	$[2\text{-}K(12\text{-}c\text{-}4)]_2$	$[2\text{-}Li(OEt_2)]_2$	$[2\text{-}Li(THF)]_2$
**Formula**	C140 H172 Al2 K2 N4 O6	C138 H180 Al2 K2 N4 O14	C118 H156 Al2 Li2 N4 O8	C118 H152 Al2 Li2 N4 O8
**Fw (g mol^{-1})**	2138.97	2251.01	1826.30	1822.27
**Cell setting**	triclinic	monoclinic	triclinic	monoclinic
**Space group**	P -1	P 2_1/n	P -1	P 2_1/c
**a (Å)**	11.9812(2)	14.9714(3)	12.9738(12)	21.0096(3)
**b (Å)**	14.1102(3)	24.1421(4)	13.1913(12)	19.3474(3)
**c (Å)**	18.7841(3)	18.3072(3)	17.1181(15)	26.6821(4)
**α (°)**	95.810(2)	90	70.273(8)	90
**β (°)**	96.5580(10)	103.748(2)	84.966(7)	98.5930(10)
**γ (°)**	97.410(2)	90	82.504(7)	90
**V (Å^3)**	3106.71(10)	6427.4(2)	2731.1(4)	10724.0(3)
**Z**	1	2	1	4
**ρ_{calc} (g cm^{-3})**	1.143	1.163	1.110	1.129
**Radiation, λ (Å)**	Cu K_α, 1.54184	Cu K_α, 1.54184	Cu K_α, 1.54184	Cu K_α, 1.54184
**μ (mm^{-1})**	1.238	1.267	0.670	0.682
**Reflections collected**	75174	46647	19852	88487
**Independent reflections**	12904	13283	9799	22269
**$R_{(int)}$**	0.026	0.0402	0.0592	0.0523
**Parameters**	808	936	673	1311
**R_1 (all data)**	0.0405	0.0760	0.1354	0.0686
**R_1 (I >2σ(I))**	0.0368	0.0569	0.0670	0.0452
**wR_2 (all data)**	0.1068	0.1684	0.1854	0.1231
**wR_2 (I >2σ(I))**	0.1029	0.1516	0.1500	0.1090
**GooF**	1.048	1.075	0.996	1.009
**T**	150(2)	150.06(15)	150.01(10)	150.01(10)
**CCDC ref**	2114863	2114865	2114859	2114862

Table S1: Crystallographic data for CO homologation and related compounds.

**Compound**	**$[2\text{-}K(THF)]_2$**	**3**	**$[4\text{-}K_2(BEt_3)]_2$**	**5**
**Formula**	C240 H336 Al4 K4 N8 O23	C240 H348 Al4 K4 N8 O26	C122 H166 Al2 B2 K4 N4 O6	C110.40 H149.60 Al2 N4 O6
**Fw (g mol^{-1})**	3965.47	4025.56	2016.56	1682.69
**Cell setting**	triclinic	monoclinic	triclinic	monoclinic
**Space group**	P -1	C 2/c	P -1	P 2_1/c
**a (Å)**	15.1682(5)	43.9642(13)	12.1088(5)	17.8436(4)
**b (Å)**	19.7249(6)	13.9217(3)	13.4265(6)	17.7254(2)
**c (Å)**	20.7358(4)	39.0993(10)	19.2028(7)	18.2264(5)
**α (°)**	79.886(2)	90	101.260(3)	90
**β (°)**	71.094(2)	102.525(3)	94.270(3)	118.887(3)
**γ (°)**	75.739(3)	90	113.177(4)	90
**V (Å^3)**	5657.3(3)	23361.5(11)	2774.8(2)	5047.5(2)
**Z**	1	4	1	2
**ρ_{calc} (g cm^{-3})**	1.164	1.145	1.207	1.107
**Radiation, λ (Å)**	Cu K_α, 1.54184	Cu K_α, 1.54184	Cu K_α, 1.54184	Cu K_α, 1.54184
**μ (mm^{-1})**	1.355	1.328	2.008	0.673
**Reflections collected**	92463	88466	42687	66822
**Independent reflections**	23286	24207	10348	10527
**$R_{(int)}$**	0.0671	0.0550	0.0836	0.0438
**Parameters**	1434	1332	650	585
**R_1 (all data)**	0.1000	0.0751	0.0853	0.0598
**R_1 (I >2σ(I))**	0.0700	0.0539	0.0527	0.0502
**wR_2 (all data)**	0.2231	0.1562	0.1467	0.1503
**wR2 (I >2σ(I))**	0.1936	0.1402	0.1256	0.1382
**GooF**	1.035	1.031	1.023	1.028
**T**	150.01(10)	150.00(10)	150.01(10)	150.01(10)
**CCDC ref**	2114861	2118134	2114860	2114864

Table S1/contd: Crystallographic data for CO homologation and related compounds.

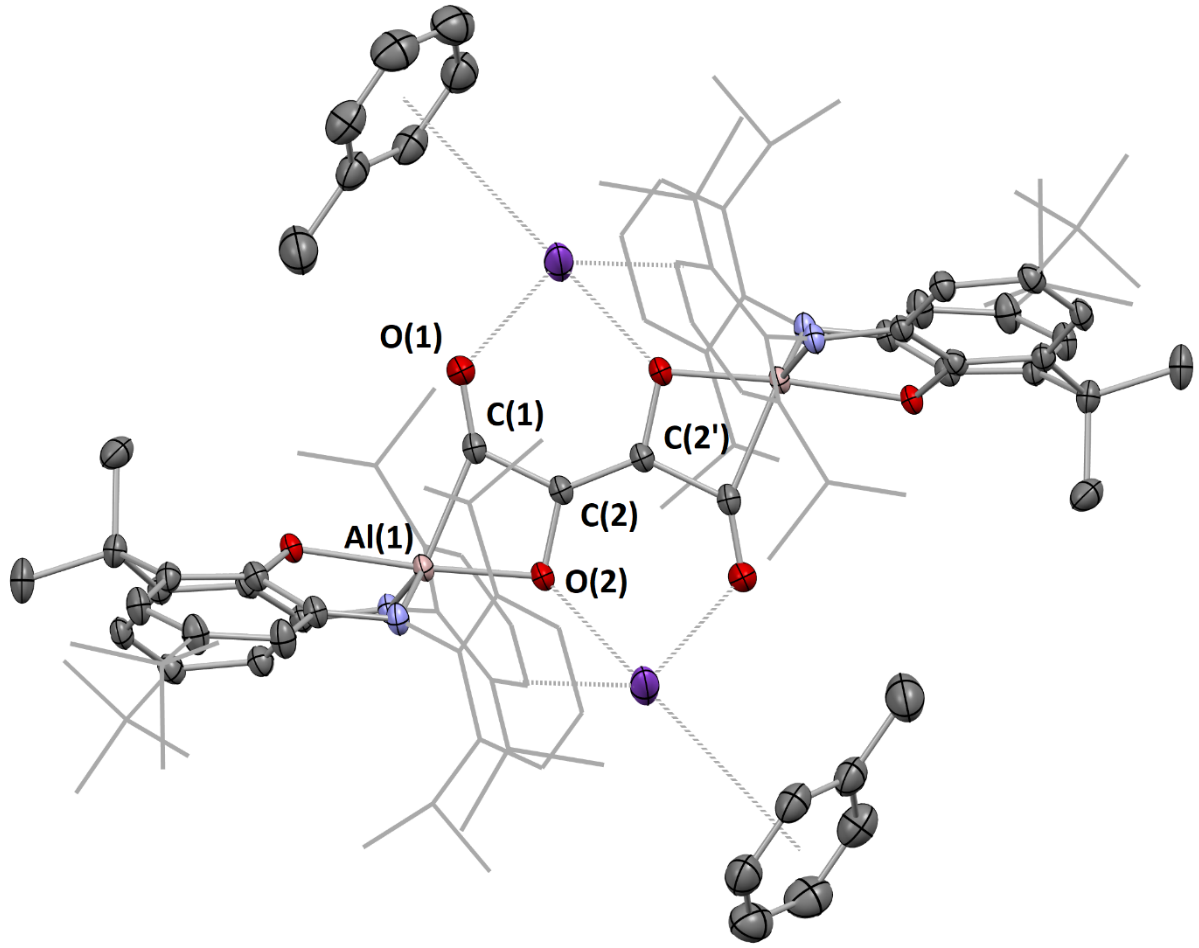

Figure S15: Molecular structure of [**2**-K(C_7H_8)]$_2$ in the solid state as determined by single crystal diffraction. Thermal ellipsoids have been set at 50% probability. Hydrogen atoms have been omitted and selected groups are shown in wireframe for clarity.

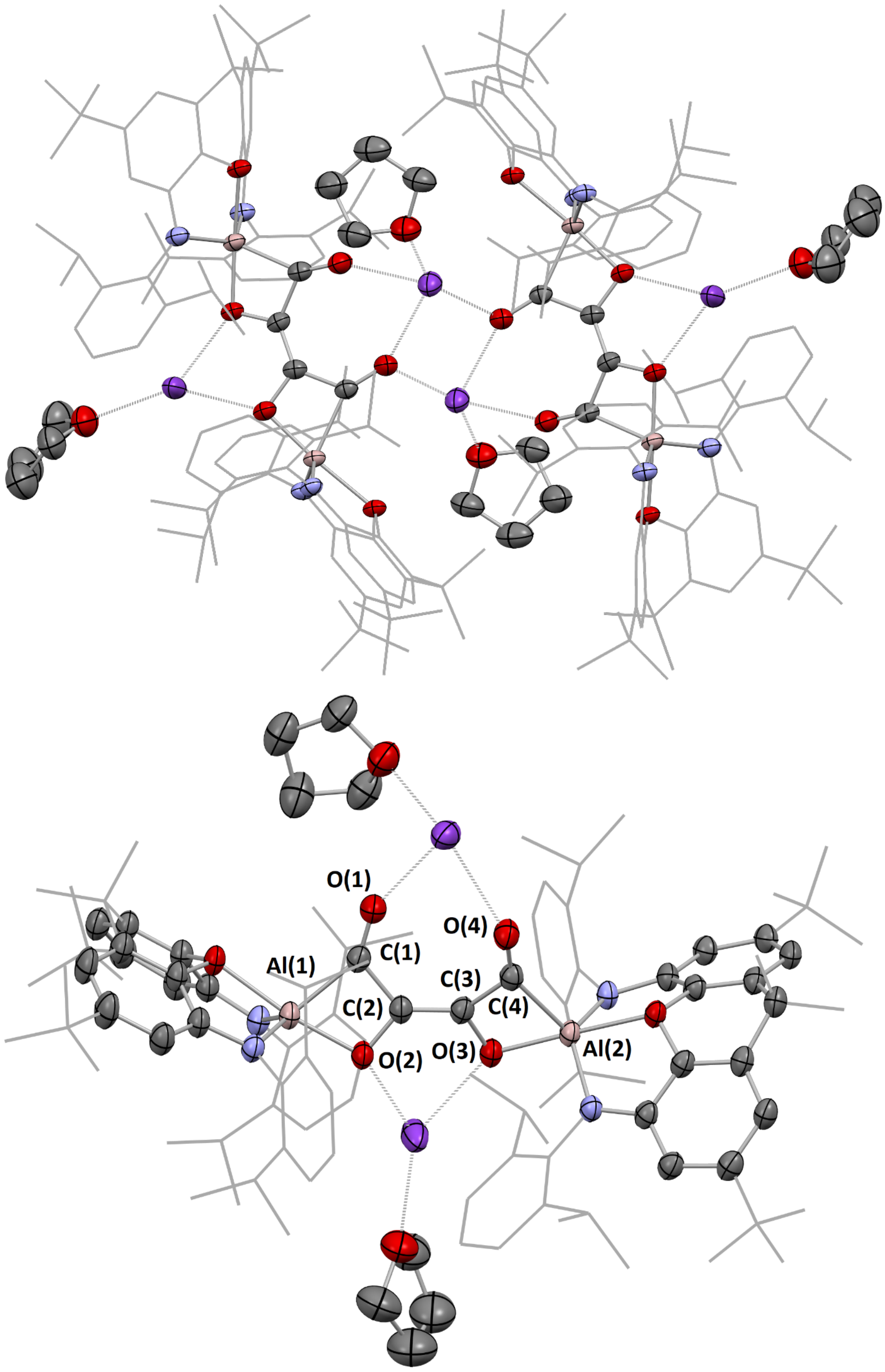

Figure S16: Molecular structure of [**2**-K(THF)]$_2$ (top) and the asymmetric unit (bottom) in the solid state as determined by single crystal diffraction. Thermal ellipsoids have been set at 50% probability. Hydrogen atoms have been omitted and selected groups are shown in wireframe for clarity.

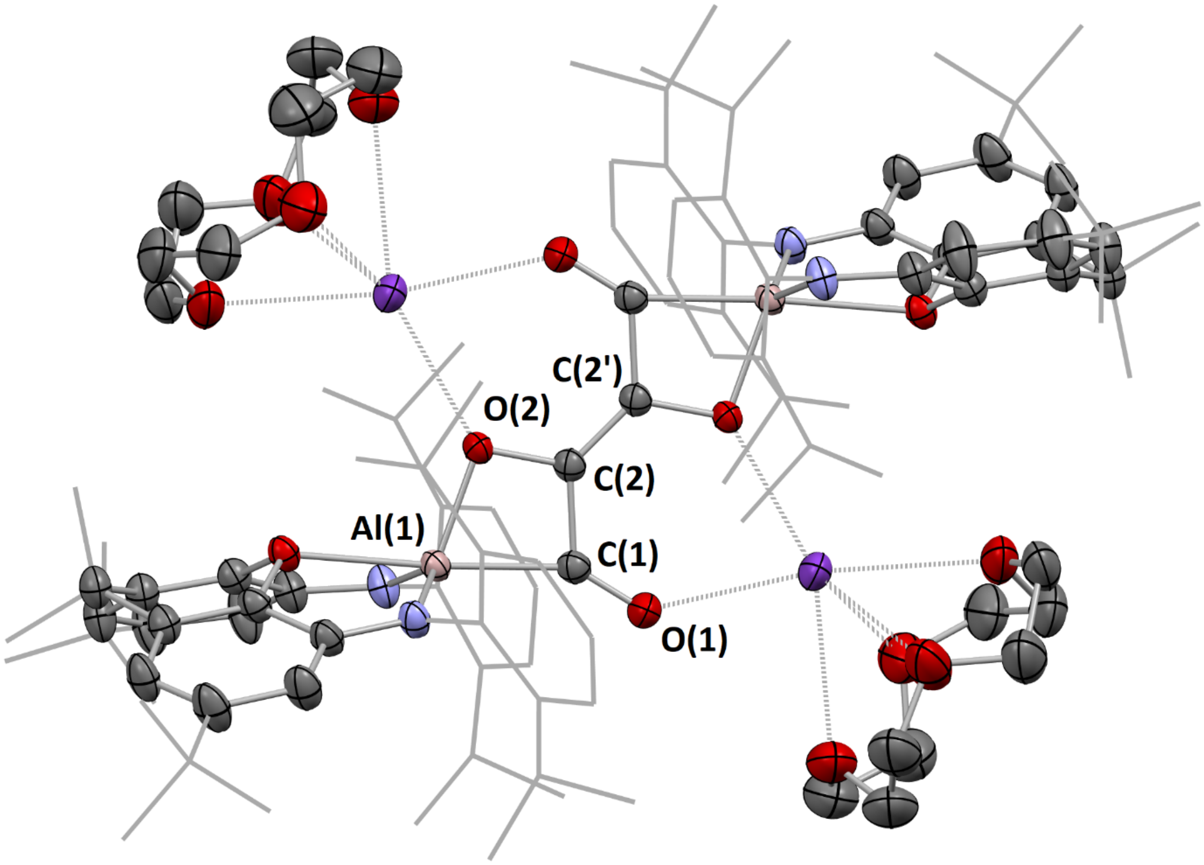

Figure S17: Molecular structure of [**2**-K(12-crown-4)]$_2$ in the solid state as determined by single crystal diffraction. Thermal ellipsoids have been set at 50% probability. Hydrogen atoms have been omitted and selected groups are shown in wireframe for clarity.

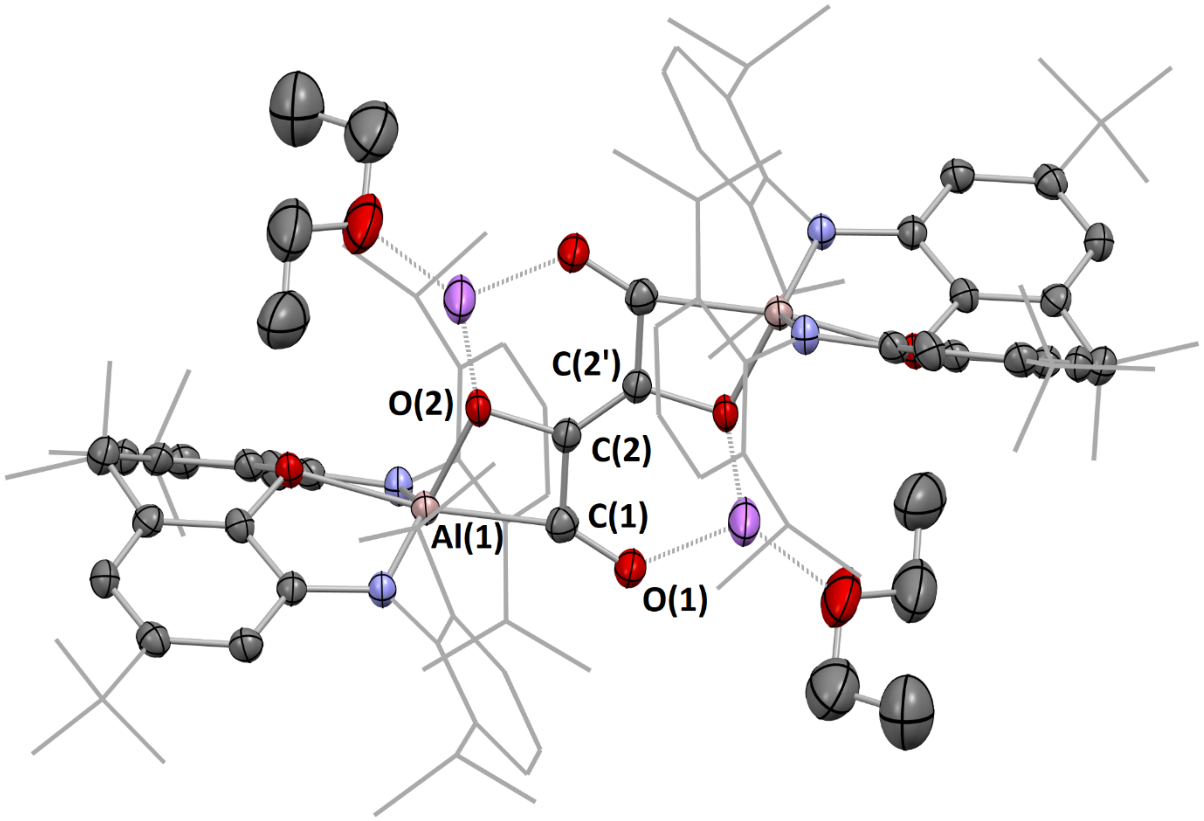

Figure S18: Molecular structure of [**2**-Li(OEt$_2$)]$_2$ in the solid state as determined by single crystal diffraction. Thermal ellipsoids have been set at 50% probability. Hydrogen atoms have been omitted and selected groups are shown in wireframe for clarity.

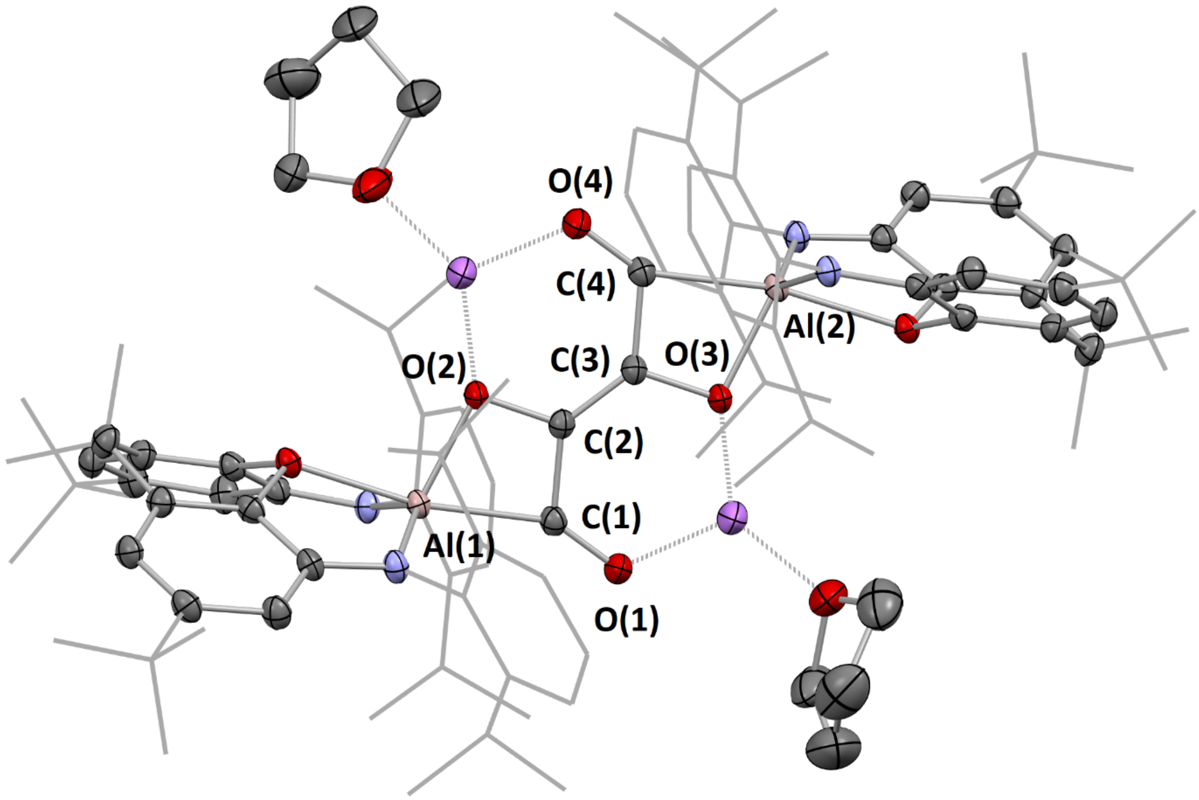

Figure S19: Molecular structure of [**2**-Li(THF)]$_2$ in the solid state as determined by single crystal diffraction. Thermal ellipsoids have been set at 50% probability. Hydrogen atoms have been omitted and selected groups are shown in wireframe for clarity.

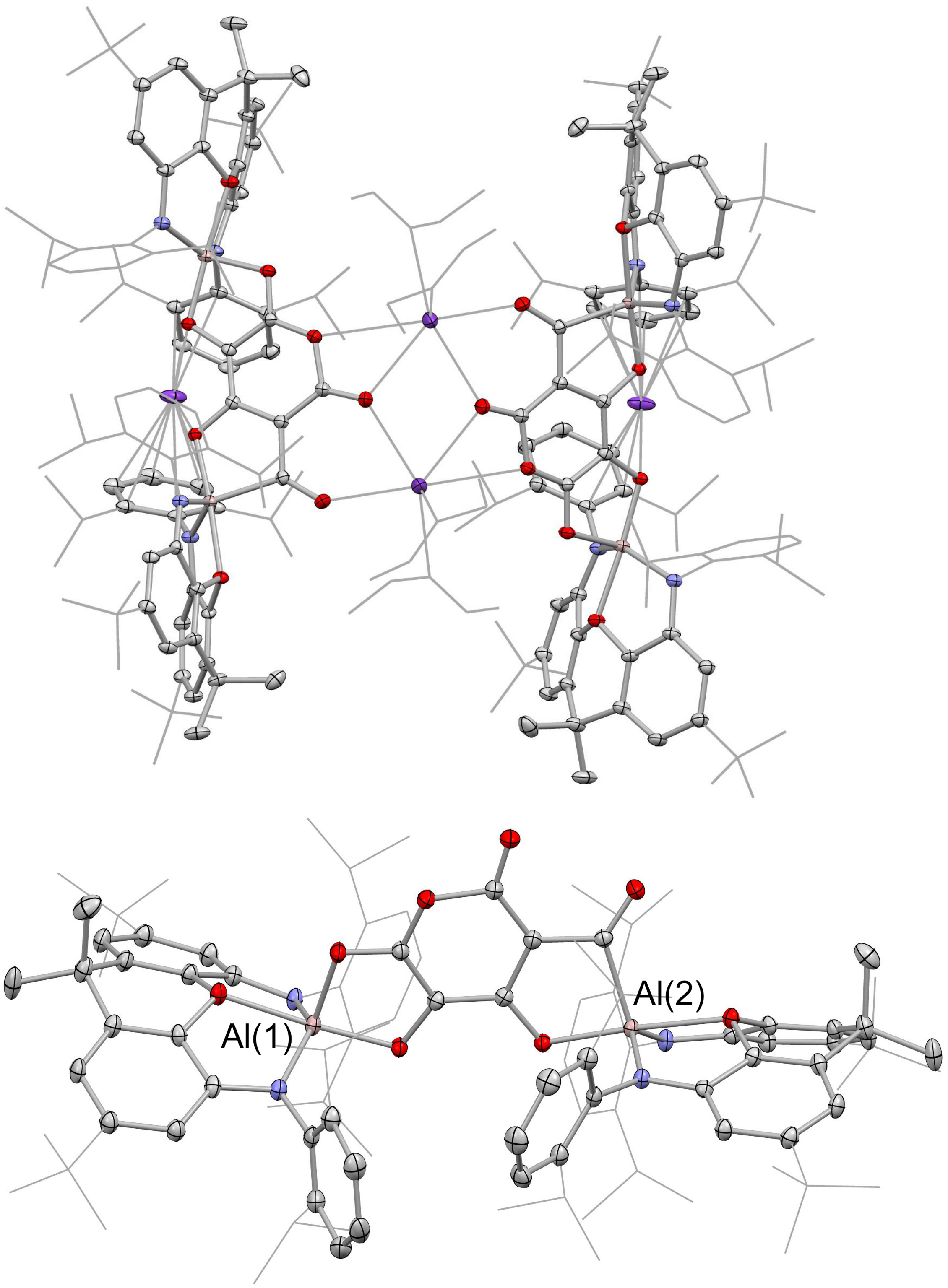

Figure S20: Molecular structure of **3** (top) and the anionic component of the asymmetric unit (bottom) in the solid state as determined by single crystal diffraction. Thermal ellipsoids have been set at 50% probability. Hydrogen atoms have been omitted and selected groups are shown in wireframe for clarity.

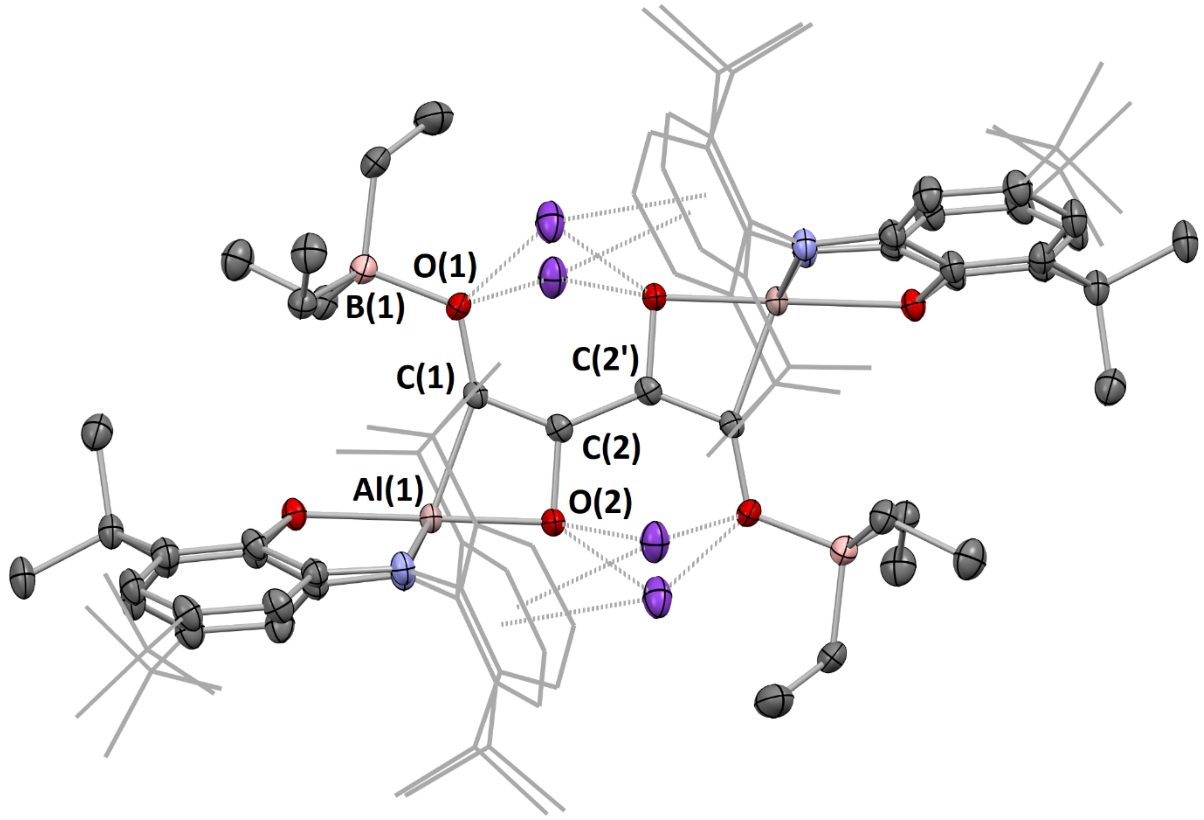

Figure S21: Molecular structure of [**4**-$K_2(BEt_3)$]$_2$ in the solid state as determined by single crystal diffraction. Thermal ellipsoids have been set at 50% probability. Hydrogen atoms have been omitted and selected groups are shown in wireframe for clarity.

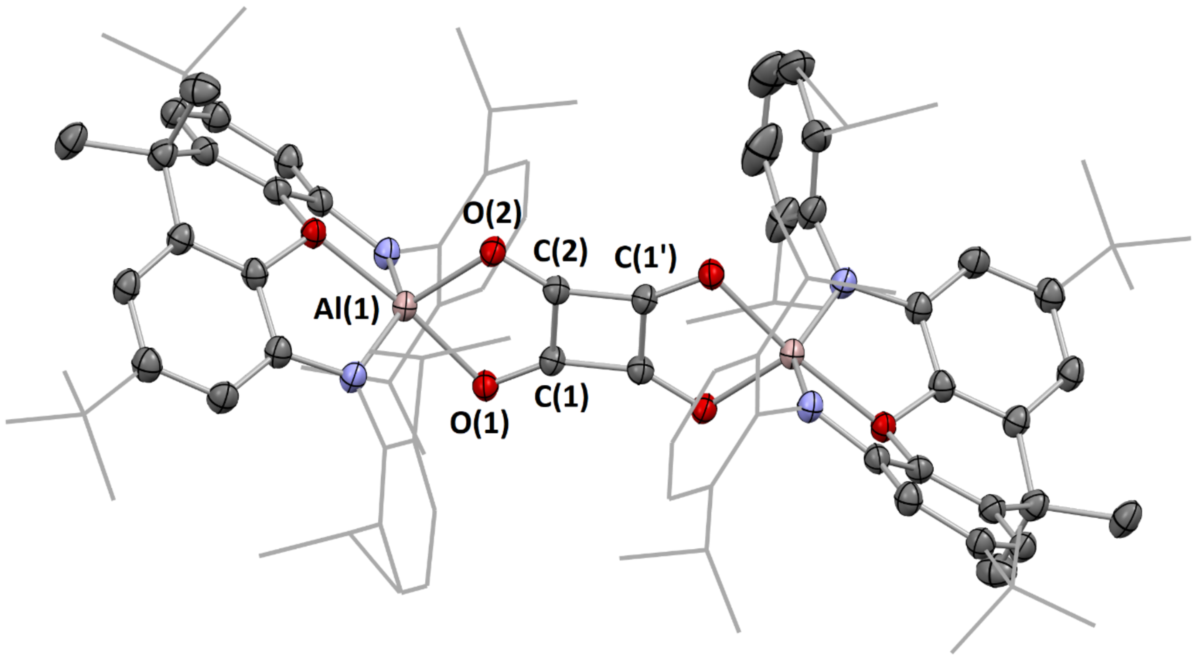

Figure S22: Molecular structure of **5** in the solid state as determined by single crystal diffraction. Thermal ellipsoids have been set at 50% probability. Hydrogen atoms have been omitted and selected groups are shown in wireframe for clarity.

6 Computational details:

All computational work reported here was carried out using density functional theory (DFT). Geometry optimisations for model compounds **A – F** and toluene were performed with Gaussian16 (Revision C.01)[S11] and for model compounds **G – J**, **R**, **P**, **TS1– TS4**, **I1 – I3** and carbon monoxide using Orca (Revision 5.0.2).[S12] Calculations were performed for model systems with simplified ligands to reduce computational cost. In all cases the tBu groups on the xanthene backbone were replaced by methyl substituents. Further, the Dipp groups were replaced with Ph groups for compounds **G – J**, or with ortho-Xylyl groups for compounds **R**, **P**, **TS1 – TS4**, **I1 – I3**.

Different isomers of the $[C_4O_4]^{2-}$ and $[C_4O_4]^{4-}$ containing compounds **A – E** were initially scoped using the B3LYP exchange correlation functional,[S13-S16] with Def-SVP basis sets,[S17,S18] and Grimme's empirical dispersion correction (GD3BJ).[S19,S20] Integration grid accuracy was set at ultrafine. The nature of stationary points found (minima) was confirmed by full frequency calculations. Further geometry optimizations were performed for synthetically viable species **A**, **E** and **F** using the B3LYP exchange correlation functional and Def-TZVP basis set, with Grimme's empirical dispersion correction (GD3BJ).

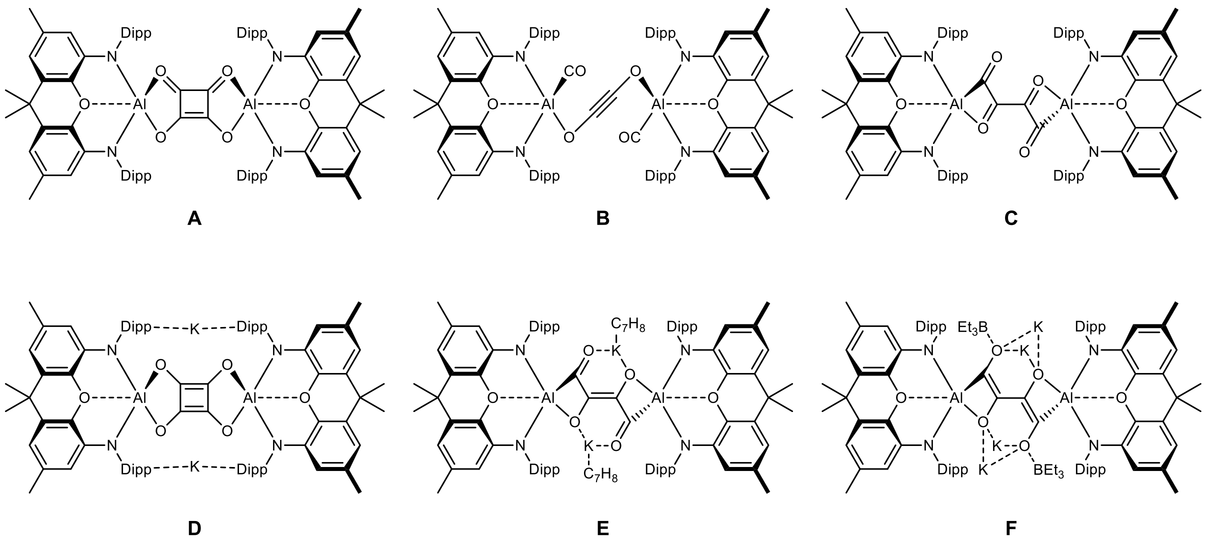

Figure S23: Model compounds **A** – **F**

	Enthalpy (Hartrees)	Relative to C/E (kJ mol^{-1})
**A**	-4488.762327	-335.933
**B**	-4488.651576	-45.156
**C**	-4488.634377	0
Toluene	-271.262744	
**D**	-5688.581626	
**D** + 2 Toluene	-6231.107114	213.0488
**E**	-6231.188260	0
**F**	-7412.923916	

Table S2: Sum of electronic and thermal enthalpies at 298.15 K and 1 atm calculated at the SVP level for model compounds **A** - **F**.

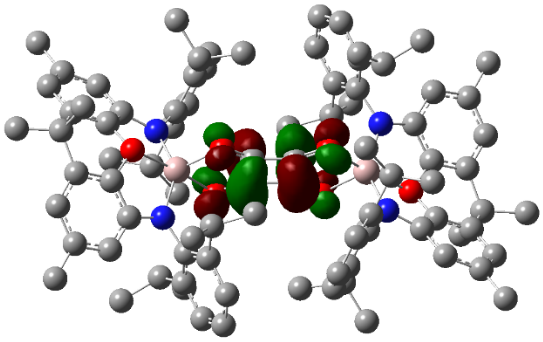

A, LUMO+1, -7.32767930E-02 Hartree

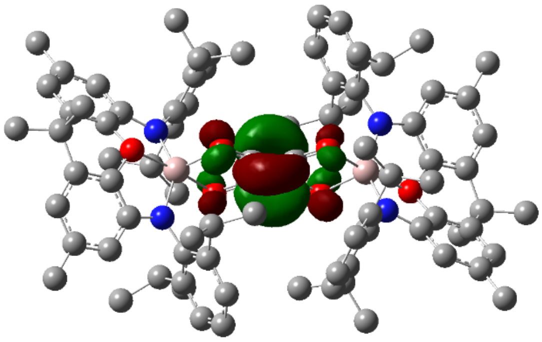

A, LUMO, -8.94303358E-02 Hartree

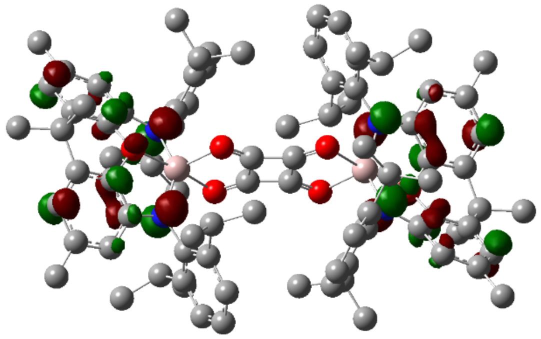

A, HOMO, -2.00482134E-01 Hartree

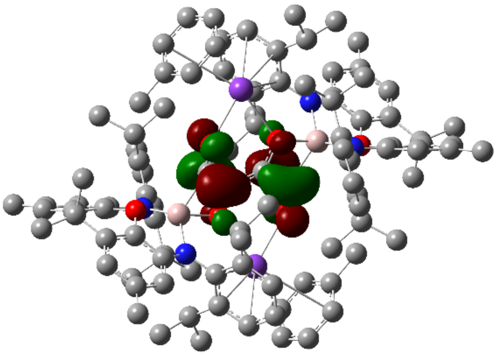

E, LUMO, -6.44873297E-02 Hartree

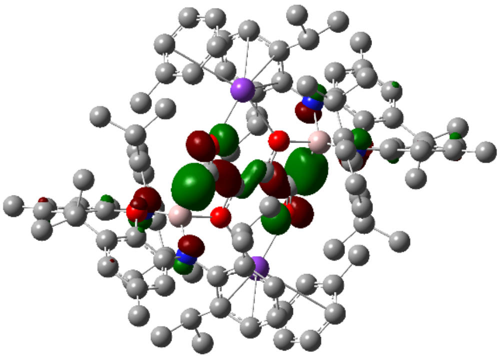

E, HOMO, -1.69247574E-01 Hartree

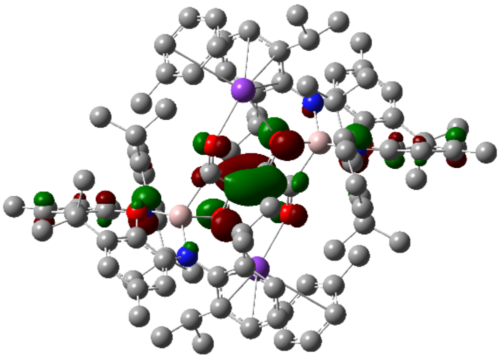

E, HOMO-1, -1.79567181E-01 Hartree

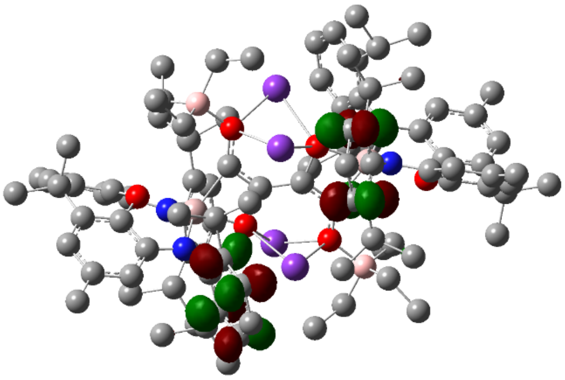

F, LUMO, -2.85128860E-02 Hartree

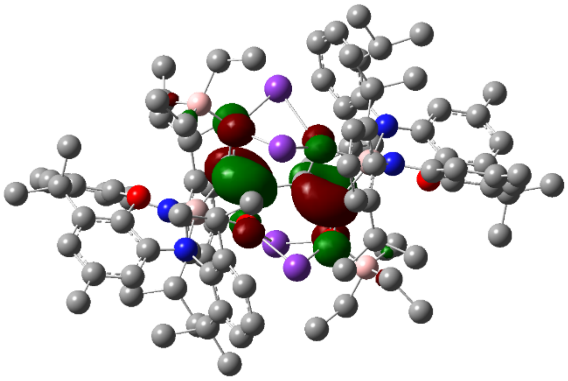

F, HOMO, -1.45382664E-01 Hartree

Figure S24: Selected frontier molecular orbitals for **A**, **E**, and **F** calculated at the TZVP level. (Iso-value 0.04)

The mechanism for the formation of **P** (**Figure S24**) is proposed to proceed through initial binding of CO in a bent fashion via the C atom (**I1**) with a second CO binding in reverse orientation via the O atom (**I2**). An isomerisation then occurs (**TS3**) to a carbene (**I3**) which can then dimerize to form **P**, the observed homologation product. Dimerization (**TS4**) is the rate determining step with a moderate high barrier due to the large steric bulk and electrostatic repulsion of the anionic fragments. This moderate high barrier is consistent with the experimental findings, that heating is required and low yields are observed.

Through the course of this mechanistic investigation a range of binding modes were considered (**Figure S25**), with the bent CO binding (**H**) being the lowest in energy for the initial binding of CO. Attempts to coordinate CO to the vacant p-orbital on Al in a linear fashion did not converge to a stable structure.

When binding two CO molecules, an energetic minimum was found featuring two Al bound C atoms (**I2′**). However, we propose that the isomeric system **I2** is the relevant intermediate in the transformation from **R** to **P** with **I2′** being an unproductive intermediate. The conversion between **I1** and **I2′** is somewhat reversible due to the moderate barrier and small ΔG, and as such the conversion of **I2** to **I2′** is proposed to proceed via **I1** not via direct rotational interconversion, as the barrier (**TS2B**) is significantly higher in energy. Binding of an isocarbonyl ligand OC to form **I2** is energetically unfavourable with respect to **I2′**; however onward isomerisation to carbene system **I3** is near barrier-less with **I3** lying downhill of both **R** and **I2′**. ETS-NOCV (using the implementation in multiwfn wavefunction analysis package)[S25] was used to further investigate the binding of the two CO molecules in **I2** and **I2′**. This analysis revealed that the binding of CO involves two main interactions; the dominant interaction involves the CO π* symmetry orbitals and the lone pair on the aluminium to form a multicentre bond (**Figure S26**). This bonding motif was also found to support reverse binding of CO in **I2** with only a modest decrease in the NOCV pair energy.

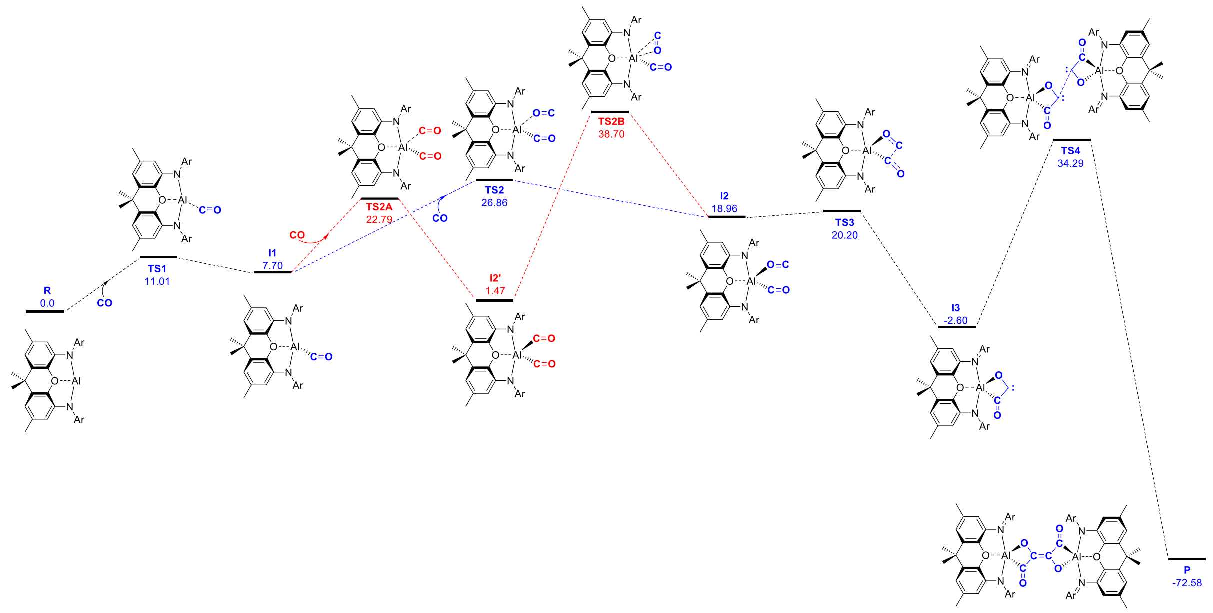

Figure S25: Proposed mechanism for CO homologation at B3LYP/def2-TZVP//B3LYP/def2-SVP with solvation modelled with CPCM(Benzene). Pathway in blue representing lowest energy pathway with reverse binding of CO (**I2**); pathway in red in a representing the prior binding of a second CO through carbon (**I2’**) then isomerization to **I2**

To investigate the mechanism of CO activation different binding modes of one or two CO molecules to the aluminium centre were calculated (**G – J**) using the using the B3LYP exchange correlation functional,[S12-S15] with 6-31G(d) Pople basis sets[S21,22] and DFT-D4 dispersion.[S23] Further mechanistic work (**R, P**, **TS1– TS4**, **I1 – I3**) was performed using the B3LYP exchange correlation functional,[S12-S15] with Def-SVP basis sets,[S16] DFT-D4 dispersion and LR-CPCM (benzene) solvent modelling,[S24] with single point energies being performed with the Def-TZVP basis set. The transition state structures (saddle points) were confirmed by having a single imaginary frequency.

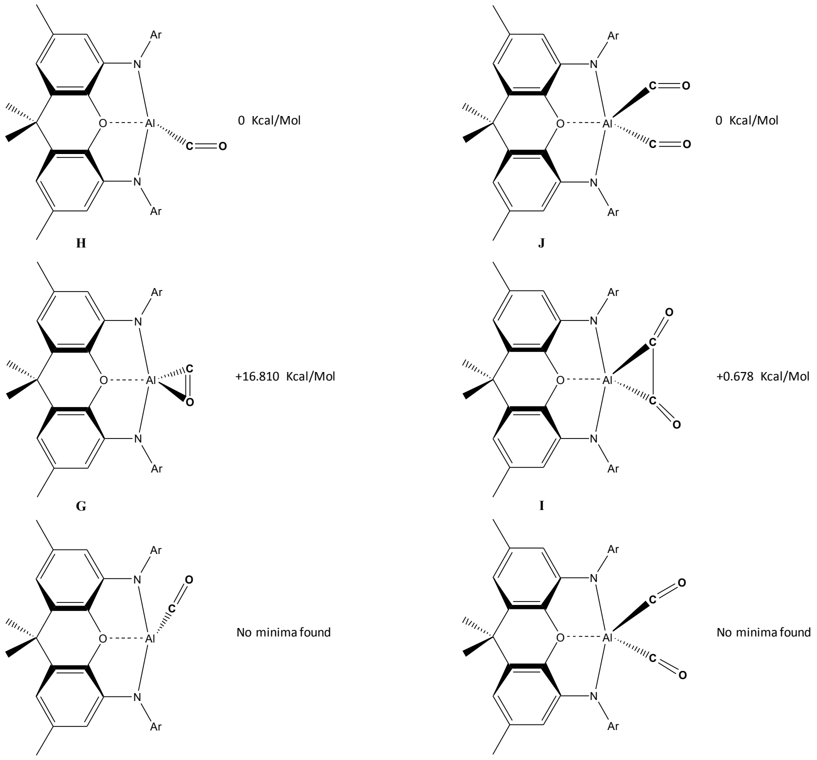

Figure S26: Different possible isomers of coordination of one and two CO molecules and their relative energies.

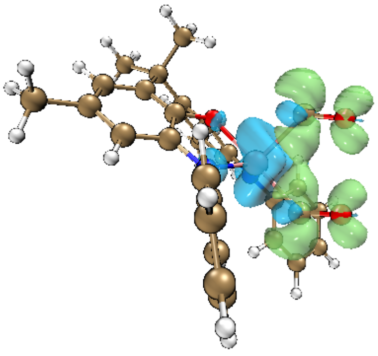

(**I2’**) ETS-NOCV pair 1, -4.1581833E-01 Hartree

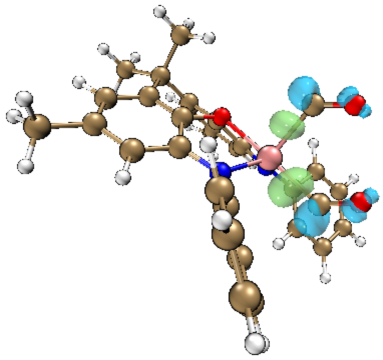

(**I2’**) ETS-NOCV pair 2, -4.014281E-02 Hartree

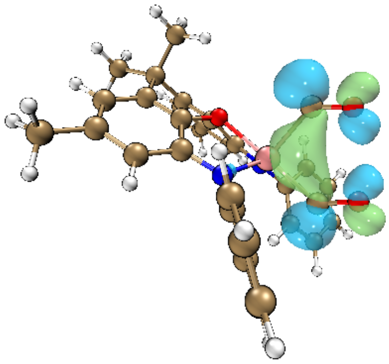

(**I2’**) HOMO, 5.8579E-01 Hartree

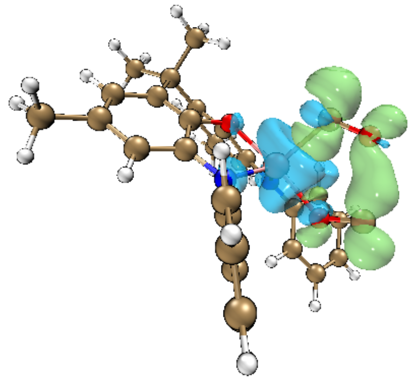

(**I2**) ETS-NOCV pair 1, -3.561061E-01 Hartree

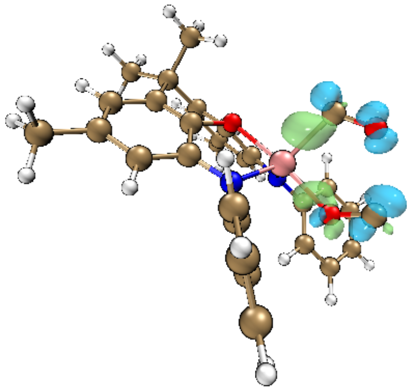

(**I2**) ETS-NOCV pair 2, -3.838985E-02 Hartree

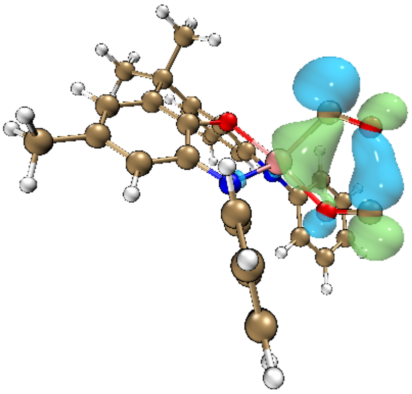

(**I2**) HOMO, 4.4697E-01 Hartree

Figure S27: Selected frontier molecular orbitals and ETS-NOCV pair densities for **I2** and **I2'** calculated at the 6-31G(d) level. (Iso-value 0.04 and 0.005 for Molecular orbitals and pair densities respectively)

xyz-Coordinates for optimized geometries

Gaussian outputs

A_SVP

```
198
SCF Done:  E(RB3LYP) =  -4489.00104413     A.U.
Al         3.23944        0.00005        0.23096
O          4.97777        0.00044       -0.82476
O          1.92181       -0.00018       -1.21798
O          1.69663       -0.00010        1.50880
N          3.77066        1.77475        0.51423
N          3.77148       -1.77437        0.51436
C          0.67148       -0.00003        0.78256
C          5.72446       -1.17118       -0.66999
C          5.72384        1.17249       -0.67024
C          5.03903       -2.14195        0.06433
C          0.78837       -0.00008       -0.66324
C          7.03447       -1.23770       -1.10615
C          7.69438        2.45586       -0.86031
H          8.72781        2.59179       -1.17977
C          5.03793        2.14300        0.06399
C          2.94655        2.87143        0.91570
C          7.61704        0.00104       -1.80868
C          2.78983        3.18375        2.28375
C          5.72147        3.35262        0.26770
H          5.23183        4.16473        0.80670
C          7.03967        3.50873       -0.19877
C          7.69569       -2.45353       -0.85979
H          8.72922       -2.58893       -1.17915
C          7.03379        1.23962       -1.10642
C          5.72322       -3.35117        0.26827
H          5.23397       -4.16346        0.80735
C          7.04152       -3.50665       -0.19812
C          2.39066        3.70075       -0.09008
C          2.94777       -2.87159        0.91522
C          1.73365        4.87453        0.29847
H          1.30818        5.52757       -0.46573
C          7.74412        4.82593        0.01939
C          3.36326        2.28595        3.36866
H          3.68239        1.35483        2.88093
C          1.62699        5.22411        1.64349
H          1.12947        6.15429        1.92960
C          2.79077       -3.18444        2.28311
C          2.52635        3.35901       -1.56832
H          3.03686        2.39134       -1.63819
C          9.14804        0.00147       -1.77351
H          9.52788        0.00170       -0.74137
H          9.54694        0.88521       -2.29175
H          9.54743       -0.88217       -2.29153
C          3.36317       -2.28656        3.36850
H          3.68198       -1.35514        2.88113
C          2.13389        4.37406        2.62519
H          2.02323        4.64273        3.67811
C          7.13710        0.00073       -3.28208
H          7.50881       -0.89468       -3.80392
H          7.50832        0.89623       -3.80414
H          6.03900        0.00043       -3.34048
C          7.74659       -4.82354        0.01989
C          2.39267       -3.70089       -0.09101
C          1.15879        3.20438       -2.24504
H          0.48813        2.54246       -1.67714
H          1.27105        2.78684       -3.25817
H          0.64179        4.16816       -2.33583
C          2.13548       -4.37527        2.62395
H          2.02466       -4.64436        3.67675
C          2.31158        1.91649        4.42199
H          1.96004        2.80018        4.97852
H          2.73312        1.21158        5.15560
H          1.43995        1.43734        3.95249
C          3.39199        4.38600       -2.31503
H          2.92776        5.38575       -2.29719
H          3.51333        4.09302       -3.37054
H          4.39233        4.46505       -1.86553
C          2.52861       -3.35857       -1.56909
H          3.03847       -2.39053       -1.63846
C          4.61225        2.91462        4.00551
H          5.38114        3.12130        3.24572
H          5.04914        2.23898        4.75897
H          4.36677        3.86558        4.50663
C          4.61220       -2.91462        4.00586
H          4.36704       -3.86584        4.50665
H          5.04829       -2.23889        4.75970
H          5.38161       -3.12067        3.24643
C          1.73623       -4.87519        0.29694
H          1.31138       -5.52822       -0.46762
C          1.62939       -5.22530        1.64182
H          1.13234       -6.15588        1.92746
C          2.31072       -1.91794        4.42137
H          1.43902       -1.43927        3.95152
H          2.73143       -1.21289        5.15533
H          1.95945       -2.80196        4.97756
C          1.16119       -3.20461       -2.24625
H          0.64499       -4.16876       -2.33782
H          1.27354       -2.78641       -3.25910
H          0.48978       -2.54354       -1.67823
C          3.39523       -4.38470       -2.31584
H          4.39548       -4.46318       -1.86603
H          3.51671       -4.09131       -3.37122
H          2.93171       -5.38478       -2.29845
O         -1.92180        0.00019        1.21799
O         -1.69662        0.00013       -1.50878
C         -0.67146        0.00007       -0.78254
C         -0.78836        0.00012        0.66325
Al        -3.23943       -0.00005       -0.23095
O         -4.97777       -0.00046        0.82477
N         -3.77065       -1.77474       -0.51428
N         -3.77147        1.77438       -0.51433
C         -5.72445        1.17117        0.67002
C         -5.72383       -1.17250        0.67019
C         -5.03902        2.14195       -0.06429
C         -7.03446        1.23768        1.10617
C         -7.69438       -2.45587        0.86022
H         -8.72782       -2.59179        1.17966
C         -5.03793       -2.14299       -0.06406
C         -2.94654       -2.87143       -0.91572
C         -7.61703       -0.00108        1.80867
C         -2.78984       -3.18379       -2.28376
C         -5.72148       -3.35260       -0.26781
H         -5.23183       -4.16470       -0.80682
C         -7.03968       -3.50872        0.19865
C         -7.69568        2.45351        0.85983
H         -8.72921        2.58891        1.17919
C         -7.03379       -1.23964        1.10637
C         -5.72321        3.35117       -0.26821
H         -5.23396        4.16347       -0.80728
C         -7.04151        3.50665        0.19817
C         -2.39065       -3.70072        0.09007
C         -2.94777        2.87161       -0.91519
C         -1.73366       -4.87452       -0.29843
H         -1.30820       -5.52754        0.46578
C         -7.74414       -4.82591       -0.01955
C         -3.36328       -2.28603       -3.36870
H         -3.68252       -1.35494       -2.88098
C         -1.62702       -5.22414       -1.64345
H         -1.12952       -6.15434       -1.92953
C         -2.79078        3.18447       -2.28308
C         -2.52635       -3.35893        1.56830
H         -3.03683       -2.39124        1.63813
C         -9.14803       -0.00150        1.77351
H         -9.52787       -0.00170        0.74137
H         -9.54694       -0.88525        2.29172
H         -9.54742        0.88213        2.29155
C         -3.36316        2.28659       -3.36848
H         -3.68198        1.35517       -2.88111
C         -2.13392       -4.37412       -2.62517
H         -2.02327       -4.64283       -3.67809
C         -7.13708       -0.00082        3.28206
H         -7.50879        0.89459        3.80394
H         -7.50830       -0.89633        3.80410
H         -6.03898       -0.00052        3.34046
C         -7.74658        4.82353       -0.01983
C         -2.39267        3.70090        0.09105
C         -1.15880       -3.20432        2.24504
H         -0.48810       -2.54244        1.67713
H         -1.27106       -2.78674        3.25816
H         -0.64183       -4.16811        2.33588
C         -2.13551        4.37532       -2.62390
H         -2.02470        4.64442       -3.67670
C         -2.31157       -1.91646       -4.42196
H         -1.95989       -2.80012       -4.97846
H         -2.73312       -1.21159       -5.15560
H         -1.44002       -1.43723       -3.95239
C         -3.39205       -4.38586        2.31503
H         -2.92786       -5.38562        2.29722
H         -3.51340       -4.09285        3.37052
H         -4.39239       -4.46489        1.86550
C         -2.52862        3.35854        1.56913
H         -3.03846        2.39049        1.63847
C         -4.61219       -2.91479       -4.00564
H         -5.38109       -3.12156       -3.24590
H         -5.04909       -2.23917       -4.75910
H         -4.36660       -3.86572       -4.50677
C         -4.61218        2.91464       -4.00586
H         -4.36701        3.86586       -4.50665
H         -5.04825        2.23890       -4.75970
H         -5.38160        3.12069       -3.24645
C         -1.73625        4.87521       -0.29688
H         -1.31141        5.52823        0.46768
C         -1.62943        5.22534       -1.64176
H         -1.13240        6.15593       -1.92739
```

C	-2.31069	1.91796	-4.42133
H	-1.43899	1.43929	-3.95145
H	-2.73139	1.21291	-5.15528
H	-1.95941	2.80198	-4.97751
C	-1.16120	3.20459	2.24629
H	-0.64502	4.16874	2.33790
H	-1.27355	2.78635	3.25913
H	-0.48977	2.54354	1.67826
C	-3.39526	4.38465	2.31589
H	-4.39551	4.46313	1.86607
H	-3.51675	4.09123	3.37126
H	-2.93175	5.38474	2.29853
H	-8.82031	-4.75053	0.19416
H	-7.62505	-5.17771	-1.05666
H	-7.32727	-5.60986	0.63567
H	-8.82321	4.74690	0.19116
H	-7.33206	5.60684	0.63768
H	-7.62522	5.17706	-1.05606
H	7.33211	-5.60683	-0.63767
H	8.82323	-4.74689	-0.19102
H	7.62517	-5.17709	1.05611
H	7.62506	5.17775	1.05649
H	8.82029	4.75055	-0.19434
H	7.32723	5.60986	-0.63584

B_SVP

198

SCF Done: E(RB3LYP) = -4490.40613834 A.U.

C	-1.99597	-0.78521	-1.51878
O	-1.66928	-1.18788	-2.52350
C	-0.48912	0.30616	0.37209
C	0.48442	0.29932	-0.37040
C	2.00065	-0.79066	1.52452
O	-1.58330	0.32821	1.05441
O	1.57774	0.30787	-1.05459
O	1.67492	-1.19441	2.52887
Al	3.13008	-0.03742	-0.21812
N	3.71584	-1.53186	-1.19200
N	3.63353	1.67577	0.36551
C	5.00521	-1.99882	-0.95351
C	2.84316	-2.38245	-1.93971
C	4.84648	1.81919	1.03256
C	2.96816	2.89653	0.02432
C	5.65513	-1.42865	0.14665
C	5.74211	-2.95072	-1.67389
C	2.44460	-3.61996	-1.37450
C	2.37892	-1.98994	-3.21715
C	5.54791	0.63203	1.27282
C	5.48930	3.00035	1.43949
C	2.04604	3.47305	0.92890
C	3.29891	3.54824	-1.18632
O	4.85819	-0.49149	0.80965
C	6.96119	-1.64930	0.54039
C	7.06767	-3.24813	-1.30682
H	5.28345	-3.44867	-2.52966
C	1.62203	-4.46900	-2.12552
C	2.85502	-4.04259	0.03206
C	1.53228	-2.86009	-3.91644
C	2.83224	-0.67869	-3.84292
C	6.84637	0.52290	1.73475
H	4.99459	3.96064	1.28999
C	6.78387	2.95196	1.98776
C	1.62460	2.75550	2.20255
C	1.53163	4.74273	0.63954
C	2.75820	4.81673	-1.43448
C	4.23039	2.91066	-2.20720
C	7.47807	-0.87981	1.76930
C	7.67320	-2.59340	-0.22095
C	7.83061	-4.28677	-2.09274
H	1.31863	-5.43015	-1.70514
C	1.16498	-4.09635	-3.38631
H	3.44264	-3.22581	0.47086
C	3.74994	-5.29096	0.02642
C	1.63020	-4.25602	0.93247
H	1.16088	-2.57371	-4.90080
H	2.94089	0.05017	-3.03020
C	1.81310	-0.09252	-4.82385
C	4.21185	-0.84502	-4.50037
C	7.46326	1.72863	2.11248
C	7.44127	4.23721	2.42845
H	1.82653	1.68806	2.03944
C	0.12669	2.90109	2.49617
C	2.46504	3.19823	3.41144
H	0.82404	5.20211	1.32943
C	1.89881	5.42137	-0.52039
H	3.01575	5.33987	-2.35769
H	4.31841	1.84393	-1.95015
C	3.66093	2.98855	-3.62891
C	5.64580	3.50607	-2.14183
C	9.00787	-0.80850	1.78640
C	6.98475	-1.61153	3.04309
H	8.70887	-2.82670	0.02703
H	7.77331	-4.09177	-3.17561
H	8.89211	-4.31327	-1.80681
H	7.41288	-5.29430	-1.92548
H	0.51367	-4.76492	-3.95447
H	3.21429	-6.16014	-0.38892
H	4.06064	-5.54891	1.05190
H	4.65667	-5.12887	-0.57290
H	0.93762	-3.40362	0.90030
H	1.93406	-4.40546	1.98089
H	1.05812	-5.14467	0.62688
H	1.71349	-0.70345	-5.73538
H	2.12773	0.91200	-5.14222
H	0.82006	-0.00828	-4.35659
H	4.96232	-1.18413	-3.77170
H	4.55938	0.10912	-4.92852
H	4.16890	-1.58735	-5.31436
H	8.48279	1.72532	2.49819
H	7.35105	5.01765	1.65606
H	6.96447	4.63233	3.34161
H	8.50972	4.09283	2.64531
H	-0.12939	3.92101	2.82234
H	-0.16828	2.21518	3.30534
H	-0.48697	2.66743	1.61730
H	3.53774	3.02231	3.25051
H	2.15836	2.65025	4.31756
H	2.32381	4.27460	3.60496
H	1.49412	6.41610	-0.72356
H	2.63393	2.59906	-3.66311
H	4.27922	2.39767	-4.32229
H	3.64404	4.02313	-4.00663
H	5.62553	4.58258	-2.37981
H	6.31018	3.01093	-2.86872
H	6.08432	3.38825	-1.14097
H	9.39731	-0.29504	0.89522
H	9.44212	-1.81775	1.82471
H	9.36112	-0.27292	2.67910
H	7.30903	-1.06923	3.94472
H	7.39100	-2.63427	3.07785
H	5.88712	-1.67838	3.06042
Al	-3.13294	-0.03546	0.22048
N	-3.70444	-1.53060	1.20126
N	-3.65471	1.67049	-0.36729
C	-4.98564	-2.01701	0.95815
C	-2.82298	-2.36287	1.95909
C	-4.86669	1.79796	-1.03911
C	-2.99810	2.89826	-0.03527
C	-5.63946	-1.45771	-0.14541
C	-5.71202	-2.97803	1.67712
C	-2.39891	-3.59536	1.40222
C	-2.37303	-1.95542	3.23714
C	-5.55409	0.60193	-1.27648
C	-5.52108	2.96990	-1.45397
C	-2.08296	3.47608	-0.94600
C	-3.32856	3.55387	1.17342
O	-4.85259	-0.51175	-0.80764
C	-6.94126	-1.69568	-0.54293
C	-7.03225	-3.29367	1.30587
H	-5.24984	-3.46835	2.53540
C	-1.56525	-4.42583	2.16193
C	-2.79231	-4.03064	-0.00518
C	-1.51502	-2.80683	3.94549
C	-2.85090	-0.64694	3.85070
C	-6.84951	0.47580	-1.74226
H	-5.03758	3.93635	-1.30738
C	-6.81338	2.90436	-2.00604
C	-1.66043	2.75459	-2.21695
C	-1.57621	4.75082	-0.66553
C	-2.79487	4.82694	1.41311
C	-4.25264	2.91475	2.20011
C	-7.46456	-0.93449	-1.77443
C	-7.64313	-2.64819	0.21747
C	-7.78377	-4.34163	2.09044
H	-1.24186	-5.38312	1.74784
C	-1.12242	-4.03886	3.42342
H	-3.39157	-3.22618	-0.45093
C	-3.66499	-5.29464	-0.00055
C	-1.55751	-4.22569	-0.89605
H	-1.15381	-2.50913	4.93020
H	-2.94551	0.07983	3.03400
C	-1.85994	-0.05103	4.85429
C	-4.24383	-0.82156	4.47724
C	-7.47868	1.67295	-2.12724
C	-7.48377	4.18014	-2.45463
H	-1.87156	1.68883	-2.05470
C	-0.15978	2.88904	-2.50227
C	-2.49195	3.20306	-3.42985
H	-0.87484	5.21139	-1.36113
C	-1.94313	5.43275	0.49249
H	-3.05187	5.35302	2.33473

H	-4.32286	1.84358	1.95619
C	-3.69023	3.01946	3.62279
C	-5.67720	3.48668	2.12289
C	-8.99507	-0.88096	-1.79446
C	-6.96035	-1.66305	-3.04577
H	-8.67487	-2.89547	-0.03333
H	-7.73213	-4.14508	3.17332
H	-8.84400	-4.38225	1.80150
H	-7.35242	-5.34373	1.92517
H	-0.46225	-4.69269	3.99848
H	-3.11718	-6.15237	0.42266
H	-3.96343	-5.56256	-1.02712
H	-4.57891	-5.14600	0.59137
H	-0.87903	-3.36194	-0.86046
H	-1.85047	-4.38213	-1.94646
H	-0.97325	-5.10394	-0.58439
H	-1.77629	-0.66059	5.76836
H	-2.19097	0.95079	5.16421
H	-0.85681	0.04211	4.41076
H	-4.97555	-1.16858	3.73363
H	-4.60773	0.13139	4.89429
H	-4.21337	-1.56074	5.29465
H	-8.49679	1.65653	-2.51634
H	-7.40716	4.96419	-1.68440
H	-7.00679	4.57797	-3.36652
H	-8.54922	4.02221	-2.67672
H	0.10612	3.90765	-2.82445
H	0.13441	2.20265	-3.31136
H	0.44703	2.64899	-1.62024
H	-3.56668	3.03579	-3.27256
H	-2.18635	2.65158	-4.33420
H	-2.34123	4.27802	-3.62403
H	-1.54428	6.43115	0.68921
H	-2.65671	2.64843	3.66605
H	-4.30119	2.42617	4.32055
H	-3.69334	4.05846	3.98854
H	-5.67434	4.56640	2.34685
H	-6.33583	2.99059	2.85436
H	-6.11100	3.34921	1.12253
H	-9.39202	-0.37014	-0.90508
H	-9.41756	-1.89523	-1.83139
H	-9.35287	-0.35142	-2.68894
H	-7.28908	-1.12634	-3.94914
H	-7.35468	-2.69048	-3.07922
H	-5.86198	-1.71725	-3.06079

C_SVP

198

SCF Done: E(RB3LYP) = -4488.74073197 A.U.

C	-1.56337	-0.50561	-1.22783
O	-1.16044	-0.84346	-2.28271
C	-0.68071	0.08780	0.03103
C	0.68057	0.08774	-0.03144
C	1.56324	-0.50520	1.22780
O	-1.50475	0.35326	0.96525
O	1.50469	0.35274	-0.96570
O	1.16019	-0.84258	2.28277
Al	3.17750	-0.06579	-0.12008
N	3.70283	-1.57568	-1.11001
N	3.71810	1.70161	0.23349
C	4.99627	-2.04083	-0.88430
C	2.82373	-2.44948	-1.82314
C	4.93340	1.86953	0.88889
C	2.99211	2.89632	-0.06376
C	5.68356	-1.42764	0.16973
C	5.70762	-3.02739	-1.58531
C	2.45538	-3.68305	-1.22830
C	2.34383	-2.09262	-3.10467
C	5.62747	0.69102	1.18966
C	5.59293	3.06491	1.22227
C	2.21255	3.51172	0.94284
C	3.11044	3.47769	-1.34857
O	4.91750	-0.45340	0.81380
C	6.99965	-1.63779	0.53458
C	7.04178	-3.31504	-1.24570
H	5.22206	-3.55748	-2.40599
C	1.66667	-4.57301	-1.96623
C	2.84813	-4.04973	0.19821
C	1.52977	-3.00429	-3.78963
C	2.74567	-0.77632	-3.75342
C	6.93588	0.59286	1.62325
H	5.10585	4.02056	1.02549
C	6.89806	3.03285	1.74606
C	2.01386	2.87845	2.31296
C	1.61647	4.74814	0.66406
C	2.49330	4.71305	-1.58164
C	3.91529	2.79374	-2.44492
C	7.55604	-0.81345	1.71071
C	7.68315	-2.61753	-0.20662
C	7.77695	-4.39101	-2.00744
H	1.38793	-5.53231	-1.52519
C	1.20827	-4.24311	-3.23895
H	3.42174	-3.21376	0.61840
C	3.74459	-5.29537	0.25708
C	1.60320	-4.22954	1.07838
H	1.14688	-2.74577	-4.77699
H	2.85617	-0.03765	-2.95019
C	1.68211	-0.22853	-4.70976
C	4.11500	-0.90705	-4.43916
C	7.57130	1.81142	1.92013
C	7.57503	4.33273	2.10689
H	2.47454	1.88130	2.28482
C	0.52462	2.69840	2.64443
C	2.72804	3.67476	3.41673
H	1.01788	5.23767	1.43342
C	1.76844	5.35541	-0.57883
H	2.58407	5.18496	-2.56104
H	3.86163	1.71089	-2.25512
C	3.34430	3.03528	-3.84583
C	5.40121	3.18453	-2.38845
C	9.08616	-0.75569	1.68220
C	7.09130	-1.47714	3.03159
H	8.72527	-2.84529	0.01852
H	7.66764	-4.25767	-3.09547
H	8.85066	-4.39490	-1.77007
H	7.37632	-5.38997	-1.76416
H	0.58290	-4.94461	-3.79614
H	3.21828	-6.18333	-0.12954
H	4.03879	-5.50968	1.29726
H	4.66111	-5.15684	-0.33375
H	0.92863	-3.36447	1.02320
H	1.88542	-4.36360	2.13461
H	1.02206	-5.11334	0.77536
H	1.58077	-0.84826	-5.61514
H	1.95625	0.78316	-5.04095
H	0.70130	-0.17723	-4.21436
H	4.88881	-1.22900	-3.72676
H	4.42993	0.05632	-4.87232
H	4.07355	-1.64900	-5.25349
H	8.60015	1.81983	2.28018
H	7.45279	5.08275	1.30942
H	7.13799	4.76384	3.02382
H	8.65150	4.19404	2.28425
H	0.01519	3.66770	2.75599
H	0.40467	2.14485	3.58913
H	-0.01356	2.14385	1.86384
H	3.80539	3.76180	3.21473
H	2.60244	3.17934	4.39309
H	2.31249	4.69241	3.50144
H	1.30433	6.32500	-0.77509
H	2.26925	2.80780	-3.88589
H	3.85945	2.39625	-4.57906
H	3.48560	4.07800	-4.17203
H	5.52040	4.26801	-2.55467
H	5.97085	2.65519	-3.16964
H	5.84921	2.94281	-1.41541
H	9.45527	-0.29006	0.75670
H	9.51306	-1.76595	1.75853
H	9.46801	-0.18042	2.53764
H	7.44590	-0.89444	3.89579
H	7.49021	-2.50067	3.10538
H	5.99421	-1.53208	3.08289
Al	-3.17758	-0.06583	0.12001
N	-3.70229	-1.57578	1.11020
N	-3.71885	1.70130	-0.23367
C	-4.99552	-2.04152	0.88458
C	-2.82282	-2.44900	1.82359
C	-4.93425	1.86871	-0.88901
C	-2.99310	2.89626	0.06317
C	-5.68312	-1.42870	-0.16946
C	-5.70645	-3.02829	1.58575
C	-2.45364	-3.68242	1.22898
C	-2.34331	-2.09165	3.10514
C	-5.62794	0.68992	-1.18953
C	-5.59418	3.06382	-1.22260
C	-2.21363	3.51141	-0.94365
C	-3.11152	3.47808	1.34777
O	-4.91752	-0.45419	-0.81362
C	-6.99918	-1.63935	-0.53415
C	-7.04053	-3.31647	1.24628
H	-5.22062	-3.55810	2.40645
C	-1.66456	-4.57183	1.96720
C	-2.84584	-4.04949	-0.19758
C	-1.52890	-3.00277	3.79039
C	-2.74582	-0.77533	3.75345
C	-6.93634	0.59125	-1.62302
H	-5.10738	4.01966	-1.02605
C	-6.89933	3.03123	-1.74629
C	-2.01483	2.87767	-2.31353
C	-1.61772	4.74803	-0.66532
C	-2.49456	4.71361	1.58039

C	-3.91625	2.79437	2.44436
C	-7.55601	-0.81530	-1.71027
C	-7.68225	-2.61928	0.20720
C	-7.77524	-4.39263	2.00820
H	-1.38519	-5.53103	1.52634
C	-1.20663	-4.24150	3.23996
H	-3.41951	-3.21371	-0.61811
C	-3.74197	-5.29537	-0.25648
C	-1.60060	-4.22922	-1.07733
H	-1.14631	-2.74388	4.77778
H	-2.85606	-0.03685	2.95000
C	-1.68289	-0.22708	4.71021
C	-4.11545	-0.90619	4.43856
C	-7.57218	1.80954	-1.92007
C	-7.57678	4.33082	-2.10725
H	-2.47535	1.88045	-2.28502
C	-0.52558	2.69773	-2.64496
C	-2.72919	3.67345	-3.41756
H	-1.01922	5.23736	-1.43487
C	-1.76978	5.35572	0.57736
H	-2.58541	5.18586	2.55962
H	-3.86244	1.71147	2.25488
C	-3.34520	3.03643	3.84517
C	-5.40221	3.18495	2.38788
C	-9.08615	-0.75806	-1.68157
C	-7.09122	-1.47892	-3.03117
H	-8.72430	-2.84746	-0.01783
H	-7.66603	-4.25902	3.09621
H	-8.84893	-4.39705	1.77079
H	-7.37415	-5.39145	1.76513
H	-0.58099	-4.94258	3.79738
H	-3.21555	-6.18307	0.13059
H	-4.03568	-5.51004	-1.29672
H	-4.65875	-5.15694	0.33394
H	-0.92614	-3.36407	-1.02203
H	-1.88247	-4.36340	-2.13363
H	-1.01945	-5.11293	-0.77408
H	-1.58179	-0.84664	5.61574
H	-1.95748	0.78458	5.04110
H	-0.70184	-0.17556	4.21528
H	-4.88885	-1.22855	3.72592
H	-4.43082	0.05724	4.87127
H	-4.07419	-1.64787	5.25315
H	-8.60107	1.81756	-2.28004
H	-7.45528	5.08080	-1.30962
H	-7.13952	4.76228	-3.02391
H	-8.65310	4.19163	-2.28512
H	-0.01629	3.66705	-2.75692
H	-0.40556	2.14380	-3.58943
H	0.01271	2.14359	-1.86414
H	-3.80655	3.76036	-3.21552
H	-2.60356	3.17770	-4.39375
H	-2.31384	4.69115	-3.50266
H	-1.30581	6.32544	0.77328
H	-2.27014	2.80902	3.88527
H	-3.86030	2.39765	4.57865
H	-3.48654	4.07926	4.17101
H	-5.52153	4.26849	2.55357
H	-5.97169	2.65593	3.16940
H	-5.85031	2.94269	1.41502
H	-9.45531	-0.29250	-0.75605
H	-9.51271	-1.76848	-1.75777
H	-9.46832	-0.18299	-2.53699
H	-7.44613	-0.89640	-3.89536
H	-7.48978	-2.50260	-3.10483
H	-5.99411	-1.53349	-3.08261

D_SVP

200

SCF Done: E(RB3LYP) = -5690.32630903 A.U.

Al	-3.14853	0.00697	0.23888
O	-5.06965	-0.03269	-0.76291
O	-2.00092	-0.01453	-1.22701
O	-1.75165	0.06299	1.51325
N	-3.80059	-1.78495	0.58660
N	-3.85525	1.78743	0.53751
C	-0.61424	-0.00269	0.83513
C	-5.82682	1.12201	-0.58055
C	-5.80347	-1.19165	-0.52165
C	-5.14933	2.10400	0.14538
C	-0.74972	-0.00023	-0.73126
C	-7.15417	1.17071	-0.97206
C	-7.79338	-2.47750	-0.57221
H	-8.83770	-2.62068	-0.85047
C	-5.09879	-2.13272	0.23165
C	-2.98825	-2.90941	0.89106
C	-7.73082	-0.08173	-1.64930
C	-2.54248	-3.15235	2.21483
C	-5.79222	-3.31825	0.53741
H	-5.29398	-4.10226	1.10947
C	-7.12644	-3.49285	0.13221
C	-7.84374	2.36196	-0.69154
H	-8.88934	2.47146	-0.98001
C	-7.13011	-1.28551	-0.90736
C	-5.86866	3.28715	0.39757
H	-5.39370	4.10174	0.94598
C	-7.20185	3.41908	-0.02664
C	-2.69991	-3.84763	-0.14495
C	-3.08003	2.91940	0.90119
C	-1.98852	-5.01305	0.17521
H	-1.76492	-5.73739	-0.61129
C	-7.83239	-4.78785	0.45451
C	-2.89192	-2.20621	3.35109
H	-3.23409	-1.27180	2.89463
C	-1.55101	-5.26076	1.47902
H	-0.99916	-6.17511	1.71018
C	-2.70518	3.13887	2.25109
C	-3.13071	-3.61632	-1.59322
H	-3.80379	-2.75390	-1.60883
C	-9.26106	-0.09579	-1.61476
H	-9.64227	-0.07187	-0.58341
H	-9.65089	-0.99744	-2.10890
H	-9.66824	0.77026	-2.15630
C	-3.14388	2.18561	3.34985
H	-3.43869	1.25024	2.86256
C	-1.82185	-4.32874	2.48168
H	-1.48226	-4.52491	3.50076
C	-7.25186	-0.11699	-3.12252
H	-7.63414	0.75965	-3.66841
H	-7.61524	-1.03002	-3.61949
H	-6.15404	-0.10685	-3.18216
C	-7.93504	4.71253	0.23519
C	-2.74749	3.87696	-0.10114
C	-1.94495	-3.24614	-2.50280
H	-1.54527	-2.25229	-2.24769
H	-2.27691	-3.18121	-3.55059
H	-1.13840	-3.99861	-2.46773
C	-1.98201	4.29950	2.57232
H	-1.68875	4.47555	3.60924
C	-1.67609	-1.86895	4.22077
H	-1.27774	-2.75395	4.74338
H	-1.95204	-1.13340	4.99089
H	-0.87415	-1.42958	3.60995
C	-3.91287	-4.81280	-2.15187
H	-3.27835	-5.70786	-2.25852
H	-4.31377	-4.57282	-3.14924
H	-4.75979	-5.06735	-1.49780
C	-3.12372	3.67452	-1.56854
H	-3.83188	2.84194	-1.62008
C	-4.05504	-2.77428	4.18029
H	-4.93771	-2.95719	3.54836
H	-4.34529	-2.06744	4.97461
H	-3.77785	-3.72763	4.66067
C	-4.38386	2.74407	4.06789
H	-4.15736	3.69848	4.57211
H	-4.74141	2.03401	4.83124
H	-5.20525	2.92227	3.35727
C	-2.03907	5.02868	0.27262
H	-1.77574	5.76642	-0.48837
C	-1.65187	5.24347	1.59790
H	-1.09718	6.14492	1.86953
C	-2.01600	1.85207	4.33041
H	-1.15883	1.41581	3.79775
H	-2.36430	1.11468	5.06963
H	-1.67432	2.73647	4.89259
C	-1.91519	3.26163	-2.42684
H	-1.08799	3.98770	-2.35859
H	-2.20427	3.20260	-3.48767
H	-1.55584	2.25786	-2.15217
C	-3.83088	4.90537	-2.15096
H	-4.69402	5.19229	-1.53201
H	-4.19814	4.68721	-3.16610
H	-3.15679	5.77433	-2.22530
O	2.00039	0.01281	1.22528
O	1.75232	-0.06161	-1.51527
C	0.61410	-0.01584	-0.83691
C	0.74955	-0.02064	0.72955
Al	3.14846	-0.00629	-0.24016
O	5.06926	0.03560	0.76282
N	3.79846	1.78652	-0.58638
N	3.85783	-1.78580	-0.53922
C	5.82831	-1.11770	0.57961
C	5.80137	1.19593	0.52306
C	5.15253	-2.10028	-0.14711
C	7.15559	-1.16465	0.97163
C	7.78919	2.48492	0.57568
H	8.83313	2.62957	0.85463
C	5.09568	2.13638	-0.23005
C	2.98457	2.90954	-0.89197
C	7.72995	0.08798	1.65045
C	2.54145	3.15223	-2.21666

C	5.78734	3.32327	-0.53449
H	5.28817	4.10685	-1.10636
C	7.12102	3.49974	-0.12832
C	7.84712	-2.35459	0.69040
H	8.89278	-2.46269	0.97919
C	7.12766	1.29157	0.90950
C	5.87385	-3.28205	-0.40001
H	5.40034	-4.09702	-0.94911
C	7.20715	-3.41218	0.02441
C	2.69152	3.84634	0.14396
C	3.08433	-2.91972	-0.90024
C	1.97718	5.00976	-0.17703
H	1.74953	5.73279	0.60948
C	7.82505	4.79608	-0.44941
C	2.89573	2.20749	-3.35259
H	3.24077	1.27426	-2.89580
C	1.54244	5.25735	-1.48174
H	0.98855	6.17030	-1.71356
C	2.70773	-3.14201	-2.24918
C	3.12154	3.61642	1.59271
H	3.79475	2.75409	1.60949
C	9.26018	0.10452	1.61660
H	9.64188	0.08232	0.58540
H	9.64835	1.00626	2.11189
H	9.66852	-0.76146	2.15739
C	3.14068	-2.18841	-3.34991
H	3.43515	-1.25219	-2.86409
C	1.81856	4.32697	-2.48447
H	1.48111	4.52303	-3.50429
C	7.25028	0.12088	3.12349
H	7.63365	-0.75578	3.66859
H	7.61205	1.03391	3.62163
H	6.15245	0.10902	3.18263
C	7.94253	-4.70417	-0.23854
C	2.75607	-3.87705	0.10384
C	1.93567	3.24767	2.50274
H	1.53752	2.25230	2.25117
H	2.26701	3.18682	3.55096
H	1.12790	3.99850	2.46431
C	1.98829	-4.30572	-2.56797
H	1.69394	-4.48393	-3.60422
C	1.68218	1.86606	-4.22384
H	1.28102	2.74986	-4.74631
H	1.96187	1.13207	-4.99415
H	0.88142	1.42310	-3.61412
C	3.90333	4.81391	2.14993
H	3.26842	5.70887	2.25504
H	4.30404	4.57545	3.14775
H	4.75033	5.06780	1.49574
C	3.13088	-3.66996	1.57088
H	3.83346	-2.83260	1.62123
C	4.05783	2.77974	-4.18034
H	4.93908	2.96575	-3.54733
H	4.35156	2.07402	-4.97436
H	3.77785	3.73213	-4.66100
C	4.37921	-2.74467	-4.07209
H	4.15297	-3.69974	-4.57519
H	4.73266	-2.03417	-4.83695
H	5.20344	-2.92091	-3.36426
C	2.05221	-5.03225	-0.26756
H	1.79299	-5.77020	0.49469
C	1.66376	-5.25007	-1.59204
H	1.11247	-6.15416	-1.86183
C	2.00842	-1.85781	-4.32646
H	1.15235	-1.42291	-3.79094
H	2.35232	-1.12014	-5.06745
H	1.66641	-2.74331	-4.88672
C	1.91921	-3.26344	2.42780
H	1.09788	-3.99652	2.36260
H	2.20804	-3.19780	3.48830
H	1.55121	-2.26395	2.14914
C	3.84576	-4.89524	2.15560
H	4.71141	-5.17706	1.53784
H	4.21058	-4.67330	3.17081
H	3.17743	-5.76860	2.23042
H	8.91226	4.71340	-0.30447
H	7.64171	5.10651	-1.49033
H	7.46471	5.61407	0.19849
H	9.02748	-4.58847	-0.09915
H	7.60272	-5.50022	0.44672
H	7.76632	-5.06613	-1.26393
H	-7.59641	5.50643	-0.45316
H	-9.02053	4.59768	0.09924
H	-7.75543	5.07693	1.25911
H	-7.64657	-5.09947	1.49463
H	-8.91984	-4.70265	0.31292
H	-7.47588	-5.60616	-0.19512
K	-0.03859	2.69383	0.08427
K	0.03918	-2.71645	-0.08864

Toluene_SVP

15

SCF Done: E(RB3LYP) = -271.394519887   A.U.

C	-1.90652	-0.00046	0.00892
C	-1.20116	-1.20696	0.00212
C	0.19567	-1.20363	-0.00954
C	0.91650	0.00062	-0.01227
C	0.19487	1.20411	-0.00952
C	-1.20220	1.20639	0.00212
H	-2.99913	-0.00093	0.01526
H	-1.74165	-2.15693	0.00219
H	0.73880	-2.15296	-0.01871
H	0.73726	2.15383	-0.01868
H	-1.74331	2.15600	0.00217
C	2.42465	0.00026	0.00949
H	2.80490	-0.02689	1.04590
H	2.83616	-0.87825	-0.51093
H	2.83610	0.90416	-0.46516

E_SVP

230

SCF Done: E(RB3LYP) = -6231.87250233   A.U.

K	-0.69591	3.25814	-1.33326
Al	3.15384	-0.24058	0.11736
O	4.96146	0.56097	-0.56890
O	1.46302	-0.91803	0.65993
O	1.40250	1.81061	-1.48216
N	3.87402	-1.73128	-0.87840
N	3.84879	0.51689	1.74099
C	5.67678	-0.29270	-1.40575
C	5.04720	-1.52871	-1.59366
C	5.72091	-2.42593	-2.44459
H	5.27705	-3.39806	-2.66337
C	6.98852	-2.10601	-2.96351
C	7.61652	-0.89729	-2.62096
H	8.62386	-0.69759	-2.98710
C	6.95805	0.03998	-1.80620
C	7.52873	1.36847	-1.27876
C	7.04046	1.51192	0.17525
C	7.77372	2.00602	1.26742
H	8.80001	2.34847	1.13216
C	7.19661	2.05597	2.54771
C	5.88865	1.58666	2.76359
H	5.46452	1.60746	3.76841
C	5.12498	1.07104	1.69896
C	5.74445	1.11009	0.44496
C	7.97437	2.62263	3.71145
C	6.93643	2.53023	-2.11460
H	5.83892	2.52212	-2.07398
H	7.24274	2.43680	-3.16822
H	7.28844	3.49903	-1.72698
C	9.05618	1.40450	-1.36964
H	9.44321	2.36289	-0.99414
H	9.38422	1.31046	-2.41501
H	9.51462	0.59213	-0.78699
C	7.67411	-3.08631	-3.88447
C	3.15363	-2.90583	-1.22602
C	2.39137	-2.91889	-2.42013
C	1.72317	-4.09602	-2.78118
H	1.12796	-4.11127	-3.69694
C	1.79778	-5.24005	-1.98891
H	1.26921	-6.14922	-2.28584
C	2.54805	-5.22255	-0.81104
H	2.60366	-6.12693	-0.20273
C	3.23565	-4.06483	-0.40907
C	4.08127	-4.05337	0.86247
H	3.96291	-3.05636	1.31532
C	3.66088	-5.10409	1.89826
H	2.58584	-5.08490	2.13724
H	4.20899	-4.94575	2.83883
H	3.89245	-6.12528	1.55717
C	5.57995	-4.21586	0.55746
H	5.76973	-5.18651	0.07036
H	6.16585	-4.18015	1.49009
H	5.94869	-3.42570	-0.10631
C	2.29648	-1.68794	-3.31107
H	2.89999	-0.89766	-2.84888
C	2.88157	-1.95326	-4.70670
H	3.92702	-2.28993	-4.64550
H	2.85520	-1.03247	-5.31138
H	2.30284	-2.72055	-5.24711
C	0.85766	-1.17057	-3.40560
H	0.20375	-1.88788	-3.91587
H	0.82270	-0.22745	-3.97195
H	0.42769	-0.97489	-2.41490
C	3.09988	0.88787	2.89414
C	2.79448	-0.06406	3.89161
C	2.24800	0.37565	5.10440
H	2.02730	-0.34696	5.89223
C	2.00229	1.72918	5.33295
H	1.59893	2.05852	6.29348
C	2.22671	2.65142	4.31151

H	1.97698	3.70358	4.47131
C	2.74445	2.24807	3.07527
C	2.87040	3.25401	1.93813
H	3.30846	2.73166	1.07918
C	3.78624	4.43647	2.27897
H	3.39201	5.02200	3.12584
H	3.87301	5.11939	1.41780
H	4.79652	4.09044	2.54047
C	1.47719	3.73567	1.50782
H	0.80668	2.88402	1.32153
H	1.54356	4.34399	0.59227
H	1.00736	4.35435	2.28522
C	3.11764	-1.52948	3.65577
H	3.04955	-1.68360	2.56994
C	4.56257	-1.85066	4.06446
H	5.27194	-1.20675	3.52544
H	4.81523	-2.90040	3.84264
H	4.70566	-1.68638	5.14496
C	2.11606	-2.48203	4.31833
H	2.19639	-2.47079	5.41664
H	2.30244	-3.52095	4.00331
H	1.07732	-2.21274	4.06645
C	0.69016	-0.01483	-0.01576
C	1.67261	0.85263	-0.74853
C	1.64720	6.01219	-1.64607
H	2.10223	6.33802	-0.70743
C	0.46578	6.62175	-2.09033
H	0.00779	7.41497	-1.49399
C	-0.12254	6.22128	-3.29494
H	-1.03842	6.70037	-3.64850
C	0.48165	5.20489	-4.04945
H	0.03156	4.88336	-4.99195
C	1.65418	4.59371	-3.59587
H	2.10609	3.78591	-4.17711
C	2.25954	4.98597	-2.38581
C	3.49592	4.28188	-1.89505
H	3.25372	3.22587	-1.69173
H	3.88714	4.73558	-0.97374
H	4.29367	4.30587	-2.65375
O	-1.46304	0.91805	-0.65992
O	-1.40248	-1.81059	1.48216
C	-0.69016	0.01486	0.01576
C	-1.67260	-0.85259	0.74855
K	0.69597	-3.25806	1.33363
Al	-3.15385	0.24058	-0.11739
O	-4.96145	-0.56097	0.56893
N	-3.87411	1.73136	0.87822
N	-3.84876	-0.51699	-1.74098
C	-5.67682	0.29276	1.40568
C	-5.04729	1.52882	1.59348
C	-5.72105	2.42610	2.44431
H	-5.27722	3.39827	2.66299
C	-6.98866	2.10619	2.96324
C	-7.61661	0.89741	2.62079
H	-8.62396	0.69772	2.98691
C	-6.95809	-0.03991	1.80614
C	-7.52872	-1.36846	1.27880
C	-7.04043	-1.51199	-0.17520
C	-7.77368	-2.00615	-1.26735
H	-8.79998	-2.34857	-1.13208
C	-7.19656	-2.05614	-2.54764
C	-5.88861	-1.58683	-2.76353
H	-5.46447	-1.60764	-3.76834
C	-5.12494	-1.07116	-1.69891
C	-5.74442	-1.11017	-0.44491
C	-6.93640	-2.53014	2.11472
H	-5.83888	-2.52201	2.07411
H	-7.24272	-2.43664	3.16833
H	-7.28838	-3.49898	1.72717
C	-9.05617	-1.40454	1.36966
H	-9.44317	-2.36296	0.99423
H	-9.38423	-1.31043	2.41502
H	-9.51464	-0.59222	0.78695
C	-3.15376	2.90595	1.22573
C	-2.39149	2.91914	2.41984
C	-1.72329	4.09631	2.78076
H	-1.12809	4.11166	3.69653
C	-1.79788	5.24025	1.98837
H	-1.26930	6.14945	2.28519
C	-2.54818	5.22263	0.81051
H	-2.60380	6.12695	0.20211
C	-3.23579	4.06488	0.40867
C	-4.08145	4.05328	-0.86284
H	-3.96314	3.05620	-1.31556
C	-3.66103	5.10382	-1.89880
H	-2.58599	5.08453	-2.13779
H	-4.20915	4.94537	-2.83934
H	-3.89254	6.12508	-1.55786
C	-5.58010	4.21588	-0.55781
H	-5.76984	5.18662	-0.07088

H	-6.16604	4.18003	-1.49042
H	-5.94886	3.42584	0.10610
C	-2.29665	1.68830	3.31094
H	-2.90018	0.89799	2.84884
C	-2.88174	1.95382	4.70653
H	-3.92717	2.29054	4.64528
H	-2.85544	1.03310	5.31132
H	-2.30297	2.72114	5.24685
C	-0.85785	1.17089	3.40554
H	-0.20390	1.88827	3.91564
H	-0.82291	0.22790	3.97211
H	-0.42792	0.97496	2.41487
C	-3.09986	-0.88807	-2.89409
C	-2.79440	0.06378	-3.89164
C	-2.24799	-0.37608	-5.10441
H	-2.02726	0.34646	-5.89231
C	-2.00237	-1.72964	-5.33286
H	-1.59906	-2.05908	-6.29338
C	-2.22677	-2.65177	-4.31132
H	-1.97707	-3.70395	-4.47102
C	-2.74446	-2.24829	-3.07511
C	-2.87036	-3.25408	-1.93783
H	-3.30840	-2.73162	-1.07894
C	-3.78618	-4.43661	-2.27847
H	-3.39196	-5.02224	-3.12527
H	-3.87290	-5.11941	-1.41720
H	-4.79647	-4.09064	-2.53999
C	-1.47712	-3.73563	-1.50750
H	-0.80662	-2.88392	-1.32140
H	-1.54343	-4.34378	-0.59184
H	-1.00731	-4.35443	-2.28481
C	-3.11737	1.52925	-3.65588
H	-3.04942	1.68339	-2.57005
C	-4.56219	1.85063	-4.06481
H	-5.27174	1.20686	-3.52588
H	-4.81472	2.90042	-3.84308
H	-4.70513	1.68634	-5.14533
C	-2.11553	2.48162	-4.31830
H	-2.19565	2.47034	-5.41662
H	-2.30180	3.52057	-4.00335
H	-1.07688	2.21218	-4.06619
C	-1.64718	-6.01210	1.64648
H	-2.10227	-6.33794	0.70788
C	-0.46574	-6.62166	2.09069
H	-0.00778	-7.41487	1.49432
C	0.12264	-6.22118	3.29526
H	1.03854	-6.70026	3.64879
C	-0.48152	-5.20478	4.04980
H	-0.03138	-4.88326	4.99228
C	-1.65407	-4.59361	3.59628
H	-2.10597	-3.78582	4.17754
C	-2.25949	-4.98587	2.38624
C	-3.49587	-4.28177	1.89552
H	-3.25362	-3.22579	1.69204
H	-3.88720	-4.73554	0.97430
H	-4.29355	-4.30561	2.65430
C	-7.97427	-2.62303	-3.71130
H	-7.58166	-3.61140	-4.00676
H	-9.03898	-2.74836	-3.46505
H	-7.90278	-1.97249	-4.59759
C	-7.67428	3.08652	3.88413
H	-8.73945	2.84419	4.01348
H	-7.20816	3.08247	4.88466
H	-7.60201	4.11688	3.50070
H	7.58067	3.61016	4.00826
H	7.90431	1.97103	4.59709
H	9.03875	2.74957	3.46463
H	8.73913	-2.84363	-4.01433
H	7.60239	-4.11661	-3.50075
H	7.20755	-3.08268	-4.88479

F_SVP

246

SCF Done: E(RB3LYP) = -7413.11017902 A.U.

K	0.15561	-1.87282	2.22147
K	0.54707	1.93003	2.80925
Al	-3.11649	0.03939	-0.22353
O	-1.47501	0.10839	-1.17102
O	-4.96386	-0.05928	0.78144
O	-1.28585	0.25994	2.41117
N	-3.94295	1.76097	-0.70628
N	-3.77335	-1.75236	-0.74601
C	-0.73831	0.17536	0.04960
C	-1.63522	0.17557	1.10555
C	-5.70596	-1.20957	0.52564
C	-5.05624	-2.10504	-0.32710
C	-5.73129	1.10253	0.70211
C	-7.00548	-1.32121	0.99299
C	-5.15264	2.08707	-0.10771
C	-7.54301	-0.16816	1.85625
C	-7.01729	1.12569	1.21335

C	-3.09762	-2.86092	-1.33571
C	-7.74338	2.31466	1.02955
H	-8.75503	2.40768	1.42445
C	-3.36081	2.82532	-1.44286
C	-7.18450	3.38547	0.31624
C	-2.60950	-3.90954	-0.49843
C	-2.75321	3.91767	-0.76580
C	-2.96551	-1.32168	3.60238
H	-3.62499	-1.39072	2.71911
H	-2.20641	-2.11704	3.44960
C	-5.80005	-3.24709	-0.68643
H	-5.35781	-3.99559	-1.34383
C	-9.07214	-0.17998	1.93158
H	-9.52969	-0.08452	0.93592
H	-9.43316	0.64606	2.56108
H	-9.42814	-1.11340	2.39094
C	-5.91080	3.26428	-0.26490
H	-5.52599	4.07657	-0.88190
C	-7.10661	-3.43835	-0.21013
C	-3.42154	1.34408	3.50864
H	-3.64680	1.49429	2.43970
H	-4.36965	0.97988	3.93828
C	-3.04350	-3.01277	-2.75022
C	-7.70799	-2.47764	0.61796
H	-8.73239	-2.63186	0.95714
C	-2.22458	4.97834	-1.51723
H	-1.75420	5.81671	-0.99830
C	-3.46835	2.85131	-2.86035
C	-1.32800	0.38549	4.95744
H	-0.93389	1.42166	5.02088
H	-1.98920	0.31813	5.83920
C	-2.58230	-3.78616	1.02183
H	-2.55700	-2.70980	1.24638
C	-7.94745	4.67781	0.15486
C	-2.29942	4.99358	-2.90905
H	-1.88591	5.83072	-3.47666
C	-2.92537	3.93437	-3.57029
H	-3.00919	3.96398	-4.65762
C	-2.54312	-4.21060	-3.29037
H	-2.51005	-4.34443	-4.37082
C	-2.68018	3.98561	0.75279
H	-3.12857	3.06967	1.15350
C	-2.13587	-5.08979	-1.08994
H	-1.77748	-5.90210	-0.45636
C	-7.85175	-4.69627	-0.58586
C	-6.96010	-0.31104	3.28198
H	-7.30475	-1.25017	3.74157
H	-7.27986	0.53265	3.91297
H	-5.86428	-0.32314	3.26059
C	-3.12857	2.70053	4.15605
H	-2.17064	3.13269	3.81751
H	-3.05570	2.61950	5.25299
H	-3.90481	3.45466	3.93941
C	-3.76348	-1.70041	4.85341
H	-3.10866	-1.80059	5.73564
H	-4.31203	-2.65299	4.74569
H	-4.50824	-0.92790	5.10410
C	-2.10821	-5.25072	-2.47199
H	-1.73969	-6.18007	-2.91253
C	-3.54436	-1.91657	-3.68743
H	-3.14167	-0.96431	-3.30258
C	-1.33746	-4.45595	1.62456
H	-1.40789	-5.55317	1.58994
H	-1.24050	-4.19907	2.69194
H	-0.40560	-4.19769	1.10070
C	-3.45919	5.18560	1.31325
H	-3.03695	6.14130	0.96109
H	-3.41600	5.18328	2.41265
H	-4.51648	5.14604	1.01887
C	-4.22272	1.75325	-3.59411
H	-4.09983	0.83342	-3.00599
C	-0.16034	-0.58347	5.16119
H	0.34226	-0.47776	6.13774
H	0.63951	-0.44498	4.40956
H	-0.49036	-1.63588	5.09311
C	-1.22503	4.02397	1.22441
H	-0.68559	3.14685	0.84117
H	-1.18686	4.03916	2.32356
H	-0.69815	4.92128	0.87296
B	-2.27941	0.17138	3.62569
C	-3.05379	-2.09568	-5.12993
H	-1.96932	-2.25884	-5.19424
H	-3.28778	-1.20363	-5.72453
H	-3.55117	-2.94916	-5.61713
C	-3.69972	1.49491	-5.01027
H	-3.84539	2.36371	-5.67036
H	-4.24644	0.65858	-5.46629
H	-2.62781	1.23577	-5.02781
C	-3.81835	-4.36918	1.73011
H	-4.74038	-3.84417	1.46650
H	-3.68737	-4.29459	2.82053
H	-3.94328	-5.43346	1.46969
C	-5.07833	-1.79815	-3.68730
H	-5.53758	-2.76507	-3.94682
H	-5.40196	-1.06144	-4.43810
H	-5.47107	-1.48777	-2.71385
C	-5.72976	2.06003	-3.63048
H	-6.14000	2.17887	-2.61955
H	-6.27750	1.24411	-4.12806
H	-5.92125	2.99165	-4.18828
C	0.73825	0.17537	-0.04952
O	1.47494	0.10804	1.17108
C	1.63517	0.17597	-1.10546
Al	3.11645	0.03936	0.22359
O	1.28581	0.26082	-2.41106
N	3.94293	1.76078	0.70686
N	3.77339	-1.75252	0.74560
K	-0.15551	-1.87214	-2.22199
K	-0.54753	1.93059	-2.80913
B	2.27943	0.17262	-3.62555
C	5.15259	2.08708	0.10833
C	3.36070	2.82495	1.44365
C	5.05631	-2.10503	0.32665
C	3.09771	-2.86131	1.33494
C	2.96553	-1.32045	-3.60267
C	3.42159	1.34527	-3.50805
C	1.32812	0.38713	-4.95730
C	5.73126	1.10281	-0.70178
C	5.91069	3.26431	0.26583
C	2.75335	3.91755	0.76677
C	3.46790	2.85050	2.86116
C	5.70603	-1.20933	-0.52584
C	5.80015	-3.24714	0.68574
C	2.60973	-3.90971	0.49730
C	3.04351	-3.01361	2.74940
H	3.62489	-1.38978	-2.71934
H	2.20645	-2.11589	-3.45029
C	3.76370	-1.69868	-4.85372
H	3.64619	1.49565	-2.43899
H	4.36995	0.98090	-3.93699
C	3.12920	2.70163	-4.15591
H	0.93404	1.42333	-5.02048
H	1.98937	0.32001	-5.83904
C	0.16047	-0.58178	-5.16141
O	4.96389	-0.05902	-0.78143
C	7.01730	1.12615	-1.21296
C	7.18436	3.38573	-0.31528
H	5.52583	4.07642	0.88304
C	2.22451	4.97797	1.51836
C	2.68073	3.98587	-0.75182
C	2.92474	3.93334	3.57129
C	4.22215	1.75223	3.59473
C	7.00559	-1.32080	-0.99314
H	5.35792	-3.99583	1.34293
C	7.10674	-3.43823	0.20947
C	2.58264	-3.78584	-1.02291
C	2.13613	-5.09017	1.08840
C	2.54319	-4.21164	3.28914
C	3.54426	-1.91768	3.68700
H	3.10901	-1.79862	-5.73608
H	4.31232	-2.65125	-4.74625
H	4.50842	-0.92602	-5.10405
H	2.17106	3.13394	-3.81817
H	3.05711	2.62039	-5.25289
H	3.90536	3.45573	-3.93885
H	-0.34210	-0.47575	-6.13794
H	-0.63941	-0.44360	-4.40976
H	0.49052	-1.63421	-5.09368
C	7.54314	-0.16754	-1.85609
C	7.74333	2.31509	-1.02882
C	7.94718	4.67816	-0.15405
H	1.75426	5.81650	0.99956
C	2.29895	4.99276	2.91022
H	3.13014	3.07051	-1.15268
C	3.45873	5.18674	-1.31176
C	1.22568	4.02278	-1.22383
H	3.00826	3.96261	4.65866
H	4.09931	0.83255	3.00637
C	3.69901	1.49352	5.01076
C	5.72919	2.05901	3.63131
C	7.70813	-2.47729	-0.61835
C	7.85191	-4.69623	0.58487
H	2.55735	-2.70942	-1.24711
C	1.33785	-4.45544	-1.62595
C	3.81876	-4.36861	-1.73128
H	1.77785	-5.90231	0.45454
C	2.10839	-5.25153	2.47041
H	2.51006	-4.34582	4.36954
H	3.14144	-0.96533	3.30250
C	3.05377	-2.09736	5.12945

C	5.07822	-1.79904	3.68688
C	9.07227	-0.17925	-1.93130
C	6.96037	-0.31015	-3.28191
H	8.75501	2.40823	-1.42362
H	1.88528	5.82971	3.47798
H	3.03527	6.14199	-0.95985
H	3.41618	5.18440	-2.41120
H	4.51590	5.14833	-1.01677
H	0.68726	3.14482	-0.84105
H	1.18784	4.03824	-2.32298
H	0.69768	4.91936	-0.87219
H	3.84462	2.36215	5.67111
H	4.24567	0.65707	5.46662
H	2.62710	1.23438	5.02812
H	6.13950	2.17812	2.62043
H	6.27690	1.24297	4.12871
H	5.92063	2.99049	4.18936
H	8.73256	-2.63137	-0.95750
H	1.40826	-5.55267	-1.59170
H	1.24095	-4.19818	-2.69325
H	0.40595	-4.19736	-1.10205
H	4.74077	-3.84371	-1.46736
H	3.68790	-4.29361	-2.82169
H	3.94366	-5.43299	-1.47125
H	1.73990	-6.18105	2.91064
H	1.96932	-2.26068	5.19374
H	3.28767	-1.20550	5.72435
H	3.55127	-2.95096	5.61632
H	5.53761	-2.76602	3.94593
H	5.40176	-1.06265	4.43802
H	5.47089	-1.48815	2.71356
H	9.52973	-0.08399	-0.93558
H	9.43329	0.64694	-2.56059
H	9.42836	-1.11255	-2.39084
H	7.30516	-1.24914	-3.74168
H	7.28007	0.53372	-3.91268
H	5.86454	-0.32240	-3.26062
H	7.54273	5.46195	-0.81813
H	7.87638	5.06203	0.87625
H	9.01208	4.55387	-0.40020
H	8.93138	-4.59914	0.39664
H	7.71131	-4.94473	1.64884
H	7.48972	-5.56059	0.00151
H	-7.88048	5.05913	-0.87666
H	-9.01144	4.55453	0.40537
H	-7.54010	5.46309	0.81538
H	-7.71196	-4.94393	-1.65012
H	-7.48883	-5.56096	-0.00345
H	-8.93109	-4.59961	-0.39666

A_TZVP

198

SCF Done: E(RB3LYP) = -4495.03167268 A.U.

Al	3.21969	0.00003	0.21619
O	4.93002	0.00009	-0.87327
O	1.91223	-0.00010	-1.22261
O	1.69491	0.00005	1.50125
N	3.76287	1.75832	0.51798
N	3.76319	-1.75807	0.51838
C	0.67195	0.00005	0.77712
C	5.68662	-1.16720	-0.70508
C	5.68633	1.16763	-0.70549
C	5.02257	-2.12348	0.05018
C	0.78321	0.00002	-0.66276
C	6.98272	-1.23278	-1.15656
C	7.65138	2.43471	-0.90374
H	8.67323	2.56694	-1.22918
C	5.02209	2.12394	0.04957
C	2.95861	2.86324	0.93891
C	7.55321	0.00025	-1.86804
C	2.82075	3.16273	2.30319
C	5.71417	3.31914	0.26302
H	5.24457	4.11935	0.81803
C	7.01646	3.47472	-0.22000
C	7.65196	-2.43389	-0.90297
H	8.67385	-2.56596	-1.22835
C	6.98241	1.23338	-1.15698
C	5.71492	-3.31847	0.26396
H	5.24546	-4.11866	0.81911
C	7.01726	-3.47387	-0.21896
C	2.40896	3.70661	-0.04723
C	2.95899	-2.86322	0.93888
C	1.77447	4.87795	0.35711
H	1.35354	5.53842	-0.38929
C	7.73201	4.78200	-0.00170
C	3.39502	2.25614	3.37431
H	3.68725	1.32567	2.89003
C	1.68476	5.21323	1.69865
H	1.20587	6.13768	1.99671
C	2.82075	-3.16294	2.30309
C	2.52435	3.38524	-1.52729
H	3.02364	2.42437	-1.62014
C	9.08127	0.00044	-1.85845
H	9.47480	0.00067	-0.84183
H	9.46547	0.87731	-2.37898
H	9.46569	-0.87651	-2.37866
C	3.39427	-2.25625	3.37451
H	3.68606	-1.32551	2.89049
C	2.18628	4.35105	2.66070
H	2.09024	4.60945	3.70721
C	7.05611	-0.00008	-3.33270
H	7.42086	-0.88761	-3.85226
H	7.42065	0.88736	-3.85258
H	5.96741	-0.00022	-3.37882
C	7.73311	-4.78092	-0.00030
C	2.40987	-3.70657	-0.04755
C	1.14923	3.25507	-2.18660
H	0.48702	2.59741	-1.62301
H	1.24660	2.85541	-3.19777
H	0.64878	4.21812	-2.25510
C	2.18656	-4.35151	2.66020
H	2.09028	-4.61011	3.70664
C	2.36046	1.91180	4.44883
H	2.04002	2.79778	5.00017
H	2.78391	1.21107	5.17015
H	1.47872	1.44973	4.00483
C	3.38014	4.41886	-2.27089
H	2.92309	5.40989	-2.22742
H	3.47834	4.14228	-3.32292
H	4.37924	4.48494	-1.84151
C	2.52568	-3.38499	-1.52754
H	3.02465	-2.42392	-1.62010
C	4.66174	2.86317	3.99089
H	5.41693	3.05015	3.22672
H	5.08929	2.18622	4.73392
H	4.43962	3.81114	4.48635
C	4.66120	-2.86267	3.99127
H	4.43950	-3.81090	4.48643
H	5.08814	-2.18565	4.73459
H	5.41670	-3.04900	3.22726
C	1.77556	-4.87815	0.35641
H	1.35504	-5.53860	-0.39022
C	1.68557	-5.21369	1.69786
H	1.20687	-6.13834	1.99562
C	2.35928	-1.91271	4.44887
H	1.47733	-1.45113	4.00475
H	2.78213	-1.21179	5.17036
H	2.03927	-2.79895	5.00004
C	1.15078	-3.25524	-2.18736
H	0.65069	-4.21846	-2.25616
H	1.24842	-2.85545	-3.19845
H	0.48811	-2.59786	-1.62398
C	3.38214	-4.41821	-2.27092
H	4.38109	-4.48399	-1.84115
H	3.48065	-4.14149	-3.32288
H	2.92542	-5.40941	-2.22772
O	-1.91222	-0.00005	1.22257
O	-1.69490	0.00017	-1.50128
C	-0.67195	0.00010	-0.77715
C	-0.78320	0.00004	0.66272
Al	-3.21968	-0.00004	-0.21618
O	-4.93004	-0.00031	0.87322
N	-3.76284	-1.75827	-0.51833
N	-3.76318	1.75815	-0.51800
C	-5.68662	1.16703	0.70531
C	-5.68635	-1.16780	0.70516
C	-5.02255	2.12347	-0.04974
C	-6.98273	1.23252	1.15677
C	-7.65139	-2.43494	0.90317
H	-8.67324	-2.56724	1.22856
C	-5.02208	-2.12398	-0.05004
C	-2.95849	-2.86316	-0.93919
C	-7.55326	-0.00067	1.86794
C	-2.82037	-3.16254	-2.30348
C	-5.71413	-3.31916	-0.26368
H	-5.24450	-4.11928	-0.81878
C	-7.01644	-3.47484	0.21927
C	-7.65196	2.43369	0.90343
H	-8.67385	2.56570	1.22882
C	-6.98244	-1.23365	1.15663
C	-5.71489	3.31850	-0.26328
H	-5.24544	4.11880	-0.81828
C	-7.01724	3.47381	0.21965
C	-2.40902	-3.70660	0.04698
C	-2.95907	2.86333	-0.93854
C	-1.77449	-4.87793	-0.35733
H	-1.35370	-5.53845	0.38909
C	-7.73197	-4.78209	0.00073
C	-3.39437	-2.25580	-3.37462
H	-3.68607	-1.32513	-2.89041
C	-1.68458	-5.21313	-1.69887

H	-1.20569	-6.13759	-1.99691
C	-2.82107	3.16319	-2.30273
C	-2.52468	-3.38534	1.52704
H	-3.02397	-2.42446	1.61988
C	-9.08131	-0.00084	1.85831
H	-9.47480	-0.00084	0.84168
H	-9.46553	-0.87782	2.37862
H	-9.46574	0.87599	2.37871
C	-3.39483	2.25666	-3.37417
H	-3.68711	1.32609	-2.89012
C	-2.18591	-4.35087	-2.66095
H	-2.08969	-4.60920	-3.70746
C	-7.05620	-0.00069	3.33262
H	-7.42095	0.88673	3.85238
H	-7.42077	-0.88824	3.85228
H	-5.96750	-0.00058	3.37878
C	-7.73308	4.78091	0.00126
C	-2.40981	3.70663	0.04788
C	-1.14969	-3.25526	2.18663
H	-0.48732	-2.59762	1.62319
H	-1.24726	-2.85562	3.19778
H	-0.64931	-4.21834	2.25521
C	-2.18691	4.35178	-2.65985
H	-2.09080	4.61046	-3.70629
C	-2.35984	-1.91205	-4.44935
H	-2.04000	-2.79819	-5.00078
H	-2.78304	-1.21106	-5.17056
H	-1.47774	-1.45047	-4.00554
C	-3.38065	-4.41899	2.27039
H	-2.92363	-5.41004	2.22690
H	-3.47902	-4.14251	3.32243
H	-4.37967	-4.48499	1.84083
C	-2.52540	3.38492	1.52786
H	-3.02434	2.42383	1.62041
C	-4.66147	-2.86230	-3.99094
H	-5.41666	-3.04877	-3.22666
H	-5.08876	-2.18528	-4.73405
H	-4.43985	-3.81048	-4.48624
C	-4.66140	2.86358	-3.99117
H	-4.43923	3.81163	-4.48645
H	-5.08857	2.18665	-4.73443
H	-5.41690	3.05037	-3.22727
C	-1.77559	4.87824	-0.35608
H	-1.35498	5.53866	0.39054
C	-1.68578	5.21388	-1.69753
H	-1.20712	6.13855	-1.99529
C	-2.35979	1.91263	-4.44833
H	-1.47819	1.45057	-4.00405
H	-2.78286	1.21198	-5.16996
H	-2.03922	2.79875	-4.99936
C	-1.15039	3.25517	2.18746
H	-0.65036	4.21841	2.25631
H	-1.24785	2.85523	3.19851
H	-0.48777	2.59791	1.62388
C	-3.38178	4.41805	2.27146
H	-4.38080	4.48382	1.84186
H	-3.48011	4.14123	3.32341
H	-2.92511	5.40928	2.22827
H	-8.81096	-4.66631	0.10492
H	-7.52618	-5.18601	-0.99176
H	-7.40747	-5.53058	0.72907
H	-8.81207	4.66480	0.10508
H	-7.40903	5.52929	0.72992
H	-7.52710	5.18518	-0.99105
H	7.40896	-5.52949	-0.72871
H	8.81209	-4.66484	-0.10429
H	7.52725	-5.18491	0.99215
H	7.52618	5.18614	0.99070
H	8.81100	4.66618	-0.10582
H	7.40756	5.53035	-0.73021

E_TZVP

230

SCF Done: E(RB3LYP) = -6238.45718919 A.U.

K	-0.70695	3.26524	-1.30844
Al	3.14183	-0.23244	0.10864
O	4.93145	0.59110	-0.58821
O	1.47027	-0.90780	0.65129
O	1.38527	1.80803	-1.49277
N	3.86333	-1.70745	-0.88962
N	3.83815	0.51371	1.72543
C	5.65083	-0.26467	-1.42329
C	5.02900	-1.49497	-1.60985
C	5.70078	-2.38383	-2.45990
H	5.26706	-3.35036	-2.67567
C	6.95556	-2.05882	-2.98204
C	7.57803	-0.85675	-2.63953
H	8.57630	-0.65702	-3.00188
C	6.92233	0.07065	-1.82391
C	7.49769	1.38752	-1.28642
C	7.01103	1.51979	0.16431
C	7.74706	1.99526	1.25330
H	8.76569	2.33089	1.12025
C	7.17915	2.02924	2.52943
C	5.88050	1.56113	2.74446
H	5.46693	1.56983	3.74352
C	5.11329	1.06603	1.68141
C	5.72354	1.11872	0.43346
C	7.96337	2.58544	3.68952
C	6.91887	2.55970	-2.11154
H	5.83103	2.55961	-2.07839
H	7.23062	2.47445	-3.15407
H	7.27498	3.51181	-1.71410
C	9.02223	1.41730	-1.37672
H	9.40755	2.36293	-0.99546
H	9.34436	1.33172	-2.41445
H	9.47293	0.60484	-0.80645
C	7.64003	-3.02656	-3.91144
C	3.16433	-2.89634	-1.23302
C	2.40634	-2.92830	-2.41955
C	1.75902	-4.11070	-2.77134
H	1.16777	-4.13950	-3.67746
C	1.85363	-5.24380	-1.98016
H	1.34324	-6.15400	-2.26870
C	2.60678	-5.21002	-0.81399
H	2.68153	-6.10353	-0.20939
C	3.27254	-4.04650	-0.42037
C	4.12560	-4.02725	0.84055
H	4.01260	-3.03968	1.29144
C	3.72037	-5.07789	1.87842
H	2.65544	-5.06804	2.11825
H	4.26725	-4.91086	2.80649
H	3.96068	-6.08730	1.53969
C	5.61849	-4.19216	0.52198
H	5.79713	-5.15257	0.03243
H	6.20488	-4.16487	1.44295
H	5.97976	-3.40580	-0.13415
C	2.29505	-1.71276	-3.32193
H	2.88860	-0.91647	-2.88026
C	2.86774	-1.99318	-4.71719
H	3.90672	-2.31785	-4.66094
H	2.82736	-1.08770	-5.32608
H	2.29374	-2.76573	-5.23395
C	0.85258	-1.21620	-3.41908
H	0.21330	-1.95035	-3.90257
H	0.80612	-0.29868	-4.00711
H	0.43200	-1.00158	-2.43894
C	3.10555	0.87763	2.89207
C	2.81504	-0.07503	3.88169
C	2.28459	0.35349	5.09668
H	2.07426	-0.36777	5.87485
C	2.04015	1.69682	5.33632
H	1.64835	2.01581	6.29370
C	2.25562	2.62087	4.32592
H	2.01156	3.66237	4.49594
C	2.75908	2.22968	3.08896
C	2.88500	3.25098	1.97162
H	3.33146	2.75405	1.11410
C	3.78307	4.43464	2.34224
H	3.37685	4.99366	3.18809
H	3.86887	5.12583	1.50067
H	4.78464	4.09614	2.60514
C	1.49407	3.73086	1.54317
H	0.84638	2.88390	1.31626
H	1.56716	4.36880	0.66025
H	1.01195	4.30693	2.33208
C	3.13771	-1.53565	3.64386
H	3.07091	-1.69158	2.56777
C	4.57890	-1.85473	4.05804
H	5.28455	-1.22386	3.51893
H	4.82491	-2.89827	3.85000
H	4.71571	-1.68110	5.12775
C	2.14203	-2.48679	4.31090
H	2.22892	-2.47274	5.39859
H	2.32896	-3.51577	3.99687
H	1.11156	-2.22059	4.06477
C	0.68517	-0.00871	-0.02120
C	1.66274	0.85813	-0.75730
C	1.59409	6.05941	-1.63376
H	2.01894	6.39232	-0.69477
C	0.41814	6.64350	-2.10196
H	-0.06087	7.42414	-1.52455
C	-0.13555	6.22935	-3.30889
H	-1.04491	6.68508	-3.67847
C	0.49741	5.22605	-4.04217
H	0.07549	4.89519	-4.98262
C	1.66502	4.64158	-3.56570
H	2.13863	3.84762	-4.12974
C	2.23438	5.04742	-2.35259
C	3.47420	4.37558	-1.83563
H	3.25766	3.32824	-1.61349

H	3.84290	4.85459	-0.92959
H	4.27154	4.40046	-2.58040
O	-1.47029	0.90778	-0.65131
O	-1.38529	-1.80807	1.49273
C	-0.68520	0.00869	0.02117
C	-1.66276	-0.85817	0.75726
K	0.70696	-3.26524	1.30849
Al	-3.14186	0.23240	-0.10866
O	-4.93154	-0.59110	0.58816
N	-3.86334	1.70742	0.88963
N	-3.83819	-0.51370	-1.72547
C	-5.65091	0.26470	1.42324
C	-5.02902	1.49495	1.60984
C	-5.70078	2.38383	2.45990
H	-5.26702	3.35033	2.67571
C	-6.95558	2.05886	2.98201
C	-7.57810	0.85683	2.63946
H	-8.57638	0.65714	3.00178
C	-6.92242	-0.07058	1.82382
C	-7.49783	-1.38741	1.28630
C	-7.01114	-1.51970	-0.16441
C	-7.74716	-1.99514	-1.25343
H	-8.76580	-2.33074	-1.12041
C	-7.17922	-2.02913	-2.52955
C	-5.88055	-1.56106	-2.74454
H	-5.46696	-1.56976	-3.74359
C	-5.11334	-1.06600	-1.68147
C	-5.72363	-1.11868	-0.43354
C	-6.91911	-2.55962	2.11143
H	-5.83127	-2.55958	2.07833
H	-7.23090	-2.47437	3.15394
H	-7.27524	-3.51172	1.71397
C	-9.02238	-1.41712	1.37655
H	-9.40774	-2.36272	0.99527
H	-9.34454	-1.33153	2.41427
H	-9.47303	-0.60463	0.80627
C	-3.16430	2.89627	1.23308
C	-2.40629	2.92816	2.41960
C	-1.75896	4.11053	2.77145
H	-1.16770	4.13928	3.67757
C	-1.85356	5.24367	1.98033
H	-1.34315	6.15386	2.26891
C	-2.60672	5.20996	0.81417
H	-2.68147	6.10351	0.20962
C	-3.27249	4.04648	0.42049
C	-4.12559	4.02730	-0.84040
H	-4.01259	3.03977	-1.29137
C	-3.72042	5.07804	-1.87821
H	-2.65549	5.06823	-2.11807
H	-4.26732	4.91107	-2.80627
H	-3.96074	6.08741	-1.53938
C	-5.61847	4.19216	-0.52176
H	-5.79712	5.15253	-0.03213
H	-6.20490	4.16492	-1.44271
H	-5.97969	3.40574	0.13432
C	-2.29501	1.71257	3.32192
H	-2.88856	0.91630	2.88022
C	-2.86769	1.99291	4.71720
H	-3.90667	2.31760	4.66096
H	-2.82732	1.08740	5.32604
H	-2.29369	2.76543	5.23400
C	-0.85254	1.21601	3.41905
H	-0.21326	1.95012	3.90258
H	-0.80608	0.29844	4.00702
H	-0.43195	1.00145	2.43889
C	-3.10558	-0.87758	-2.89211
C	-2.81505	0.07512	-3.88168
C	-2.28452	-0.35334	-5.09665
H	-2.07418	0.36795	-5.87479
C	-2.04002	-1.69665	-5.33633
H	-1.64816	-2.01561	-6.29369
C	-2.25551	-2.62075	-4.32597
H	-2.01141	-3.66223	-4.49600
C	-2.75905	-2.22961	-3.08901
C	-2.88497	-3.25095	-1.97170
H	-3.33156	-2.75409	-1.11421
C	-3.78289	-4.43470	-2.34244
H	-3.37653	-4.99364	-3.18827
H	-3.86868	-5.12591	-1.50090
H	-4.78447	-4.09629	-2.60542
C	-1.49403	-3.73070	-1.54314
H	-0.84645	-2.88369	-1.31610
H	-1.56715	-4.36870	-0.66027
H	-1.01176	-4.30667	-2.33203
C	-3.13780	1.53572	-3.64383
H	-3.07101	1.69163	-2.56773
C	-4.57901	1.85473	-4.05800
H	-5.28463	1.22380	-3.51891
H	-4.82508	2.89824	-3.84993
H	-4.71581	1.68111	-5.12772
C	-2.14219	2.48693	-4.31085
H	-2.22907	2.47289	-5.39853
H	-2.32917	3.51589	-3.99679
H	-1.11170	2.22079	-4.06472
C	-1.59400	-6.05950	1.63380
H	-2.01890	-6.39238	0.69482
C	-0.41800	-6.64358	2.10189
H	0.06099	-7.42418	1.52441
C	0.13576	-6.22947	3.30880
H	1.04516	-6.68519	3.67830
C	-0.49718	-5.22622	4.04217
H	-0.07521	-4.89540	4.98261
C	-1.66485	-4.64177	3.56581
H	-2.13844	-3.84786	4.12993
C	-2.23427	-5.04757	2.35272
C	-3.47416	-4.37575	1.83588
H	-3.25767	-3.32840	1.61377
H	-3.84291	-4.85474	0.92985
H	-4.27144	-4.40070	2.58071
C	-7.96343	-2.58529	-3.68966
H	-7.67101	-3.61799	-3.90090
H	-9.03408	-2.58207	-3.48317
H	-7.78974	-2.00811	-4.59934
C	-7.64003	3.02661	3.91143
H	-8.71414	2.84384	3.95393
H	-7.24902	2.93462	4.92877
H	-7.48187	4.05950	3.59630
H	7.67103	3.61818	3.90065
H	7.78960	2.00836	4.59924
H	9.03403	2.58213	3.48306
H	8.71413	-2.84373	-3.95399
H	7.48195	-4.05945	-3.59627
H	7.24899	-2.93463	-4.92878

F_TZVP

246

SCF Done: E(RB3LYP) = -7420.48092645 A.U.

K	0.15280	-1.87784	2.22393
K	0.55597	1.93138	2.81454
Al	-3.10522	0.03652	-0.22364
O	-1.48073	0.10192	-1.16496
O	-4.93858	-0.06659	0.80712
O	-1.27250	0.25833	2.41136
N	-3.93038	1.75070	-0.68668
N	-3.76997	-1.73948	-0.74945
C	-0.73552	0.16649	0.05616
C	-1.62677	0.16899	1.10529
C	-5.68900	-1.21004	0.53414
C	-5.04878	-2.09317	-0.32372
C	-5.71026	1.09428	0.72118
C	-6.97948	-1.32146	1.00236
C	-5.13529	2.07425	-0.08118
C	-7.51678	-0.17466	1.86576
C	-6.98646	1.11743	1.23417
C	-3.10696	-2.85069	-1.34996
C	-7.70793	2.30163	1.05810
H	-8.71105	2.39291	1.44875
C	-3.36273	2.82362	-1.42324
C	-7.15175	3.36802	0.35212
C	-2.62762	-3.90341	-0.52706
C	-2.75956	3.90991	-0.74968
C	-2.93965	-1.33220	3.60618
H	-3.60363	-1.41210	2.73920
H	-2.18635	-2.12070	3.45399
C	-5.79376	-3.22467	-0.68816
H	-5.36192	-3.96323	-1.34816
C	-9.04324	-0.18270	1.93471
H	-9.49245	-0.08016	0.94656
H	-9.39867	0.63373	2.56310
H	-9.39785	-1.11078	2.38267
C	-5.88914	3.24713	-0.23125
H	-5.51060	4.05437	-0.84173
C	-7.08989	-3.41644	-0.21003
C	-3.41175	1.32147	3.51858
H	-3.65146	1.47804	2.46388
H	-4.34345	0.95592	3.95939
C	-3.06187	-2.99268	-2.75776
C	-7.68284	-2.46685	0.62303
H	-8.69874	-2.61868	0.95813
C	-2.25011	4.97315	-1.49573
H	-1.78408	5.80395	-0.98165
C	-3.48961	2.85949	-2.83085
C	-1.31984	0.37939	4.95224
H	-0.93315	1.40790	5.01412
H	-1.97480	0.30833	5.82661
C	-2.59912	-3.80029	0.99017
H	-2.59699	-2.73813	1.23468
C	-7.91299	4.65894	0.20336
C	-2.34215	4.99795	-2.87794
H	-1.94658	5.83481	-3.43928
C	-2.96643	3.94587	-3.53476

H	-3.06535	3.98481	-4.61057	C	3.72340	-1.71131	-4.86538
C	-2.57658	-4.18364	-3.30517	H	3.65116	1.47860	-2.46352
H	-2.55077	-4.30845	-4.37711	H	4.34371	0.95606	-3.95861
C	-2.67669	3.97640	0.76416	C	3.12209	2.67886	-4.16510
H	-3.11690	3.06827	1.16511	H	0.93326	1.40848	-5.01395
C	-2.16762	-5.07589	-1.12574	H	1.97491	0.30895	-5.82647
H	-1.81627	-5.88809	-0.50460	C	0.14972	-0.58216	-5.16035
C	-7.83914	-4.66756	-0.58715	O	4.93857	-0.06643	-0.80708
C	-6.94787	-0.32687	3.29279	C	6.98642	1.11767	-1.23405
H	-7.30549	-1.25552	3.74073	C	7.15165	3.36821	-0.35184
H	-7.26443	0.51080	3.91656	H	5.51051	4.05442	0.84210
H	-5.86215	-0.35035	3.28059	C	2.24992	4.97306	1.49594
C	-3.12154	2.67862	4.16496	C	2.67694	3.97645	-0.76393
H	-2.18163	3.11499	3.81459	C	2.96595	3.94568	3.53503
H	-3.03615	2.59357	5.25055	C	4.25044	1.77235	3.56582
H	-3.90457	3.41346	3.95830	C	6.97947	-1.32125	-1.00252
C	-3.72379	-1.71163	4.86525	H	5.36198	-3.96335	1.34768
H	-3.06274	-1.81477	5.72964	C	7.08991	-3.41640	0.20956
H	-4.26622	-2.65557	4.75567	C	2.59919	-3.80012	-0.99041
H	-4.45897	-0.94588	5.12121	C	2.16770	-5.07596	1.12535
C	-2.14753	-5.22685	-2.50087	C	2.57681	-4.18397	3.30486
H	-1.79156	-6.14731	-2.94552	C	3.56428	-1.89552	3.68538
C	-3.56371	-1.89503	-3.68550	H	3.06214	-1.81448	-5.72961
H	-3.17485	-0.94998	-3.29725	H	4.26584	-2.65525	-4.75590
C	-1.34225	-4.45259	1.57920	H	4.45853	-0.94558	-5.12156
H	-1.39226	-5.54061	1.52963	H	2.18206	3.11541	-3.81530
H	-1.25242	-4.20643	2.63957	H	3.03720	2.59355	-5.25072
H	-0.42637	-4.16914	1.06017	H	3.90511	3.41366	-3.95826
C	-3.45043	5.17540	1.32728	H	-0.34155	-0.46557	-6.12999
H	-3.03254	6.11990	0.97112	H	-0.64132	-0.44292	-4.41385
H	-3.39727	5.17378	2.41639	H	0.47516	-1.62589	-5.09848
H	-4.49991	5.13561	1.04239	C	7.51675	-0.17434	-1.86579
C	-4.25079	1.77243	-3.56546	C	7.70785	2.30189	-1.05791
H	-4.13848	0.85522	-2.98951	C	7.91285	4.65915	-0.20303
C	-0.14964	-0.58275	5.16036	H	1.78393	5.80386	0.98183
H	0.34163	-0.46628	6.13001	C	2.34172	4.99777	2.87817
H	0.64140	-0.44338	4.41388	H	3.11779	3.06864	-1.16487
H	-0.47506	-1.62648	5.09835	C	3.45003	5.17594	-1.32687
C	-1.22247	4.02611	1.22668	C	1.22276	4.02519	-1.22669
H	-0.68490	3.15621	0.85113	H	3.06469	3.98455	4.61085
H	-1.18084	4.05181	2.31596	H	4.13817	0.85510	2.98992
H	-0.71038	4.91599	0.86519	C	3.73585	1.51890	4.98290
B	-2.26455	0.16040	3.62346	C	5.75160	2.09465	3.60391
C	-3.07039	-2.06017	-5.12594	C	7.68285	-2.46670	-0.62337
H	-1.99510	-2.21960	-5.18848	C	7.83918	-4.66758	0.58649
H	-3.30409	-1.16964	-5.70485	H	2.59702	-2.73793	-1.23479
H	-3.56388	-2.90157	-5.61625	C	1.34233	-4.45241	-1.57951
C	-3.73627	1.51886	-4.98255	C	3.81600	-4.42936	-1.68672
H	-3.87107	2.38753	-5.62819	H	1.81633	-5.88808	0.50413
H	-4.29331	0.70095	-5.43606	C	2.14767	-5.22707	2.50046
H	-2.67805	1.24700	-5.00373	H	2.55114	-4.30894	4.37678
C	-3.81587	-4.42969	1.68645	H	3.17569	-0.95031	3.29724
H	-4.74442	-3.93519	1.42610	C	3.07101	-2.06063	5.12585
H	-3.68924	-4.36418	2.76830	C	5.09657	-1.79601	3.69393
H	-3.90259	-5.48499	1.41516	C	9.04321	-0.18234	-1.93474
C	-5.09596	-1.79502	-3.69412	C	6.94785	-0.32639	-3.29284
H	-5.53431	-2.75563	-3.97186	H	8.71095	2.39323	-1.44859
H	-5.41867	-1.05677	-4.42954	H	1.94601	5.83457	3.43950
H	-5.49544	-1.50965	-2.72674	H	3.03140	6.12020	-0.97094
C	-5.75196	2.09467	-3.60352	H	3.39726	5.17420	-2.41599
H	-6.16017	2.20449	-2.60151	H	4.49945	5.13689	-1.04160
H	-6.29842	1.29618	-4.10899	H	0.68584	3.15479	-0.85135
H	-5.92820	3.02593	-4.14745	H	1.18132	4.05099	-2.31597
C	0.73553	0.16651	-0.05605	H	0.70997	4.91464	-0.86514
O	1.48075	0.10179	1.16506	H	3.87056	2.38765	5.62846
C	1.62677	0.16918	-1.10517	H	4.29290	0.70107	5.43654
Al	3.10524	0.03655	0.22373	H	2.67764	1.24699	5.00403
O	1.27248	0.25873	-2.41123	H	6.15984	2.20442	2.60192
N	3.93037	1.75069	0.68694	H	6.29807	1.29621	4.10945
N	3.77004	-1.73951	0.74934	H	5.92778	3.02595	4.14780
K	-0.15268	-1.87759	-2.22396	H	8.69874	-2.61848	-0.95852
K	-0.55636	1.93143	-2.81470	H	1.39237	-5.54043	-1.53002
B	2.26457	0.16085	-3.62332	H	1.25249	-4.20615	-2.63985
C	5.13526	2.07433	0.08144	H	0.42645	-4.16902	-1.06044
C	3.36263	2.82357	1.42350	H	4.74452	-3.93488	-1.42620
C	5.04882	-2.09315	0.32351	H	3.68946	-4.36366	-2.76857
C	3.10707	-2.85078	1.34978	H	3.90274	-5.48471	-1.41561
C	2.93957	-1.33181	-3.60614	H	1.79175	-6.14758	2.94502
C	3.41186	1.32182	-3.51828	H	1.99567	-2.21968	5.18845
C	1.31992	0.37997	-4.95212	H	3.30507	-1.17023	5.70482
C	5.71023	1.09445	-0.72102	H	3.56424	-2.90226	5.61604
C	5.88906	3.24724	0.23156	H	5.53462	-2.75680	3.97151
C	2.75956	3.90990	0.74991	H	5.41956	-1.05798	4.42945
C	3.48929	2.85936	2.83114	H	5.49609	-1.51063	2.72658
C	5.68900	-1.20990	-0.53426	H	9.49242	-0.07990	-0.94658
C	5.79381	-3.22470	0.68777	H	9.39863	0.63417	-2.56304
C	2.62769	-3.90340	0.52679	H	9.39784	-1.11037	-2.38280
C	3.06208	-2.99295	2.75756	H	7.30549	-1.25499	-3.74088
H	3.60374	-1.41172	-2.73930	H	7.26438	0.51136	-3.91651
H	2.18626	-2.12027	-3.45378	H	5.86213	-0.34991	-3.28063

```
H          7.57658          5.39833          -0.93603
H          7.76656          5.09502           0.78690
H          8.98215          4.50941          -0.35504
H          8.91573         -4.53384           0.47646
H          7.63538         -4.95634           1.61902
H          7.54431         -5.50715          -0.04968
H         -7.76677          5.09482          -0.78658
H         -8.98228          4.50917           0.35543
H         -7.57670          5.39813           0.93634
H         -7.63526         -4.95623          -1.61969
H         -7.54434         -5.50720           0.04896
H         -8.91569         -4.53382          -0.47720
```

Orca Outputs

G_6-31G*

```
61
N         -3.96017         -1.67030           0.70935
N         -3.96056          1.47826          -0.48248
C         -5.21931         -2.17610           0.46482
C         -3.18312         -2.27519           1.71826
C         -5.21317          1.69831          -1.01514
C         -3.18535          2.59599          -0.11292
C         -5.92817         -1.60493          -0.60545
C         -5.94907         -3.12808           1.20869
C         -2.88076         -3.64917           1.68895
C         -2.65390         -1.50269           2.76433
C         -5.92275          0.56166          -1.43819
C         -5.93774          2.90640          -1.10308
C         -2.87938          3.61810          -1.03041
C         -2.66097          2.69420           1.18588
O         -5.21284         -0.63467          -1.31102
C         -7.26529         -1.78898          -0.89441
C         -7.28705         -3.41151           0.90822
H         -5.47911         -3.61733           2.05551
C         -2.09206         -4.22950           2.68019
C         -1.85650         -2.08326           3.74746
C         -7.25846          0.51055          -1.78204
H         -5.46771          3.83387          -0.79327
C         -7.27195          2.92242          -1.52818
C         -2.09153          4.70435          -0.65494
C         -1.86416          3.77490           1.55479
C         -7.86082         -0.88909          -1.99077
C         -7.95424         -2.73627          -0.12339
C         -8.01962         -4.46083           1.71514
H         -1.86794         -5.29350           2.63257
C         -1.57310         -3.45130           3.71782
H         -1.45419         -1.46164           4.54477
C         -7.94168          1.73344          -1.84830
C         -7.99731          4.24553          -1.63427
H         -1.86577          5.47991          -1.38444
C         -1.57671          4.79195           0.64068
H         -1.46579          3.82323           2.56608
C         -9.39214         -0.86019          -1.92712
C         -7.42524         -1.42577          -3.37833
H         -9.00438         -2.94247          -0.30545
H         -7.69204         -4.46226           2.76169
H         -9.10314         -4.29262           1.70045
H         -7.84147         -5.47204           1.32144
H         -0.95062         -3.90260           4.48705
H         -8.98987          1.76864          -2.12815
H         -7.70399          4.92906          -0.82836
H         -7.77477          4.75452          -2.58321
H         -9.08432          4.11104          -1.58444
H         -0.95469          5.63537           0.93100
H         -9.74713         -0.48654          -0.96088
H         -9.80153         -1.86452          -2.08137
H         -9.79815         -0.21842          -2.71669
H         -7.80984         -0.77566          -4.17405
H         -7.81608         -2.43918          -3.53328
H         -6.33565         -1.46135          -3.46024
H         -3.26665         -4.24984           0.87011
H         -2.87174         -0.44076           2.78474
H         -2.88222          1.90348           1.89375
H         -3.26081          3.54089          -2.04451
C         -1.17429         -0.37692          -0.62611
Al        -3.22772         -0.33980          -0.53067
O         -1.60399          0.05002           0.49063
```

H_6-31G*

```
61
Al        -2.77259         -0.33947          -0.37731
N         -3.62252         -1.74111           0.79851
N         -3.61133          1.48384          -0.19935
C         -4.90664         -2.16743           0.57465
C         -2.92460         -2.24305           1.90993
C         -4.83365          1.74136          -0.76441
C         -2.83290          2.59374           0.19306
C         -5.59781         -1.54525          -0.47911
C         -5.64315         -3.16329           1.25365
C         -1.69900         -2.90651           1.72878
C         -3.38914         -2.04943           3.22574
C         -5.54886          0.63155          -1.24780
C         -5.52231          2.97144          -0.85643
C         -2.24838          3.44643          -0.75907
C         -2.59752          2.85066           1.55335
O         -4.85638         -0.57653          -1.15484
C         -6.93260         -1.70677          -0.78874
C         -6.98221         -3.41846           0.93074
H         -5.17318         -3.71662           2.06098
C         -0.95292         -3.33864           2.82595
C         -2.65323         -2.50377           4.31584
C         -6.87869          0.61779          -1.61436
H         -5.03361          3.88127          -0.52091
C         -6.84547          3.02557          -1.31320
C         -1.45833          4.52579          -0.36105
C         -1.80332          3.92556           1.94929
C         -7.50612         -0.76447          -1.86197
C         -7.63781         -2.68218          -0.06566
C         -7.72493         -4.51266           1.66607
H          0.00083         -3.83621           2.66209
C         -1.42581         -3.14785           4.12454
H         -3.03028         -2.34101           5.32391
C         -7.53575          1.85753          -1.66624
C         -7.53828          4.36682          -1.41562
H         -1.01112          5.17194          -1.11407
C         -1.22927          4.77000           0.99410
H         -1.63330          4.10668           3.00857
C         -9.03718         -0.70881          -1.80343
C         -7.07511         -1.27340          -3.26134
H         -8.68934         -2.86728          -0.26218
H         -7.42850         -4.56077           2.72106
H         -8.80951         -4.35532           1.62710
H         -7.52359         -5.50248           1.23122
H         -0.84712         -3.49277           4.97837
H         -8.57806          1.92154          -1.96294
H         -7.27783          5.01898          -0.57279
H         -7.25321          4.90126          -2.33347
H         -8.62887          4.25355          -1.43006
H         -0.60753          5.60702           1.30395
H         -9.38873         -0.35417          -0.82882
H         -9.46444         -1.70109          -1.98522
H         -9.42859         -0.03891          -2.57693
H         -7.44289         -0.59522          -4.04159
H         -7.48456         -2.27478          -3.44461
H         -5.98594         -1.32740          -3.33844
H         -1.34319         -3.06114           0.71475
H         -4.32960         -1.52563           3.37156
H         -3.04545          2.18706           2.28631
H         -2.42715          3.24494          -1.81133
C         -1.28007          0.13705           1.12823
O         -0.14068          0.23592           1.34752
```

I_6-31G*61

```
63
N         -0.93814         -0.73183           1.46388
N         -1.01861          2.41496           0.24694
C         -2.18233         -1.26740           1.18850
C         -0.10131         -1.31398           2.42568
C         -2.21910          2.58094          -0.40517
C         -0.28345          3.54537           0.65179
C         -2.84007         -0.73132           0.07010
C         -2.94151         -2.20251           1.92178
C          0.05049         -2.70706           2.57660
C          0.69990         -0.47766           3.22763
C         -2.84783          1.40403          -0.84397
C         -2.96899          3.75894          -0.60288
C          0.07607          4.56652          -0.24452
C          0.14554          3.64609           1.98538
O         -2.10531          0.24147          -0.61069
C         -4.14666         -0.96600          -0.30856
C         -4.25358         -2.52350           1.54917
H         -2.52123         -2.65065           2.81572
C          0.94036         -3.23377           3.50979
C          1.60198         -1.01082           4.14475
C         -4.14728          1.29018          -1.29255
H         -2.55404          4.71328          -0.29554
C         -4.26276          3.70912          -1.13708
C          0.83462          5.65294           0.18368
C          0.92169          4.72459           2.40521
C         -4.68330         -0.13690          -1.48730
C         -4.86159         -1.90361           0.44954
C         -5.01849         -3.55412           2.34999
H          1.03566         -4.31414           3.60277
C          1.72327         -2.39267           4.30482
H          2.21181         -0.33639           4.74183
C         -4.86333          2.48425          -1.45981
C         -5.01931          4.99943          -1.36261
H          1.10830          6.42822          -0.52907
C          1.26692          5.73869           1.51033
H          1.25287          4.77466           3.44027
C         -6.21453         -0.16332          -1.54752
C         -4.11612         -0.71458          -2.80948
H         -5.89129         -2.14355           0.20362
```

H	-4.79490	-3.47650	3.42081
H	-6.10097	-3.43740	2.22186
H	-4.76134	-4.57769	2.04183
H	2.42160	-2.80738	5.02771
H	-5.88719	2.47003	-1.81973
H	-4.81273	5.72914	-0.57047
H	-4.73667	5.47063	-2.31481
H	-6.10196	4.82911	-1.39214
H	1.87006	6.58163	1.83901
H	-6.66033	0.23491	-0.62990
H	-6.57561	-1.18740	-1.69197
H	-6.57530	0.43148	-2.39353
H	-4.45713	-0.11385	-3.66153
H	-4.45691	-1.74785	-2.94949
H	-3.02296	-0.71090	-2.80345
H	-0.51839	-3.37319	1.93577
H	0.60938	0.59739	3.11404
H	-0.14159	2.86408	2.68251
H	-0.22729	4.48087	-1.28357
Al	-0.20311	0.64110	0.22994
C	1.01328	0.98468	-1.36549
C	1.69174	1.37846	0.32365
O	1.63185	0.76798	-2.36623
O	2.84556	1.43844	0.62808

J_6-31G*61

63

C	-1.88009	-0.63602	-1.57597
O	-0.69777	-0.68689	-1.70692
C	-1.25985	0.22917	1.13450
O	-0.11404	0.12543	0.84110
Al	-2.99136	-0.10286	0.10986
O	-4.82277	-0.43831	-0.86032
N	-3.72879	-1.56896	1.14882
N	-3.71834	1.69605	0.00729
C	-5.56379	0.72110	-1.08449
C	-4.92303	1.88699	-0.63948
C	-5.57455	-1.47570	-0.31002
C	-6.84618	0.62607	-1.58164
C	-4.93950	-2.11063	0.76744
C	-7.37400	-0.78597	-1.87775
C	-6.85832	-1.70220	-0.75777
C	-2.93000	2.82648	0.32348
C	-7.56392	-2.74053	-0.12983
H	-8.57783	-2.98137	-0.43299
C	-2.95879	-2.25602	2.11674
C	-6.96503	-3.46933	0.90711
C	-2.57500	3.77176	-0.65341
C	-2.58410	-3.59739	1.93596
C	-5.65085	3.07632	-0.83995
H	-5.22485	4.02319	-0.52427
C	-8.90401	-0.81080	-1.96913
H	-9.36669	-0.47288	-1.03577
H	-9.25971	-1.82399	-2.18489
H	-9.25237	-0.16448	-2.78189
C	-5.67548	-3.15657	1.35773
H	-5.25283	-3.70048	2.19635
C	-6.93612	3.04700	-1.39760
C	-2.45323	3.00589	1.63198
C	-7.54170	1.83314	-1.75070
H	-8.55248	1.83687	-2.14605
C	-1.81440	-4.25840	2.89363
H	-1.52917	-5.29567	2.73045
C	-2.52721	-1.58927	3.27415
C	-7.71676	-4.61149	1.55461
C	-1.39461	-3.58933	4.04496
H	-0.78940	-4.10220	4.78902
C	-1.75215	-2.25133	4.22614
H	-1.42688	-1.71630	5.11566
C	-1.64925	4.09998	1.95046
H	-1.28665	4.21625	2.96945
C	-1.77998	4.87034	-0.32828
H	-1.51183	5.58589	-1.10290
C	-7.67420	4.34867	-1.61969
C	-6.77418	-1.26651	-3.22505
H	-7.09819	-0.60869	-4.04087
H	-7.10507	-2.28913	-3.44404
H	-5.68084	-1.25897	-3.19162
C	-1.31149	5.04151	0.97621
H	-0.68434	5.89358	1.22817
H	-7.56418	-4.62765	2.64068
H	-8.79426	-4.53836	1.36639
H	-7.38211	-5.58533	1.16961
H	-7.50472	5.05164	-0.79504
H	-7.34163	4.84885	-2.54047
H	-8.75475	4.18630	-1.70828
H	-2.79460	-0.54632	3.40103
H	-2.90040	-4.11059	1.03230
H	-2.70305	2.26345	2.38147
H	-2.92351	3.62749	-1.67205

R_SVP

71

Al	-4.63824	-2.90227	-0.51570
N	-4.06255	-1.70577	1.05071
N	-3.62721	-2.34990	-2.20741
C	-4.49774	-0.41357	1.15623
C	-3.10954	-2.13485	1.99844
C	-3.83413	-1.12542	-2.79267
C	-3.04871	-3.35593	-3.00666
C	-5.19410	0.11102	0.05267
C	-4.28468	0.49344	2.22368
C	-1.77127	-1.67778	1.90684
C	-3.49155	-3.01371	3.03664
C	-4.75179	-0.26737	-2.16406
C	-3.20612	-0.59427	-3.94429
C	-3.72742	-3.83499	-4.15756
C	-1.80434	-3.91917	-2.63909
O	-5.32118	-0.79137	-1.00487
C	-5.47185	1.45398	-0.14763
C	-4.65105	1.84379	2.11227
H	-3.76790	0.14634	3.12133
C	-0.82950	-2.14248	2.83474
C	-2.52245	-3.45725	3.94631
C	-4.98786	1.05500	-2.50840
H	-2.48601	-1.21055	-4.48781
C	-3.45544	0.71918	-4.37151
C	-3.13266	-4.83820	-4.93295
C	-1.24691	-4.93005	-3.43354
C	-5.89233	1.86902	-1.56715
C	-5.21716	2.33035	0.92212
C	-4.41788	2.78539	3.27187
H	0.20642	-1.79664	2.75934
C	-1.19556	-3.03241	3.84651
H	-2.82032	-4.13486	4.75232
C	-4.32908	1.54765	-3.64731
C	-2.77780	1.24607	-5.61625
H	-3.65842	-5.20090	-5.82187
C	-1.89759	-5.38607	-4.58124
H	-0.27880	-5.35425	-3.14996
C	-5.73090	3.37736	-1.78932
C	-7.37076	1.48259	-1.81102
H	-5.42633	3.39690	0.82663
H	-3.49921	2.52980	3.82426
H	-4.33329	3.83014	2.93202
H	-5.25103	2.74575	3.99680
H	-0.44913	-3.38732	4.56279
H	-4.47623	2.57994	-3.96882
H	-1.75744	0.84411	-5.72384
H	-3.33349	0.96141	-6.52807
H	-2.71273	2.34584	-5.60530
H	-1.44737	-6.17023	-5.19633
H	-4.69209	3.70341	-1.62933
H	-6.37892	3.94276	-1.10252
H	-6.02403	3.65321	-2.81366
H	-7.66600	1.73715	-2.84219
H	-8.02638	2.03026	-1.11431
H	-7.53002	0.40538	-1.66167
C	-1.07338	-3.39981	-1.42952
H	-0.88871	-2.31779	-1.51664
H	-1.65591	-3.53834	-0.50394
H	-0.10644	-3.90920	-1.30003
C	-5.07725	-3.28342	-4.53450
H	-5.57559	-3.92976	-5.27262
H	-5.72316	-3.19192	-3.64679
H	-4.99925	-2.27082	-4.96576
C	-1.38187	-0.67649	0.85083
H	-1.67437	0.34477	1.15080
H	-0.29489	-0.67977	0.67762
H	-1.89776	-0.87862	-0.09742
C	-4.93787	-3.40950	3.17908
H	-5.57963	-2.51571	3.25876
H	-5.29227	-3.96172	2.29317
H	-5.09417	-4.03307	4.07263

I1_SVP

73

Al	-2.78280	-0.29669	-0.42315
N	-3.62072	-1.70090	0.76172
N	-3.60479	1.54899	-0.37544
C	-4.86277	-2.20083	0.48408
C	-2.86971	-2.23818	1.82756
C	-4.85615	1.76688	-0.87917
C	-2.81826	2.67867	-0.04834
C	-5.57069	-1.56537	-0.55603
C	-5.55140	-3.26438	1.10900
C	-1.67292	-2.93718	1.54219
C	-3.25409	-2.00420	3.17199
C	-5.57145	0.62790	-1.30186
C	-5.55990	2.98762	-0.99165
C	-2.09708	3.35133	-1.06311
C	-2.74037	3.12077	1.29238

O	-4.85618	-0.55418	-1.17969
C	-6.90126	-1.76022	-0.87201
C	-6.88436	-3.55911	0.77169
H	-5.05178	-3.83747	1.89366
C	-0.88923	-3.41033	2.60299
C	-2.44839	-2.50127	4.20447
C	-6.90549	0.58754	-1.65692
H	-5.06566	3.91681	-0.69841
C	-6.90003	3.01287	-1.41731
C	-1.28862	4.44206	-0.71570
C	-1.92078	4.21439	1.60299
C	-7.51203	-0.80460	-1.91439
C	-7.56820	-2.79618	-0.18908
C	-7.58089	-4.71016	1.46113
H	0.03854	-3.94636	2.37987
C	-1.27251	-3.20311	3.92894
H	-2.74175	-2.31473	5.24236
C	-7.57949	1.82297	-1.72645
C	-7.60911	4.34264	-1.53625
H	-0.72490	4.95742	-1.49954
C	-1.19362	4.87259	0.60928
H	-1.85846	4.55383	2.64155
C	-9.04130	-0.77900	-1.82690
C	-7.09323	-1.27699	-3.32960
H	-8.61677	-3.01373	-0.39770
H	-7.40988	-4.69255	2.55045
H	-8.66777	-4.68867	1.28651
H	-7.20545	-5.68279	1.09601
H	-0.64886	-3.57397	4.74724
H	-8.63086	1.86594	-2.01462
H	-7.39871	4.99050	-0.66926
H	-7.27792	4.89508	-2.43385
H	-8.70027	4.21535	-1.61065
H	-0.55567	5.72287	0.86669
H	-9.38506	-0.45020	-0.83423
H	-9.45733	-1.77841	-2.02379
H	-9.46224	-0.09807	-2.58177
H	-7.48476	-0.58741	-4.09500
H	-7.48922	-2.28611	-3.52858
H	-5.99825	-1.31155	-3.42590
C	-1.25604	0.20644	0.96942
O	-0.10618	0.25938	1.14543
C	-1.26042	-3.16887	0.11064
H	-0.16678	-3.24825	0.01487
H	-1.70436	-4.09379	-0.28651
H	-1.61760	-2.34683	-0.54395
C	-4.48860	-1.19991	3.47999
H	-4.56431	-0.33273	2.80743
H	-5.40830	-1.78942	3.32665
H	-4.48065	-0.84489	4.52161
C	-3.52273	2.41074	2.36268
H	-4.57518	2.27364	2.06961
H	-3.10628	1.40309	2.53124
H	-3.48891	2.96299	3.31401
C	-2.22060	2.90204	-2.49596
H	-1.52146	3.44999	-3.14559
H	-2.02138	1.82215	-2.58792
H	-3.24382	3.06644	-2.87589

I3_SVP

73

Al	-2.78280	-0.29669	-0.42315
N	-3.62072	-1.70090	0.76172
N	-3.60479	1.54899	-0.37544
C	-4.86277	-2.20083	0.48408
C	-2.86971	-2.23818	1.82756
C	-4.85615	1.76688	-0.87917
C	-2.81826	2.67867	-0.04834
C	-5.57069	-1.56537	-0.55603
C	-5.55140	-3.26438	1.10900
C	-1.67292	-2.93718	1.54219
C	-3.25409	-2.00420	3.17199
C	-5.57145	0.62790	-1.30186
C	-5.55990	2.98762	-0.99165
C	-2.09708	3.35133	-1.06311
C	-2.74037	3.12077	1.29238
O	-4.85618	-0.55418	-1.17969
C	-6.90126	-1.76022	-0.87201
C	-6.88436	-3.55911	0.77169
H	-5.05178	-3.83747	1.89366
C	-0.88923	-3.41033	2.60299
C	-2.44839	-2.50127	4.20447
C	-6.90549	0.58754	-1.65692
H	-5.06566	3.91681	-0.69841
C	-6.90003	3.01287	-1.41731
C	-1.28862	4.44206	-0.71570
C	-1.92078	4.21439	1.60299
C	-7.51203	-0.80460	-1.91439
C	-7.56820	-2.79618	-0.18908
C	-7.58089	-4.71016	1.46113
H	0.03854	-3.94636	2.37987
C	-1.27251	-3.20311	3.92894
H	-2.74175	-2.31473	5.24236
C	-7.57949	1.82297	-1.72645
C	-7.60911	4.34264	-1.53625
H	-0.72490	4.95742	-1.49954
C	-1.19362	4.87259	0.60928
H	-1.85846	4.55383	2.64155
C	-9.04130	-0.77900	-1.82690
C	-7.09323	-1.27699	-3.32960
H	-8.61677	-3.01373	-0.39770
H	-7.40988	-4.69255	2.55045
H	-8.66777	-4.68867	1.28651
H	-7.20545	-5.68279	1.09601
H	-0.64886	-3.57397	4.74724
H	-8.63086	1.86594	-2.01462
H	-7.39871	4.99050	-0.66926
H	-7.27792	4.89508	-2.43385
H	-8.70027	4.21535	-1.61065
H	-0.55567	5.72287	0.86669
H	-9.38506	-0.45020	-0.83423
H	-9.45733	-1.77841	-2.02379
H	-9.46224	-0.09807	-2.58177
H	-7.48476	-0.58741	-4.09500
H	-7.48922	-2.28611	-3.52858
H	-5.99825	-1.31155	-3.42590
C	-1.25604	0.20644	0.96942
O	-0.10618	0.25938	1.14543
C	-1.26042	-3.16887	0.11064
H	-0.16678	-3.24825	0.01487
H	-1.70436	-4.09379	-0.28651
H	-1.61760	-2.34683	-0.54395
C	-4.48860	-1.19991	3.47999
H	-4.56431	-0.33273	2.80743
H	-5.40830	-1.78942	3.32665
H	-4.48065	-0.84489	4.52161
C	-3.52273	2.41074	2.36268
H	-4.57518	2.27364	2.06961
H	-3.10628	1.40309	2.53124
H	-3.48891	2.96299	3.31401
C	-2.22060	2.90204	-2.49596
H	-1.52146	3.44999	-3.14559
H	-2.02138	1.82215	-2.58792
H	-3.24382	3.06644	-2.87589

P_SVP

150

Al	3.02089	-0.23340	-0.32974
O	4.94947	0.50882	-0.82927
O	1.49041	-1.03853	0.22803
O	1.06923	1.68206	-1.89844
N	3.82565	-1.71824	-1.33035
N	3.64499	0.55398	1.38040
C	5.74372	-0.37253	-1.54955
C	5.09626	-1.58854	-1.82930
C	5.86392	-2.52335	-2.55542
H	5.42087	-3.48403	-2.82643
C	7.19967	-2.24702	-2.89677
C	7.80443	-1.03945	-2.51314
H	8.85162	-0.86214	-2.76289
C	7.06485	-0.06909	-1.81015
C	7.58113	1.28628	-1.30230
C	6.93529	1.51853	0.07326
C	7.54946	2.10444	1.19611
H	8.58235	2.45194	1.14281
C	6.83621	2.24720	2.39786
C	5.51845	1.77372	2.51065
H	4.98744	1.85762	3.46168
C	4.87463	1.15414	1.41862
C	5.62177	1.11886	0.22654
C	7.48631	2.90597	3.59314
C	7.09755	2.39554	-2.27059
H	6.00069	2.40145	-2.34893
H	7.51372	2.23219	-3.27817
H	7.42191	3.38550	-1.91047
C	9.11049	1.31841	-1.22590
H	9.46137	2.29848	-0.86921
H	9.55127	1.15834	-2.22151
H	9.49878	0.54377	-0.54722
C	7.98714	-3.27805	-3.67257
C	3.07688	-2.82802	-1.80220
C	2.56851	-2.81048	-3.12074
C	1.88450	-3.93577	-3.59719
H	1.49486	-3.92700	-4.61921
C	1.67765	-5.05004	-2.78220
H	1.13268	-5.91770	-3.16527
C	2.16171	-5.04646	-1.47303
H	1.99971	-5.91479	-0.82696
C	2.87097	-3.94717	-0.97033
C	2.70272	0.89763	2.38532
C	2.22233	-0.07653	3.28595
C	1.28925	0.30027	4.26234

H	0.91431	-0.45974	4.95446
C	0.83177	1.61251	4.35472
H	0.10551	1.89062	5.12186
C	1.28446	2.56389	3.43986
H	0.89398	3.58311	3.47570
C	2.20404	2.22421	2.44254
C	0.64338	-0.07171	-0.21971
C	1.46182	0.82425	-1.11897
O	-1.49048	1.03861	-0.22803
O	-1.06928	-1.68204	1.89837
C	-0.64343	0.07176	0.21967
C	-1.46188	-0.82422	1.11890
Al	-3.02094	0.23332	0.32954
O	-4.94951	-0.50880	0.82922
N	-3.82573	1.71822	1.33029
N	-3.64501	-0.55397	-1.38050
C	-5.74387	0.37261	1.54929
C	-5.09641	1.58857	1.82913
C	-5.86446	2.52376	2.55437
H	-5.42160	3.48462	2.82505
C	-7.20012	2.24726	2.89596
C	-7.80462	1.03938	2.51291
H	-8.85189	0.86214	2.76237
C	-7.06500	0.06910	1.80985
C	-7.58106	-1.28645	1.30228
C	-6.93524	-1.51873	-0.07327
C	-7.54930	-2.10499	-1.19600
H	-8.58213	-2.45266	-1.14262
C	-6.83617	-2.24744	-2.39785
C	-5.51853	-1.77367	-2.51073
H	-4.98766	-1.85728	-3.46187
C	-4.87457	-1.15436	-1.41863
C	-5.62175	-1.11900	-0.22657
C	-7.09718	-2.39546	2.27071
H	-6.00032	-2.40113	2.34898
H	-7.51332	-2.23205	3.27829
H	-7.42135	-3.38554	1.91076
C	-9.11042	-1.31890	1.22600
H	-9.46113	-2.29909	0.86947
H	-9.55116	-1.15877	2.22161
H	-9.49891	-0.54443	0.54724
C	-3.07691	2.82778	1.80257
C	-2.56933	2.81010	3.12143
C	-1.88573	3.93537	3.59849
H	-1.49628	3.92627	4.62058
C	-1.67857	5.04984	2.78385
H	-1.13358	5.91733	3.16727
C	-2.16241	5.04670	1.47461
H	-2.00019	5.91518	0.82881
C	-2.87115	3.94737	0.97124
C	-2.70295	-0.89716	-2.38581
C	-2.22307	0.07730	-3.28640
C	-1.29004	-0.29902	-4.26302
H	-0.91524	0.46131	-4.95487
C	-0.83183	-1.61101	-4.35548
H	-0.10542	-1.88868	-5.12264
C	-1.28394	-2.56267	-3.44062
H	-0.89279	-3.58167	-3.47639
C	-2.20363	-2.22350	-2.44320
C	-7.48620	-2.90644	-3.59305
H	-6.97969	-3.85250	-3.85303
H	-8.54639	-3.13517	-3.40331
H	-7.43151	-2.26235	-4.48722
C	-7.98808	3.27887	3.67048
H	-9.03592	2.96977	3.80731
H	-7.55338	3.44701	4.67132
H	-7.98635	4.25553	3.15670
H	6.97945	3.85169	3.85370
H	7.43217	2.26147	4.48705
H	8.54635	3.13524	3.40319
H	9.03455	-2.96819	-3.81100
H	7.98695	-4.25472	-3.15879
H	7.55103	-3.44646	-4.67274
C	3.41151	-3.94506	0.43207
H	2.88808	-3.19462	1.04134
H	3.28457	-4.92814	0.91078
H	4.48070	-3.67924	0.45000
C	2.75057	-1.59194	-3.98681
H	3.81631	-1.37064	-4.16193
H	2.26623	-1.72953	-4.96418
H	2.31228	-0.70128	-3.50865
C	-2.61665	-3.25349	-1.42325
H	-3.66553	-3.57012	-1.55204
H	-1.97814	-4.14637	-1.49737
H	-2.52774	-2.85334	-0.40174
C	-2.64365	1.51503	-3.17483
H	-3.66082	1.61245	-2.77022
H	-1.95799	2.02596	-2.47983
H	-2.59018	2.02385	-4.15037
C	-3.41316	3.94667	-0.43056
H	-2.88939	3.19765	-1.04126
H	-4.48203	3.67951	-0.44793
H	-3.28763	4.93048	-0.90812
C	-2.75084	1.59102	3.98687
H	-3.81650	1.36978	4.16258
H	-2.31315	0.70058	3.50777
H	-2.26593	1.72796	4.96404
C	2.61693	3.25370	1.42205
H	3.66597	3.57001	1.55022
H	2.52739	2.85323	0.40070
H	1.97873	4.14681	1.49614
C	2.64335	-1.51421	3.17542
H	3.66083	-1.61146	2.77154
H	2.58958	-2.02253	4.15121
H	1.95842	-2.02564	2.48006

I2’_SVP

75

Al	1.77532	-0.07982	-0.19965
O	4.70144	0.08512	0.33904
O	-0.13361	-0.35377	-0.99394
O	3.92660	-0.81872	-2.12896
N	1.09918	-1.54547	0.87675
N	1.12417	1.74426	-0.32088
C	3.57971	0.22458	0.68266
C	2.76946	-0.71765	-1.90179
C	-0.85446	0.81424	-1.18968
C	-0.13165	1.96598	-0.83485
C	-0.85914	-1.38545	-0.41630
C	-2.17808	0.75865	-1.57419
C	-0.14904	-2.04973	0.59921
C	-2.77904	-0.63722	-1.81559
C	-2.18057	-1.58344	-0.75959
C	1.94738	2.87269	-0.06795
C	-2.84190	-2.63774	-0.10047
H	-3.88561	-2.86187	-0.32568
C	1.82241	-2.07858	1.97442
C	-2.16405	-3.41197	0.85581
C	2.62475	3.49712	-1.14012
C	2.94904	-2.90028	1.74646
C	-0.81735	3.18361	-1.00975
H	-0.31457	4.12185	-0.76500
C	-4.30919	-0.61302	-1.74978
H	-4.66712	-0.27289	-0.76592
H	-4.72078	-1.61528	-1.94090
H	-4.72062	0.05758	-2.51891
C	-0.83350	-3.11996	1.20797
H	-0.33495	-3.70182	1.98643
C	-2.15305	3.19554	-1.45282
C	2.09176	3.35533	1.25071
C	-2.83563	1.99604	-1.71444
H	-3.88059	2.03318	-2.02585
C	3.67702	-3.36848	2.85118
H	4.55229	-4.00195	2.67734
C	1.42884	-1.75132	3.29547
C	-2.86280	-4.57506	1.52149
C	3.30139	-3.04581	4.15399
H	3.88203	-3.41875	5.00252
C	2.17769	-2.24419	4.37013
H	1.87979	-1.98309	5.39026
C	2.91238	4.46780	1.48000
H	3.02564	4.84323	2.50155
C	3.43760	4.60688	-0.87251
H	3.96744	5.08936	-1.69946
C	-2.85062	4.52182	-1.64837
C	-2.33293	-1.12501	-3.21777
H	-2.71395	-0.44639	-3.99802
H	-2.72053	-2.13846	-3.41046
H	-1.23576	-1.15577	-3.29475
C	3.58260	5.09413	0.42771
H	4.22137	5.96057	0.62130
H	-2.69989	-4.57337	2.61204
H	-3.94819	-4.55389	1.33828
H	-2.48072	-5.54014	1.14371
H	-2.64755	5.21097	-0.81221
H	-2.50148	5.02486	-2.56779
H	-3.94134	4.39792	-1.73260
C	3.38248	-3.26330	0.35005
H	4.05432	-2.49930	-0.07477
H	3.92923	-4.21922	0.34984
H	2.52392	-3.34917	-0.33095
C	0.23327	-0.86806	3.53569
H	0.26718	0.02428	2.89327
H	-0.70958	-1.38714	3.29556
H	0.18635	-0.54378	4.58611
C	1.39464	2.65978	2.38728
H	0.33302	2.47468	2.16153
H	1.86373	1.67600	2.56457
H	1.46232	3.24661	3.31586
C	2.47935	2.96522	-2.54167
H	3.08352	3.55237	-3.24944

H 2.80035 1.91316 -2.60725
H 1.43027 2.99405 -2.87894

I2_SVP

75
Al 1.83572 -0.36507 -0.52622
C 4.73300 -0.44788 -0.00588
O -0.10321 -0.45692 -1.17180
O 3.99403 -1.35672 -2.01832
N 1.06194 -1.51648 0.85059
N 1.41412 1.53900 -0.64506
O 3.62059 -0.14956 0.33154
C 2.78696 -1.26102 -2.11261
C -0.68809 0.78652 -1.40265
C 0.16901 1.86542 -1.13163
C -0.91307 -1.30920 -0.42167
C -2.02813 0.85850 -1.72532
C -0.24990 -1.88867 0.67372
C -2.78781 -0.47680 -1.82800
C -2.26631 -1.36837 -0.68656
C 2.41241 2.54622 -0.60903
C -3.02813 -2.20849 0.14571
H -4.10237 -2.31565 -0.01119
C 1.72992 -1.90774 2.03693
C -2.41178 -2.90966 1.19657
C 3.02036 2.97438 -1.81308
C 2.73612 -2.89862 1.98223
C -0.38757 3.14102 -1.35331
H 0.22591 4.02854 -1.18241
C -4.30279 -0.27400 -1.74049
H -4.59349 0.20300 -0.79218
H -4.82699 -1.23821 -1.81809
H -4.65805 0.35731 -2.56874
C -1.04124 -2.74602 1.46508
H -0.58876 -3.25383 2.31988
C -1.73063 3.27883 -1.74747
C 2.83352 3.07033 0.63168
C -2.55307 2.15034 -1.90973
H -3.60198 2.28855 -2.17626
C 3.43212 -3.22030 3.15610
H 4.21347 -3.98523 3.11592
C 1.41319 -1.27285 3.26212
C -3.22459 -3.85047 2.05563
C 3.13980 -2.58749 4.36463
H 3.69582 -2.84728 5.26988
C 2.13113 -1.62313 4.41296
H 1.89642 -1.12452 5.35847
C 3.84833 4.03643 0.65269
H 4.17385 4.44430 1.61444
C 4.02823 3.94600 -1.75248
H 4.50333 4.27598 -2.68129
C -2.28699 4.66092 -2.00034
C -2.43650 -1.14542 -3.18145
H -2.76545 -0.50829 -4.01822
H -2.93826 -2.12296 -3.26448
H -1.35270 -1.30643 -3.27718
C 4.44202 4.47919 -0.53036
H 5.23375 5.23304 -0.49976
H -2.97137 -3.74308 3.12311
H -4.30495 -3.67251 1.94007
H -3.03191 -4.90423 1.78590
H -1.95244 5.37786 -1.23289
H -1.94763 5.05469 -2.97507
H -3.38793 4.65899 -2.01036
C 2.61290 2.37089 -3.13189
H 3.21572 2.78483 -3.95391
H 2.74359 1.27546 -3.13161
H 1.55081 2.55914 -3.35872
C 3.05142 -3.60283 0.68889
H 3.64476 -2.97199 0.00755
H 3.62874 -4.52103 0.87573
H 2.13294 -3.86881 0.14390
C 0.31733 -0.24179 3.32805
H 0.34924 0.43202 2.46055
H -0.67835 -0.71713 3.31345
H 0.39336 0.35936 4.24664
C 2.20931 2.57626 1.90835
H 1.11007 2.60570 1.85522
H 2.48959 1.52761 2.09975
H 2.53654 3.17781 2.76975

CO_SVP

2
C -2.09828 -2.73860 0.03590
O -0.97332 -2.73860 0.03590

TS1_SVP

73
Al -2.62348 -0.80363 -1.41112
N -3.46708 -1.73527 0.23894
N -3.12733 1.16914 -1.01097
C -4.77906 -2.11169 0.13047
C -2.81976 -1.93267 1.47176
C -4.39451 1.58401 -1.31583
C -2.13838 2.18009 -0.92265
C -5.52599 -1.55538 -0.92454
C -5.51820 -2.95937 0.99206
C -1.69857 -2.79391 1.56297
C -3.25071 -1.21680 2.62136
C -5.32315 0.59620 -1.69617
C -4.92669 2.89655 -1.27183
C -1.47950 2.62328 -2.09568
C -1.80178 2.74962 0.32815
O -4.80494 -0.69476 -1.75135
C -6.90712 -1.54940 -1.01824
C -6.90839 -3.09506 0.86082
H -5.00072 -3.46251 1.81267
C -1.00257 -2.89067 2.77520
C -2.52789 -1.34249 3.81449
C -6.68826 0.75989 -1.83548
H -4.26626 3.72990 -1.02049
C -6.29477 3.13542 -1.48709
C -0.45362 3.57212 -1.98948
C -0.77235 3.69887 0.39633
C -7.51257 -0.53361 -2.00885
C -7.61107 -2.36545 -0.11646
C -7.65870 -4.02348 1.78779
H -0.13416 -3.55414 2.83586
C -1.40289 -2.16492 3.89821
H -2.85106 -0.77050 4.69023
C -7.18187 2.07362 -1.73904
C -6.81150 4.55538 -1.44271
H 0.06127 3.90150 -2.89733
C -0.08851 4.10242 -0.75087
H -0.50976 4.12700 1.36872
C -8.99946 -0.29424 -1.73442
C -7.34644 -1.05709 -3.45700
H -8.70032 -2.42227 -0.14884
H -7.16426 -4.10213 2.76912
H -8.69354 -3.68187 1.95067
H -7.71574 -5.04562 1.37127
H -0.84505 -2.24430 4.83557
H -8.24802 2.27907 -1.84748
H -6.34171 5.13136 -0.62863
H -6.58944 5.09196 -2.38289
H -7.90280 4.58556 -1.29743
H 0.71766 4.83845 -0.68235
H -9.16971 0.08138 -0.71422
H -9.57132 -1.22633 -1.85930
H -9.40964 0.43948 -2.44488
H -7.73551 -0.31789 -4.17603
H -7.90373 -1.99905 -3.58769
H -6.29029 -1.24627 -3.69437
C -0.65566 -0.03599 0.34420
O 0.48771 -0.04421 0.33565
C -1.90351 2.10470 -3.44543
H -1.29582 2.54984 -4.24796
H -1.80945 1.00751 -3.50068
H -2.96322 2.33896 -3.64393
C -2.54150 2.33486 1.56867
H -3.62990 2.43633 1.43980
H -2.34792 1.27677 1.80186
H -2.23403 2.94162 2.43414
C -4.45969 -0.31978 2.56371
H -4.52022 0.19903 1.59794
H -5.39243 -0.89901 2.66707
H -4.43245 0.42933 3.36941
C -1.26949 -3.61293 0.37320
H -0.53303 -4.37704 0.66669
H -2.13332 -4.11146 -0.09530
H -0.82009 -2.98628 -0.41485

TS2_SVP

75
Al 0.63244 0.52053 1.44011
O 3.52457 0.75049 2.12430
O -1.43823 0.31328 0.84149
O 5.24377 -1.18417 -3.14142
N -0.10548 -0.82154 2.73526
N -0.10039 2.38014 1.61083
C 2.38132 0.85816 2.40898
C 4.06791 -1.12589 -3.01912
C -2.14199 1.51149 0.80062
C -1.38249 2.62854 1.19959
C -2.12431 -0.69235 1.51305
C -3.49692 1.49403 0.53464
C -1.36978 -1.30924 2.52931
C -4.13597 0.11355 0.29115
C -3.47755 -0.85809 1.28895
C 0.75738 3.49046 1.80227
C -4.12319 -1.86022 2.03776
H -5.18734 -2.05802 1.90005
C 0.58442 -1.24256 3.89540
C -3.39970 -2.60856 2.98178

C	1.30041	4.16286	0.68067
C	1.69986	-2.10599	3.79117
C	-2.07415	3.86008	1.16280
H	-1.54757	4.77679	1.43857
C	-5.65681	0.15786	0.46606
H	-5.93911	0.48149	1.47938
H	-6.09704	-0.83416	0.28432
H	-6.10960	0.85230	-0.25762
C	-2.04475	-2.33183	3.23260
H	-1.51657	-2.87961	4.01656
C	-3.43933	3.91245	0.82970
C	1.08867	3.91216	3.11039
C	-4.15546	2.73870	0.54066
H	-5.22336	2.80291	0.32621
C	2.40101	-2.45642	4.95406
H	3.26493	-3.12320	4.87103
C	0.16796	-0.77140	5.16701
C	-4.07874	-3.72288	3.74456
C	2.01153	-1.97677	6.20479
H	2.57296	-2.25522	7.10120
C	0.89594	-1.14274	6.30402
H	0.58259	-0.76426	7.28215
C	1.96208	4.99512	3.27808
H	2.21670	5.31908	4.29198
C	2.16677	5.24473	0.88731
H	2.58957	5.76020	0.01929
C	-4.13649	5.25320	0.79139
C	-3.80528	-0.34499	-1.15134
H	-4.23123	0.36005	-1.88367
H	-4.22765	-1.34553	-1.33983
H	-2.71880	-0.39400	-1.31247
C	2.50173	5.66203	2.17678
H	3.18342	6.50482	2.32301
H	-3.79749	-3.71231	4.81054
H	-5.17544	-3.64886	3.67877
H	-3.78977	-4.71293	3.34827
H	-3.89566	5.85885	1.68094
H	-3.82301	5.84248	-0.08885
H	-5.23053	5.13966	0.74194
C	0.94655	3.71569	-0.71371
H	1.56094	4.23568	-1.46430
H	1.09365	2.62920	-0.82562
H	-0.11499	3.91259	-0.94322
C	0.51041	3.19544	4.29784
H	-0.58552	3.11875	4.22543
H	0.89897	2.16450	4.34695
H	0.76638	3.70920	5.23704
C	-1.04180	0.11701	5.29175
H	-1.09930	0.82256	4.45267
H	-1.97345	-0.47361	5.26618
H	-1.02359	0.68294	6.23557
C	2.13616	-2.64844	2.45367
H	2.73362	-1.90915	1.89588
H	2.75691	-3.54917	2.58119
H	1.27167	-2.89675	1.82027

TS2B_SVP

75

Al	1.80372	-0.23253	-0.43261
O	3.81536	0.34086	0.63037
O	-0.07113	-0.50327	-1.21175
O	3.86982	0.61556	-2.25488
N	1.03393	-1.57826	0.85070
N	1.06133	1.59165	-0.23283
C	4.77885	0.74324	0.10875
C	2.75108	0.18689	-2.28800
C	-0.77137	0.68974	-1.42268
C	-0.13225	1.81437	-0.87611
C	-0.85068	-1.46027	-0.56385
C	-2.07626	0.63572	-1.87329
C	-0.22004	-2.05337	0.54060
C	-2.66197	-0.76539	-2.12195
C	-2.15977	-1.64818	-0.96402
C	1.46909	2.56729	0.71194
C	-2.89713	-2.60770	-0.24946
H	-3.93646	-2.81306	-0.51043
C	1.89119	-2.38562	1.64030
C	-2.30346	-3.30895	0.81559
C	2.50997	3.47505	0.41899
C	2.34254	-3.63804	1.14908
C	-0.84648	3.02593	-0.99230
H	-0.40581	3.94415	-0.59769
C	-4.19111	-0.73895	-2.18895
H	-4.63269	-0.35393	-1.25728
H	-4.58636	-1.75032	-2.36744
H	-4.53145	-0.10461	-3.02118
C	-0.98402	-3.03100	1.21116
H	-0.55165	-3.55076	2.06947
C	-2.13628	3.05046	-1.54819
C	0.81044	2.61490	1.96644
C	-2.76079	1.86085	-1.96243
H	-3.78682	1.89576	-2.33138
C	3.21874	-4.40318	1.93003
H	3.56512	-5.36774	1.54603
C	2.36484	-1.90635	2.88389
C	-3.08690	-4.37598	1.54494
C	3.67205	-3.94297	3.16681
H	4.36226	-4.54795	3.76149
C	3.25002	-2.69640	3.62979
H	3.60904	-2.32213	4.59334
C	1.26140	3.51576	2.93870
H	0.76590	3.53877	3.91435
C	2.93325	4.36361	1.41930
H	3.74620	5.06158	1.19683
C	-2.85664	4.37066	-1.69227
C	-2.10325	-1.31983	-3.45648
H	-2.42837	-0.68479	-4.29629
H	-2.47083	-2.34458	-3.62753
H	-1.00406	-1.34238	-3.45052
C	2.32844	4.37837	2.67612
H	2.67646	5.07320	3.44587
H	-2.89197	-4.35055	2.62930
H	-4.17063	-4.25963	1.38841
H	-2.80938	-5.38577	1.19268
H	-2.64880	5.03813	-0.84050
H	-2.53207	4.90352	-2.60415
H	-3.94679	4.23206	-1.76409
C	3.13044	3.51951	-0.95141
H	3.86939	2.71742	-1.10193
H	2.37123	3.38001	-1.73517
H	3.63905	4.48227	-1.11477
C	-0.37936	1.72823	2.22898
H	-1.26687	2.09349	1.68490
H	-0.19944	0.70320	1.87814
H	-0.62505	1.70026	3.30114
C	1.92003	-0.56741	3.40499
H	2.23074	0.25232	2.73937
H	0.82371	-0.51353	3.47858
H	2.34584	-0.37219	4.40064
C	1.92011	-4.12782	-0.21106
H	2.01965	-3.32745	-0.96264
H	2.53480	-4.98330	-0.52856
H	0.86294	-4.44133	-0.22691

TS2B_SVP

75

Al	1.82262	-0.06427	-0.23343
C	4.28460	0.62542	0.04830
O	-0.12442	-0.15276	-0.86987
O	4.05291	-0.83389	-1.86690
N	1.31566	-1.79269	0.50787
N	1.11697	1.50201	0.64432
O	3.57556	0.04488	0.80559
C	2.85364	-0.76292	-1.97951
C	-0.90798	0.90120	-0.40843
C	-0.20303	1.80741	0.39898
C	-0.71538	-1.39834	-0.64834
C	-2.26742	0.88572	-0.64748
C	0.09561	-2.27831	0.08644
C	-2.81404	-0.32015	-1.43697
C	-2.06241	-1.55836	-0.91150
C	1.92553	2.51554	1.22682
C	-2.61804	-2.80743	-0.58245
H	-3.67084	-3.01483	-0.77940
C	1.90130	-2.40515	1.64557
C	-1.82418	-3.79520	0.02790
C	2.37245	3.59194	0.42659
C	3.12696	-3.10046	1.53870
C	-0.96856	2.88867	0.88470
H	-0.48870	3.64993	1.50378
C	-4.32876	-0.46414	-1.26923
H	-4.61000	-0.60340	-0.21437
H	-4.70099	-1.32569	-1.84375
H	-4.84540	0.42967	-1.65011
C	-0.48901	-3.52877	0.37421
H	0.08899	-4.27224	0.92787
C	-2.34741	2.96633	0.62072
C	2.31601	2.42061	2.57890
C	-3.00076	1.96197	-0.11745
H	-4.07936	2.02655	-0.26900
C	3.70740	-3.63581	2.69876
H	4.65582	-4.17506	2.61314
C	1.26081	-2.28122	2.90625
C	-2.41036	-5.15504	0.32771
C	3.09387	-3.50269	3.94333
H	3.56260	-3.92380	4.83739
C	1.87263	-2.83289	4.03875
H	1.38293	-2.72522	5.01157
C	3.15524	3.40682	3.11494
H	3.45938	3.33216	4.16336
C	3.20399	4.56445	0.99889
H	3.55512	5.39509	0.37918

C	-3.13345	4.15045	1.13526
C	-2.48476	-0.12656	-2.93876
H	-2.98012	0.77932	-3.32390
H	-2.83764	-0.99388	-3.51975
H	-1.40219	-0.02351	-3.10012
C	3.59829	4.47572	2.33407
H	4.25457	5.23700	2.76538
H	-2.03446	-5.55467	1.28339
H	-3.50986	-5.11984	0.37784
H	-2.14003	-5.88625	-0.45517
H	-2.76554	4.48387	2.11884
H	-3.04835	5.01344	0.45047
H	-4.20474	3.91405	1.23140
C	1.83692	1.27572	3.42747
H	0.74013	1.19453	3.39828
H	2.23239	0.31353	3.06769
H	2.14865	1.40175	4.47534
C	1.95413	3.69055	-1.01651
H	2.49792	4.49622	-1.53182
H	2.15113	2.74152	-1.54108
H	0.87313	3.89085	-1.11088
C	3.79936	-3.29077	0.20670
H	4.46176	-4.17018	0.22881
H	3.06243	-3.41779	-0.59908
H	4.40764	-2.41842	-0.07687
C	-0.06379	-1.57523	3.03866
H	-0.11637	-0.69089	2.38976
H	-0.89679	-2.23275	2.73855
H	-0.23791	-1.26028	4.07827

TS3_SVP

75

Al	1.89765	-0.13600	-0.37106
O	3.45112	0.13880	0.77328
O	0.05464	-0.43018	-1.29007
O	3.66435	-0.26106	-2.88266
N	1.45761	-1.92582	0.24852
N	0.90685	1.44457	0.24595
C	4.58134	-0.15726	0.40310
C	3.26591	-0.37081	-1.76202
C	-0.86902	0.58482	-1.05122
C	-0.37159	1.61414	-0.23526
C	-0.47305	-1.70520	-1.09657
C	-2.16889	0.44323	-1.49601
C	0.31782	-2.50731	-0.25731
C	-2.50853	-0.85595	-2.25052
C	-1.75030	-1.99075	-1.53775
C	1.56776	2.57921	0.78734
C	-2.24185	-3.27370	-1.23565
H	-3.23760	-3.57629	-1.56258
C	2.09711	-2.59083	1.33043
C	-1.45969	-4.17333	-0.49104
C	2.02760	3.59897	-0.08056
C	3.28745	-3.31981	1.11495
C	-1.27423	2.66725	0.01632
H	-0.94863	3.51566	0.62244
C	-4.01778	-1.11403	-2.27727
H	-4.43211	-1.20120	-1.26144
H	-4.24285	-2.04217	-2.82367
H	-4.54117	-0.29655	-2.79537
C	-0.20043	-3.79210	0.00414
H	0.36700	-4.47774	0.63731
C	-2.59365	2.61318	-0.46650
C	1.80643	2.66566	2.17588
C	-3.04749	1.49952	-1.19477
H	-4.08771	1.46364	-1.52191
C	3.92290	-3.91345	2.21585
H	4.85091	-4.47011	2.05412
C	1.52894	-2.50812	2.62340
C	-1.97204	-5.56675	-0.20912
C	3.38832	-3.80936	3.50008
H	3.90051	-4.27399	4.34756
C	2.19179	-3.11638	3.69747
H	1.76051	-3.04230	4.70057
C	2.48390	3.78325	2.68209
H	2.66647	3.84942	3.75894
C	2.69640	4.70379	0.46269
H	3.05500	5.49013	-0.20851
C	-3.52644	3.77124	-0.19660
C	-1.98621	-0.73991	-3.70463
H	-2.48538	0.09380	-4.22438
H	-2.18870	-1.67128	-4.25777
H	-0.90189	-0.55793	-3.72597
C	2.92413	4.80232	1.83643
H	3.45211	5.66814	2.24582
H	-1.78089	-5.86299	0.83537
H	-3.05418	-5.64605	-0.39557
H	-1.46988	-6.31349	-0.84973
H	-3.40545	4.15694	0.82871
H	-3.32389	4.61423	-0.88134
H	-4.58035	3.48288	-0.33218
C	1.35247	1.56232	3.09050
H	0.29002	1.32176	2.93334
H	1.92172	0.63902	2.89566
H	1.49769	1.83729	4.14616
C	0.21220	-1.80732	2.83359
H	-0.62728	-2.44266	2.50243
H	0.05520	-1.56348	3.89495
H	0.15035	-0.88253	2.24524
C	3.84817	-3.45938	-0.27111
H	4.71429	-4.13834	-0.28245
H	3.08765	-3.84727	-0.96888
H	4.15658	-2.47405	-0.67324
C	1.82328	3.48396	-1.56922
H	2.20395	2.52233	-1.95071
H	0.75477	3.52612	-1.83958
H	2.34168	4.29581	-2.10065

TS4_SVP

150

Al	3.57554	-0.25039	-0.63875
O	5.54233	0.43002	-1.04405
O	1.97262	-1.15277	-0.20988
O	1.69088	1.16722	-2.69803
N	4.40333	-1.78581	-1.57358
N	4.09734	0.57225	1.06662
C	6.34757	-0.46801	-1.73126
C	5.69297	-1.67991	-2.02170
C	6.47515	-2.63575	-2.70316
H	6.02830	-3.59419	-2.97683
C	7.82972	-2.38593	-2.98651
C	8.43914	-1.18694	-2.58312
H	9.50115	-1.03410	-2.78064
C	7.68694	-0.19579	-1.92302
C	8.21450	1.14326	-1.38109
C	7.49515	1.40791	-0.04708
C	8.05321	2.00266	1.10072
H	9.09589	2.32397	1.10550
C	7.27106	2.19058	2.25212
C	5.93430	1.75738	2.29230
H	5.34664	1.87847	3.20516
C	5.34380	1.13234	1.17536
C	6.16592	1.04414	0.03595
C	7.86491	2.85345	3.47398
C	7.83206	2.26665	-2.37801
H	6.74313	2.31060	-2.52578
H	8.30461	2.08630	-3.35733
H	8.16739	3.24555	-1.99838
C	9.73642	1.12214	-1.20799
H	10.09701	2.08983	-0.82827
H	10.23472	0.94646	-2.17325
H	10.05255	0.33519	-0.50646
C	8.62989	-3.42948	-3.73164
C	3.77076	-3.05359	-1.67659
C	2.83082	-3.30534	-2.69817
C	2.18807	-4.55220	-2.73432
H	1.45233	-4.74512	-3.52110
C	2.47046	-5.53743	-1.78944
H	1.95427	-6.50101	-1.82805
C	3.41628	-5.28653	-0.79265
H	3.64338	-6.05466	-0.04686
C	4.07664	-4.05417	-0.72405
C	3.11882	0.87730	2.05507
C	2.65316	-0.13325	2.92271
C	1.65284	0.17878	3.85337
H	1.26858	-0.61132	4.50451
C	1.12606	1.46664	3.93744
H	0.32949	1.68890	4.65258
C	1.58506	2.45793	3.07082
H	1.13929	3.45641	3.10081
C	2.56785	2.17928	2.11358
C	1.12107	-0.65208	-1.04444
C	2.03672	0.46000	-1.77477
O	-1.99280	1.23771	0.20139
O	-1.66120	-1.08872	2.67756
C	-1.13049	0.75102	1.03371
C	-2.02040	-0.38742	1.75478
Al	-3.57407	0.29441	0.62309
O	-5.52182	-0.43524	1.02940
N	-4.43346	1.80103	1.57813
N	-4.08365	-0.52454	-1.08871
C	-6.34608	0.44111	1.72598
C	-5.71381	1.65887	2.03844
C	-6.50920	2.58508	2.74541
H	-6.07738	3.54515	3.03726
C	-7.85657	2.30317	3.03215
C	-8.44549	1.10158	2.60633
H	-9.50276	0.92334	2.80807
C	-7.67829	0.13851	1.92259
C	-8.18002	-1.20494	1.36916
C	-7.46396	-1.43412	0.02784
C	-8.01734	-2.01957	-1.12623

H	-9.05411	-2.35940	-1.13059
C	-7.23863	-2.17413	-2.28494
C	-5.91049	-1.71628	-2.32397
H	-5.32543	-1.81408	-3.24107
C	-5.32560	-1.09985	-1.19945
C	-6.14181	-1.04357	-0.05619
C	-7.76343	-2.33143	2.34878
H	-6.67264	-2.35178	2.48753
H	-8.23226	-2.17453	3.33390
H	-8.07900	-3.31303	1.95928
C	-9.70321	-1.21765	1.20775
H	-10.04303	-2.18762	0.81487
H	-10.19794	-1.06990	2.17951
H	-10.04405	-0.42742	0.52163
C	-3.69780	2.94162	1.99704
C	-3.09161	2.96372	3.27335
C	-2.30232	4.06655	3.62802
H	-1.81765	4.07770	4.60892
C	-2.12152	5.13555	2.74928
H	-1.49306	5.98314	3.03718
C	-2.74991	5.11751	1.50306
H	-2.62170	5.95718	0.81313
C	-3.54539	4.03154	1.11514
C	-3.11033	-0.87076	-2.07014
C	-2.63031	0.09819	-2.97585
C	-1.67831	-0.27813	-3.93349
H	-1.29082	0.47851	-4.62151
C	-1.21421	-1.58968	-4.00885
H	-0.47172	-1.86709	-4.76093
C	-1.66853	-2.53568	-3.08972
H	-1.26661	-3.55306	-3.10945
C	-2.60015	-2.19153	-2.10375
C	-7.82691	-2.82728	-3.51466
H	-7.29308	-3.76019	-3.76643
H	-8.88994	-3.07607	-3.37259
H	-7.74647	-2.16927	-4.39677
C	-8.67177	3.31430	3.80504
H	-9.74557	3.07181	3.78243
H	-8.36099	3.35254	4.86430
H	-8.54206	4.33155	3.39926
H	7.34309	3.79696	3.71093
H	7.77333	2.20941	4.36532
H	8.93160	3.08575	3.33136
H	9.70787	-3.20630	-3.70869
H	8.47915	-4.43448	-3.30320
H	8.32412	-3.48662	-4.79150
C	5.09738	-3.78626	0.35023
H	4.88126	-2.84720	0.88047
H	5.12067	-4.60429	1.08586
H	6.10908	-3.67858	-0.07617
C	2.49536	-2.25740	-3.72466
H	2.17125	-2.72470	-4.66793
H	1.66405	-1.62244	-3.37681
H	3.34725	-1.59401	-3.92753
C	-3.02015	-3.20031	-1.06662
H	-4.07478	-3.50298	-1.18210
H	-2.39635	-4.10434	-1.12778
H	-2.91947	-2.78003	-0.05297
C	-3.09858	1.52458	-2.89163
H	-4.17966	1.58801	-2.69525
H	-2.58426	2.03420	-2.06042
H	-2.87104	2.07291	-3.81852
C	-3.29247	1.82583	4.23997
H	-4.35630	1.71487	4.51027
H	-2.97276	0.86529	3.81241
H	-2.72030	1.99472	5.16452
C	3.19803	-1.53288	2.82240
H	4.29812	-1.53587	2.76689
H	2.88805	-2.13956	3.68693
H	2.82172	-2.02520	1.91078
C	2.98959	3.23160	1.12131
H	4.03990	3.53913	1.26117
H	2.90571	2.84999	0.09066
H	2.35596	4.12653	1.20919
C	-4.23374	4.01120	-0.22236
H	-3.83359	3.20920	-0.85909
H	-5.31356	3.81894	-0.11356
H	-4.10029	4.96665	-0.75216

7 References:

S1a Hicks, J.; Vasko, P.; Goicoechea, J. M.; Aldridge, S. Synthesis, structure and reaction chemistry of a nucleophilic aluminyl anion. *Nature*, **2018**, *557*, 92-95.

S1b Roy, M. M. D.; Hicks, J.; Vasko, P.; Heilmann, A.; Baston, A.-M.; Goicoechea, J. M.; Aldridge, S. Probing the Extremes of Covalency in M−Al bonds: Lithium and Zinc Aluminyl Compounds. *Angew. Chem. Int. Ed.* **2021**, *60*, 22301.

S1c Cruz, C. A.; Emslie, D. J. H; Harrington, L. E.; Britten, J. F.; Robertson, C. M. Extremely Stable Thorium(IV) Dialkyl Complexes Supported by Rigid Tridentate 4,5-Bis(anilido)xanthene and 2,6-Bis(anilidomethyl)pyridine Ligands. *Organometallics* **2007** *26* (3), 692-701

S2 Hill-Cousins, J. T.; Pop, I.; Pileio, G.; Stevanato, G.; Håkansson, P.; Roy, S. S.; Levitt, M. H.; Brown, L. J.; Brown, R. C. D. Synthesis of an Isotopically Labeled Naphthalene Derivative That Supports a Long-Lived Nuclear Singlet State. *Organic Letters*, **2015**, *17*, (9), 2150-2153.

S3 Cosier, J.; Glazer, A. M. A nitrogen-gas-stream cryostat for general X-ray diffraction studies. *J. Appl. Cryst.* **1986**, *19*, 105-107.

S4 CrysAlisPro, Agilent Technologies, Version 1.171.39.46.

S5 Sheldrick, G. M. SHELXT - Integrated space-group and crystal-structure determination. *Acta Crystallogr., Sect. A: Found. Adv.,* **2015**, *71*, 3–8.

S6 Sheldrick, G. M. Crystal structure refinement with SHELXL. *Acta Crystallogr., Sect. C: Struct. Chem.,* **2015**, *71*, 3–8.

S7 Dolomanov, O. V.; Bourhis, L. J.; Gildea, R. J.; Howard, J. A. K.; Puschmann, H. OLEX2: a complete structure solution, refinement and analysis program. *J. Appl. Cryst.* **2009**, *42*, 339-341.

S8 Barbour, L. J. X-Seed — A Software Tool for Supramolecular Crystallography. *J. Supramol. Chem.* **2001**, 1189–1191.

S9 van der Sluis, P.; Spek, A. L. BYPASS: an effective method for the refinement of crystal structures containing disordered solvent regions. *Acta Cryst.,* **1990**, *A46*, 194-201.

S10 Spek, A. L. PLATON SQUEEZE: a tool for the calculation of the disordered solvent contribution to the calculated structure factors. *Acta Cryst.*, **2015**, *C71*, 9-18.

S11 Gaussian16, Revision C.01, Frisch, M. J.; Trucks, G. W.; Schlegel, H. B.; Scuseria, G. E.; Robb, M. A.; Cheeseman, J. R. ; Scalmani, G.; Barone, V.; Petersson, G. A.; Nakatsuji, H.; Li, X.; Caricato, M.; Marenich, A. V.; Bloino, J.; Janesko, B. G.; Gomperts, R.; Mennucci, B.; Hratchian, H. P.; Ortiz, J. V.; Izmaylov, A. F. ; Sonnenberg, J. L.; Williams-Young, D.; Ding, F.; Lipparini, F.; Egidi, F; Goings, J.; Peng, B.; Petrone, A.; Henderson, T.; Ranasinghe, D.; Zakrzewski, V. G.; Gao, J.; Rega, N.; Zheng, G.; Liang, W.; Hada, M.; Ehara, M.; Toyota, K.; Fukuda, R.; Hasegawa, J.; Ishida, M.; Nakajima, T.; Honda, Y.; Kitao, O.; Nakai, H. ; Vreven, T.; Throssell, K.; Montgomery, Jr., J. A.; Peralta, J. E.; Ogliaro, F.; Bearpark, M. J.; Heyd, J. J.; Brothers, E. N.; Kudin, K. N.; Staroverov, V. N.; Keith, T. A.; Kobayashi, R.; Normand, J. ; Raghavachari, K.; Rendell, A. P.; Burant, J. C.; Iyengar, S. S.; Tomasi, J.; Cossi, M.; Millam, J. M.; Klene, M.; Adamo, C.; Cammi, R.; Ochterski, J. W.; Martin, R. L.; Morokuma, K.; Farkas, O.; Foresman, J. B.; Fox, D. J. Gaussian, Inc., Wallingford CT, **2019**.

S12a Neese, F. The ORCA program system, Wiley Interdiscip. Rev.: Comput. Mol. Sci. **2012**, 2, 73–78.

S12b Neese, F. Software update: the ORCA program system, version 4.0, Wiley Interdiscip. Rev.: Comput. Mol. Sci. **2017**, 8, e1327.

S12c Neese, F.; Wennmohs, F.; Becker, U.; Riplinger, C. The ORCA quantum chemistry program package, J. Chem. Phys. **2020**, 152, 224108.

S13 Becke, A. D.; Density-functional thermochemistry. III. The role of exact exchange. *J. Chem. Phys.* **1993**, *98*, 5648–5652.

S14 Lee, C.; Yang, W.; Parr, R. G. Development of the Colle-Salvetti correlation-energy formula into a functional of the electron density. *Phys. Rev. B*, **1988**, *37*, 785–789.

S15 Vosko, S. H.; Wilk, L.; Nusair, M. Accurate spin-dependent electron liquid correlation energies for local spin density calculations: a critical analysis. *Can. J. Phys.* **1980**, *58*, 1200–1211.

S16 Stephens, P. J.; Devlin, F. J.; Chabalowski, C. F., Frisch, M. J. Ab Initio Calculation of Vibrational Absorption and Circular Dichroism Spectra Using Density Functional Force Fields. *J. Phys. Chem.* **1994**, *98*, 45, 11623–11627.

S17 Schaefer, A.; Huber, C.; Ahlrichs, R. Fully optimized contracted Gaussian basis sets of triple zeta valence quality for atoms Li to Kr. *J. Chem. Phys.* **1994**, *100*, 5829–5835.

S18 Schaefer, A.; Horn, H.; Ahlrichs, R. Fully optimized contracted Gaussian basis sets for atoms Li to Kr. *J. Chem. Phys.* **1992**, *97*, 2571–2577.

S19 Grimme, S.; Antony, J.; Ehrlich, S.; Krieg, H. A consistent and accurate ab initio parametrization of density functional dispersion correction (DFT-D) for the 94 elements H-Pu. *J. Chem. Phys.* **2010**, *132*, 154104–154119.

S20 Grimme, S.; Ehrlich, S.; Goerigk, L. Effect of the damping function in dispersion corrected density functional theory. *J. Comput. Chem.* **2011**, *32*, 1456–1465.

S21 Pople, J. A.; Segal, G. A. Approximate Self-Consistent Molecular Orbital Theory. III. CNDO Results for AB2 and AB3 Systems. J. Chem. Phys. **1965**, 43, 136.

S22 Pople, J. A.; Segal, G. A. Approximate Self-Consistent Molecular Orbital Theory. II. Calculations with Complete Neglect of Differential Overlap. J. Chem. Phys. **1966**, 44, 3289.

S23 Grimme, S. A generally applicable atomic-charge dependent London dispersion correction. J. Chem. Phys. **2019**, 150, 154122

S24 Cammi, R.; Mennucci, B.; Tomasi, J.; Fast Evaluation of Geometries and Properties of Excited Molecules in Solution: A Tamm-Dancoff Model with Application to 4-Dimethylaminobenzonitrile. J. of Phys. Chem. A. **2000,** 104(23), 5631.

S25a Tian Lu, Feiwu Chen, Multiwfn: A Multifunctional Wavefunction Analyzer, J. Comput. Chem. **2012**, 33, 580-592

S25b Tian Lu, Feiwu Chen, Calculation of Molecular Orbital Composition, Acta Chim. Sinica, **2011**, 69, 2393-2406
